# Effects of student human rights ordinances on mental health among middle and high school students in South Korea: a difference-in-differences analysis

**DOI:** 10.4178/epih.e2025011

**Published:** 2025-03-01

**Authors:** Sang Jun Eun

**Affiliations:** Department of Preventive Medicine, Chungnam National University College of Medicine, Daejeon, Korea

**Keywords:** Human rights, Mental health, Adolescent, Econometric models, Difference-in-differences, Republic of Korea

## Abstract

**OBJECTIVES:**

To actively protect and enhance students’ human rights, student human rights ordinances (SHROs) have been enforced in seven provinces in South Korea at different times since 2010. Although human rights are closely linked to mental health, there has been no research on the effectiveness of human rights legislation on adolescent mental health. This study evaluated the effects of SHROs on the mental health of middle and high school students.

**METHODS:**

Repeated cross-sectional data were used, including 1,148,257 respondents from the Korea Youth Risk Behavior Web-based Survey between 2006 and 2023. Probabilities of perceived stress, sleep insufficiency, depressive mood, suicide ideation, and suicide attempt in treated provinces were estimated through a difference-in-differences approach that accounts for treatment effect heterogeneity across groups over time.

**RESULTS:**

SHROs had no consistently significant effects on any mental health outcomes, except for slightly increased suicide ideation in total students (0.7%, 95% confidence interval 0.3% to 1.1%). Suicide attempts in total and male students and perceived stress and sleep insufficiency in female students tended to decrease, while other mental health outcomes tended to increase. Uncertainty in the effect estimates of SHROs increased for all mental health outcomes with possible violations of parallel trends, rendering originally significant effects insignificant.

**CONCLUSIONS:**

SHROs failed to improve mental health of middle and high school students in treated provinces, possibly due to the absence of enforcement mechanisms. Further research is needed on the effectiveness of and effect mechanisms for legal measures to improve human rights on adolescent mental health.

## GRAPHICAL ABSTRACT

[Fig f5-epih-47-e2025011]

## Key Message

Although student human rights ordinances have been enforced in South Korea to comprehensively guarantee human rights for students, they failed to improve the mental health of middle and high school students in treated provinces, possibly due to the absence of enforcement mechanisms such as penalty provisions. Despite the ineffectiveness of student human rights ordinances, this study first estimated the effects of human rights legislation on adolescent mental health. Further research is needed on the effectiveness of and effect mechanisms for legal measures to improve human rights on adolescent mental health.

## INTRODUCTION

Mental health is a cornerstone of adolescent health and development. However, mental health conditions are a major contributor to adolescent health burden, with 21.5 million disability-adjusted life years in 2019 and a prevalence of 9% for mental disorders, 17% for suicide ideation, 17% for suicide attempts, and 34% for depressive symptoms [1-4]. Human rights are closely linked to mental health, as human rights violations worsen mental health and mental health depends on the extent to which human rights are guaranteed [5]. Children and adolescents are a vulnerable group that are prone to poor mental health and human rights violations [5].

In South Korea (hereinafter, Korea), adolescents have faced high rates of poor mental health, including suicide and suicide ideation [6,7]. In the past decade, suicide has been the leading cause of death, and mental health problems such as perceived stress, depressive mood, and suicide behaviors have worsened [8,9]. Along with mental illness and peer/family relationships, academic stress has been identified as the culprit of poor mental health in adolescents, being experienced by 69% of adolescents who died by suicide and the main immediate trigger for suicide during 2015-2021, and accounting for 63% of suicide ideation, 62% of felt stress, 39%-56% of sleep insufficiency, and 66% of unhappiness in 2023 [8-10]. The strong social pressure toward academic excellence in Korea has compromised the human rights of adolescents, not limited to mental health, including infringements of the right to health and the right to rest and leisure as individual consequences of academic stress, and discrimination and violence based on academic performance, parental socioeconomic status, and gender as contextual consequences of the highly-competitive educational environment [11-15].

The Constitution of the Republic of Korea guarantees all citizens fundamental human rights, and Korea ratified the United Nations Convention on the Rights of the Child (CRC) in 1991. Accordingly, the Framework Act on Education in 1997 and the Elementary and Secondary Education Act in 2007 stipulated that the human rights of students provided in the Constitution and international human rights treaties shall be guaranteed. Despite these legal provisions, human rights for students were not incorporated effectively in school education [16]. Therefore, to actively protect and enhance students’ human rights, provincial education offices in seven of 17 provinces, starting with Gyeonggi in 2010, enacted student human rights ordinances (SHROs) based on the Constitution, the CRC, and the related Acts [16]. Although SHROs were not enacted simultaneously across provinces, the substantive and procedural guarantees for students’ human rights were largely similar, encompassing freedom from discrimination and violence, the right to health, the right to learn, rest, and leisure, freedom of privacy, expression, conscience, and religion, the right to choose educational activities, human rights education for students and guardians, and the right to participate in school policy decisions. Details on SHROs are presented in Supplementary Material 1.

Social contexts that threaten human rights are related to worse mental health and may have generalized effects on mental health risk even in those not directly exposed to contextual stressors [17]. As a foundational context, legislation may influence human rights violations and mental health by mitigating contextual stressors [17,18]. Accordingly, SHROs that comprehensively protect students’ human rights may have the potential to improve students’ mental health. However, only few studies evaluated the effectiveness of human rights law on child health, all of which were ecological studies of the effects of international human rights treaties on health outcomes such as child mortalities and vaccination rates, and their findings were mixed with inconsistent improvements and inconclusive due to the lack of appropriate control groups [19-21]. Furthermore, the effectiveness of human rights law on mental health for adolescents has never been assessed, particularly at the subnational level. Therefore, this study aimed to examine the effects of staggered implementation of SHROs on the mental health of middle and high school students in Korea under a difference-in-differences (DiD) design.

## METHODS

### Data

Data from the Korea Youth Risk Behavior Web-based Survey (KYRBS) between 2006 and 2023 were used, which included nationally representative, annual cross-sectional samples of middle and high school students collected by the Korea Disease Control and Prevention Agency [8,22]. Participants were selected by multi-stage stratified cluster random sampling and self-reported online on health-risk behaviors, including smoking, drinking, physical activity, and mental health, with response rates ranging from 90.9% to 97.7% [8,22]. KYRBS was conducted around September during 2006-2011, around June during 2012-2019, and around September and October during 2020-2023 [8]. Of the 1,191,684 respondents in the 2006-2023 KYRBS, 1,148,257 (96.4%) students in grades 7-12 were included in the analysis, after excluding 8,623 from Sejong, one of the 17 provinces, due to missing years and 34,804 with missing values for study variables.

### Variables

**Figure 1.**
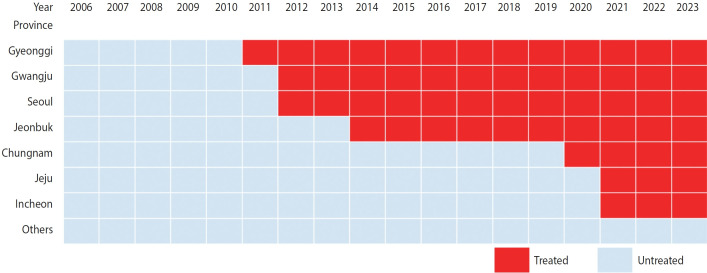
Difference-in-differences design with multiple groups and multiple time periods.

The intervention variable was the earliest year when KYRBS was conducted following the first enforcement of SHRO, corresponding to the year when a province first enforced SHRO. The treated group included the 2011 cohort of Gyeonggi, the 2012 cohort of Seoul and Gwangju, the 2014 cohort of Jeonbuk, the 2020 cohort of Chungnam, and the 2021 cohort of Incheon and Jeju (Figure 1, Supplementary Material 1). The never-treated comparison group included Busan, Daegu, Daejeon, Ulsan, Gangwon, Chungbuk, Jeonnam, Gyeongbuk, and Gyeongnam.

Outcome variables were perceived stress, depressive mood, sleep insufficiency, suicide ideation, and suicide attempt. Perceived stress was defined as those who felt a lot or very much stress in their daily lives. Sleep insufficiency was defined as those who thought that their sleep time in the previous 7 days was not enough or very not enough to recover from fatigue. Depressive mood was defined as those who had ever felt so sad or hopeless enough to stop their daily lives over the two weeks in the previous 12 months. Suicide ideation was defined as those who had ever seriously considered suicide in the previous 12 months. Suicide attempt was defined as those who had attempted suicide in the previous 12 months.

Potential covariates included sex (male, female), grade (7th to 12th), age (12 to 18 years), self-rated health (good, poor), perceived body image (lean, fat), eating breakfast (0 to 6-7 day/wk), eating fast food (0 to ≥ 3 times/wk), vigorous physical activity (0 to ≥ 3 day/wk), muscle-strengthening activity (0 to ≥ 3 day/wk), body mass index (normal/underweight, obesity/overweight), alcohol use (yes, no), cigarette or nicotine use (yes, no), academic performance (high, middle, low), family socioeconomic status (high, middle, low), living arrangement (living with family, living with non-family), area type (non-metropolitan, metropolitan), and outcome variables not used as a regressand.

All study variables except the intervention variable and area type were measured by self-report, which included only those variables measured in KYRBS throughout the study period. The study variables are detailed in Supplementary Material 2.

### Statistical analysis

Recent econometric literature has pointed out that standard two-way fixed effects (TWFE) models can yield biased estimates of the average treatment effect in a DiD design with multiple time periods and different treatment timings due to treatment effect heterogeneity across cohorts over time, such as the negative weight problem, where TWFE estimates derived from weighted averages of individual treatment effects can be negative when the treatment effect is positive across all units [23-25]. Therefore, the effects on adolescent mental health of SHROs implemented at different times were estimated with increasingly used DiD analysis for a staggered adoption setting developed by Callaway & Sant’Anna [23] (CSDiD), which allows for treatment effect heterogeneity and dynamic effect, thereby avoiding pitfalls in TWFE models [23-25].

The CSDiD approach estimates group-time average treatment effects, defined as the average treatment effect on the treated (ATT) for each group at each time, where groups are defined by the time at which a group first becomes treated, and then grouptime ATTs are aggregated into overall treatment effect parameters by averaging with weights proportional to the group size, across groups, across time periods, and across event times [23]. Grouptime ATTs in the seven treated provinces were estimated relative to both never-treated and not-yet-treated comparison units using the improved doubly robust DiD estimator based on weighted least squares models that adjusted for pre-treatment covariates with inverse probability tilting, which is robust for the ATT when either outcome regression or inverse probability tilting estimator is misspecified [23,26]. The covariates included a set of potential covariates that made the pre-treatment trends of the treatment and comparison groups as parallel as possible, and are shown for each outcome variable in Supplementary Material 3. The aggregated treatment effects of SHROs were presented as global (i.e., average of all group-time ATTs weighted by group size) and dynamic effects (i.e., ATTs by length of treatment). All models used wild bootstrapped standard errors clustered at the provincial level. Whether linear pre-treatment trends were parallel was examined by the Wald test.

To assess the robustness to analytical choices and potential biases, four sets of sensitivity analyses were conducted, including the same covariates as in the main CSDiD analyses for the first to third analyses. First, the comparison group was restricted to never-treated provinces that had never implemented a SHRO, excluding not-yet-treated units. Second, asymptotic standard errors were used rather than wild cluster bootstrapped standard errors. Third, the effects of SHROs were estimated using TWFE models. Fourth, sensitivity analyses outlined by Rambachan & Roth [27] were performed, which allow robust inference for the dynamic treatment effect in cases where the parallel trends assumption may not hold [27]. The robustness of the dynamic treatment effects of SHROs to potential violations of parallel trends was estimated by imposing bounds on relative magnitudes and smoothness restrictions, where the former bounds the maximum post-treatment violations of parallel trends between consecutive periods by *M̅* times the maximum pre-treatment violations, and the latter bounds the maximum change in the linear slope of the pre-treatment trend across consecutive periods by Μ, allowing for non-linearity in the counterfactual difference in trends [24,27]. Here, *M̅* ranged from 0.5 to 2.0 with an interval of 0.5 and Μ ranged from 0.00 to 0.05 with an interval of 0.01. As *M̅* or Μ increases, the degree of violation of the parallel trends increases. If a significant effect estimate of an intervention remains significant until *M̅* is 1.5 and becomes insignificant when Μ is 2.0, it suggests that the significant effect estimate in the main analysis is robust up to 1.5 times the maximum violation of the parallel trends in the pre-treatment period [27]. The effects of potential violations of parallel trends were estimated for the overall post-treatment dynamic effects, yielding 95% robust confidence intervals [27].

Analyses were performed following recent recommendations for staggered DiD analysis [24,25]. Sample weights were applied. CSDiD models, TWFE models, and sensitivity analyses proposed by Rambachan & Roth [27] were conducted in Stata 15.1 using the *csdid2, reghdfe*, and *HonestDiD* modules, respectively [28-30]. The basic structures of Stata commands for analyses were as follows: *csdid2 outcome covariates [weight = W], time(year) gvar (intervention) notyet cluster(province) method(drimp)* for CSDiD models, and *reghdfe outcome iv covariates [pweight= W], absorb (province year) vce(cluster province)* for TWFE models, where *iv* was coded 1 for the post-treatment period in the treated group and 0 otherwise and W was the sample weight.

## RESULTS

The descriptive characteristics of Korean middle and high school students in the treated and comparison groups are presented in Table 1, and by sex in Supplementary Material 4. All characteristics had statistically significant differences between the treated and comparison groups (p < 0.001), but the differences were generally not large, with students in the treated group reporting worse mental health, eating habits, and physical activity levels, and living more in metropolitan areas.

**Figure 2.**
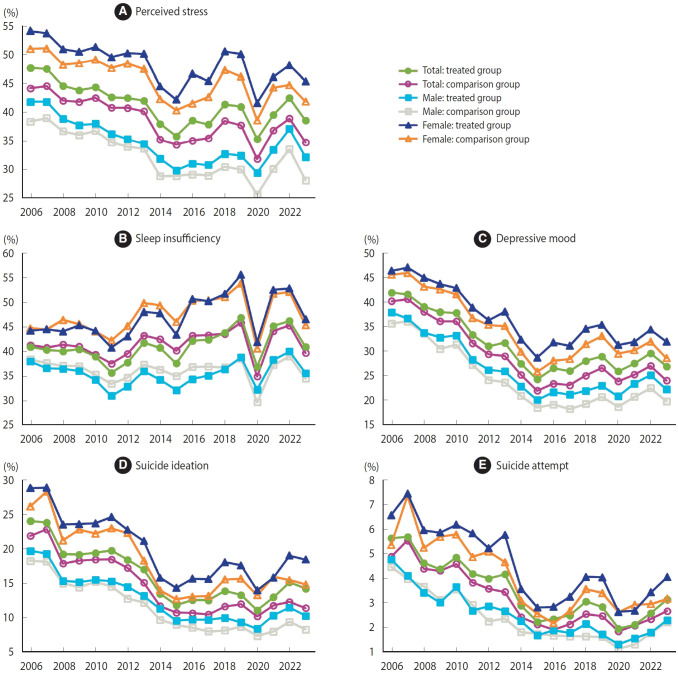
Trends in mental health among middle and high school students in South Korea by treatment status, 2006-2023: (A) perceived stress, (B) sleep insufficiency, (C) depressive mood, (D) suicide ideation, and (E) suicide attempt.

Trends in mental health among Korean middle and high school students are depicted in Figure 2 according to whether SHROs were enforced or not. Mental health was worse in the treated group than in the comparison group in most periods, except for sleep insufficiency. Trends in mental health did not seem to differ markedly between the treated and comparison groups, when visually inspected. However, pre-treatment parallel trends analyses based on CSDiD models rejected the null hypothesis that parallel trends held in the pre-treatment periods, except for suicide ideation in female students (Supplementary Material 5).

Table 2 shows the global effects of SHROs on mental health in Korean middle and high school students, which were minimal, with less than 1% change in probability of having a mental health problem, and statistically insignificant for all mental health outcomes, except for increases of 0.7% (95% confidence interval 0.3% to 1.1%) and 0.8% (95% confidence interval 0.3% to 1.2%) in suicide ideation among total and male students, respectively. In the treated group, following the implementation of SHROs, suicide attempts in total and male students and perceived stress and sleep insufficiency in female students tended to decrease, while other mental health outcomes tended to increase.

**Figure 3.**
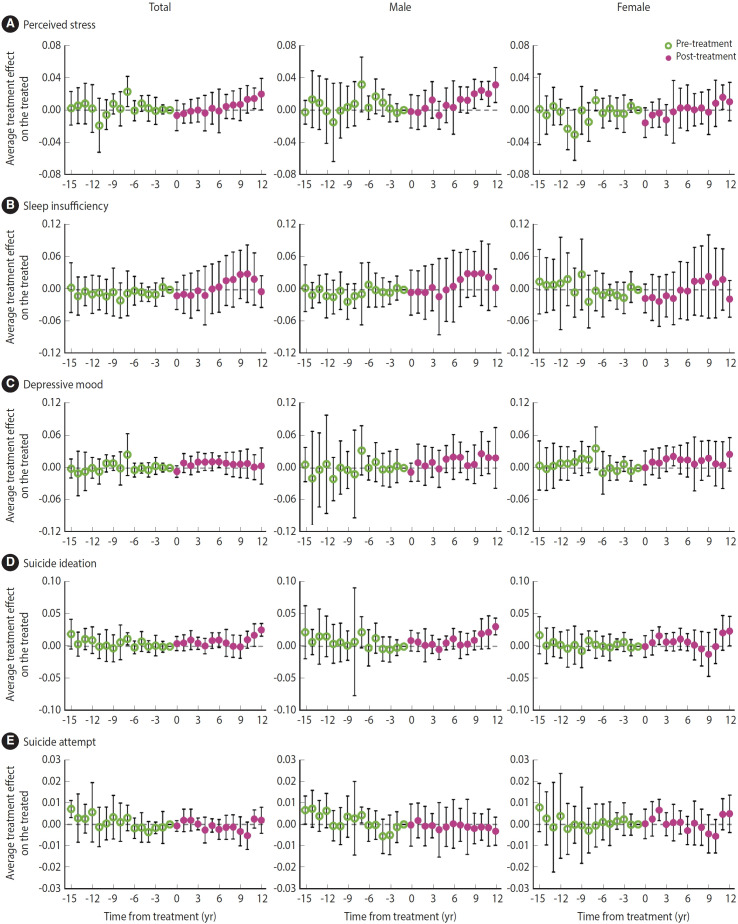
Dynamic effects of student human rights ordinances on mental health among middle and high school students in South Korea: (A) perceived stress, (B) sleep insufficiency, (C) depressive mood, (D) suicide ideation, and (E) suicide attempt.

The dynamic effects of SHROs are illustrated in Figure 3 and their coefficients are listed in Supplementary Material 6, demonstrating inconsistent patterns across mental health outcomes and sex. The effects of SHROs were not significant for most event time points, but were significant at some event times, such as increases in perceived stress at 11 and 12 years post-treatment, in depressive mood at 6 years post-treatment, and in suicide ideation at 12 years post-treatment. The overall dynamic effects were similar to the global effects, with a significant effect on suicide ideation in total students.

The overall group and time effects and their significances were also similar to the global effects, and group- and time-specific ATTs were heterogeneous across treated cohorts and time periods (Supplementary Materials 7 and 8). The group-time average treatment effects are reported in Supplementary Material 9.

**Figure 4.**
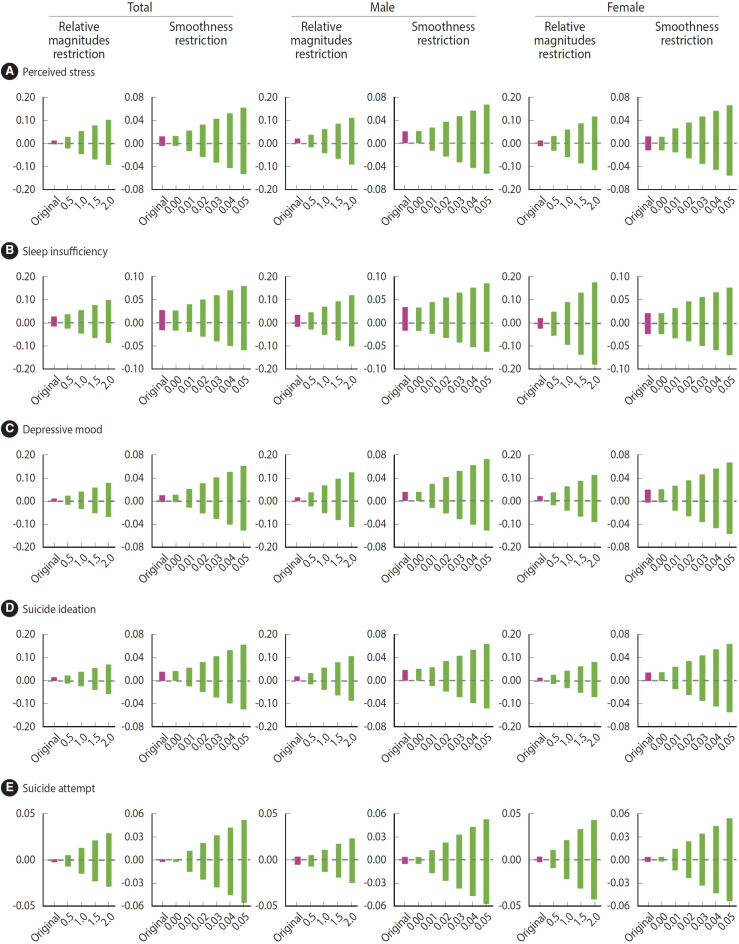
Robust confidence intervals for overall dynamic effects of student human rights ordinances by type and degree of potential violations of parallel trends: (A) perceived stress, (B) sleep insufficiency, (C) depressive mood, (D) suicide ideation, and (E) suicide attempt.

The global effects of SHROs estimated by restricting the comparison group to never-treated units were similar to the findings of the main analyses, except that the signs of insignificant effect estimates for perceived stress in female students and suicide attempt in male students changed from negative to positive, and perceived stress and depressive mood in male students increased significantly as well (Supplementary Material 10). Findings from sensitivity analysis using asymptotic standard errors were also similar to those of the main analyses (Supplementary Material 11). TWFE models produced somewhat different results from the main CSDiD analyses, with significantly increased depressive mood and suicide attempts in total students and depressive mood and suicide ideation in female students, and inverted signs of insignificant effect estimates for perceived stress in total students, suicide attempts in male students, and sleep insufficiency in female students (Supplementary Material 12). Figure 4 and Supplementary Material 13 show the variation in the overall dynamic effects of SHROs by type and degree of potential violations of parallel trends. Even small relaxations of the parallel trends assumption increased uncertainty about the overall dynamic effect estimates, for which robust confidence intervals included zero for all mental health outcomes unless post-treatment trends were constrained to be perfectly linear.

## DISCUSSION

This study evaluated the effects of SHROs enforced at different times on the mental health of Korean middle and high school students using CSDiD models. No evidence was found that SHROs improved mental health among Korean adolescents, similar to previous findings that ratification of international human rights treaties, including the CRC, had no or mixed associations with improvements in population health [19-21,31,32]. Considering the overall effects aggregated into global, dynamic, group, and time effects and the results of sensitivity analyses using CSDiD and TWFE models, SHROs had no consistently significant effects on any mental health outcomes, except for slightly increased suicide ideation in total students (Table 2, Supplementary Materials 6-8 and 10-12). Moreover, pre-treatment parallel trends did not hold, except for suicide ideation in female students, and robust confidence intervals showed that uncertainty in the effect estimates of SHROs increased for all mental health outcomes with possible violations of parallel trends, rendering even originally significant effects insignificant (Figure 4, Supplementary Materials 5 and 13). It was also inconclusive whether SHROs insignificantly reduced suicide attempts in total and male students and perceived stress and sleep insufficiency in female students, contrary to the positive direction of the effects on other mental health outcomes, given the change in sign of the effect estimates from negative to positive in sensitivity analyses (Supplementary Materials 10, 12, and 13).

Unlike international economic treaties, international treaties on social issues such as human rights tend to impose costs on those regulated rather than immediate benefits, lack accountability mechanisms to promote compliance, and have relatively weak alliances among and financial support for human rights advocates [31,32]. Although the precise pathways through which human rights law becomes effective are unclear, it is noted that the only modifiable factor for improving the effectiveness of international treaties is enforcement mechanisms among accountability mechanisms for transparency (e.g., sharing information through regular reporting), oversight (e.g., active monitoring and assessment), complaint (e.g., allowing complaints to be processed and adjudicated), and enforcement (e.g., legal penalties and monetary fines) [20,32]. Additionally, domestic compliance with human rights standards requires legal infrastructural capacity (i.e., the capacity for legal rules to be enacted and enforced predictably and impartially) and government effectiveness (i.e., the capacity to implement quality public policies while responding to the needs of its citizens) [33]. Compared to other high-income countries, legal infrastructural capacity and government effectiveness in Korea were slightly lower from the mid-2000s to the mid-2010s, but have been higher after the mid-2010s, providing a potential foundation for domestic implementation of human rights standards [34]. However, SHROs with transparency, oversight, and complaint mechanisms are municipal ordinances, the lowest-level of legislation enacted to delegate or enforce the duties stipulated in higher-level laws, and therefore do not have enforcement mechanisms, such as penalty provisions, that could have contributed to achieving the intended effects of SHROs, possibly preventing them from improving adolescent mental health in Korea [35] (Supplementary Material 1).

The ineffectiveness of SHROs on adolescent mental health may not necessarily be evidence of their worthlessness. In a few studies in Korea using methods that are not robust to heterogeneous treatment effects in a staggered DiD design, SHROs have shown mixed associations with outcomes other than mental health: did not improve desirable behaviors (e.g., being responsible and orderly), peer/teacher relationships, and life satisfaction, but increased the likelihood of victimization due to problem behaviors (e.g., bullying and violence), and did not make school environments more human rights-promoting (e.g., students’ participation in making school rules), which might partly explain the results of this study that SHROs did not improve adolescent mental health; but reduced officially reported school violence among middle school students, insignificantly decreased compulsory night selfstudy time among high school students, and made school environments less human rights-violating (e.g., teacher’s corporal punishment) [36-39]. These findings implied that SHROs could have positive as well as null and negative effects on various outcomes, yet it was unclear what could explain the differences in effectiveness, suggesting the need for further research.

Since its first enactment, SHROs have been criticized for making it difficult to guide students by infringing on teachers’ professional authority due to increased students’ autonomy resulting from the expansion of students’ human rights [35,40]. Accordingly, the Chungnam and Seoul provincial councils abolished SHROs in April 2024, regardless of the evidence that the more students’ human rights are guaranteed, the more teachers’ professional authority is respected [40]. On the other hand, there are arguments for enacting a student human rights act because the absence of legal enforcement mechanisms for SHROs limits the protection of students’ human rights [35]. Such controversies over SHROs also call for further research to demonstrate the effect mechanisms for legal measures to ensure students’ human rights.

Some limitations should be noted. First, unobserved time-varying confounders may have differentially affected mental health outcomes in the treated or comparison group [24]. For example, private tutoring produces competition among students, which in turn reduces life satisfaction and increases depressive symptoms [13,41]. Compared to the comparison group, middle and high school students in the treated group, including the Seoul Capital Area, have participated in private tutoring more and spent more money, and the gap with the comparison group has widened since the mid-2010s [9]. Cooperation among students increases life satisfaction and positive affect (e.g., happiness) and reduces negative affect (e.g., sadness) without compromising academic performance, but the learning environment in the treated group became more competitive, which might have contributed to a null effect on the mental health of SHROs [9,41]. Future research is needed to evaluate the effects of human rights laws on mental health by accounting for the differential impact of social, economic, educational, and cultural time-varying confounders.

Second, due to the lack of panel data suitable for assessing the effects of SHROs on adolescent mental health, repeated cross-sectional data of KYRBS were used, which may lead to biased estimates of ATTs under compositional changes over time. However, the CSDiD model does not suffer from compositional changes by aggregating only ATTs for a fixed set of groups treated for at least some certain number of time periods [23]. Nonetheless, the use of repeated cross-sectional data rather than panel data widened the confidence intervals, increasing uncertainty in the effect estimates [26]. Third, all study variables, except for the intervention variable and area type, were measured by self-report, which may have introduced recall bias for most variables and social desirability bias for sensitive behaviors (e.g., alcohol use) and mental health outcomes. However, since it can be presumed that under-or over-reporting would have been systematic across provinces over time due to the use of nationally representative KYRBS data, those biases are unlikely to be associated with the effectiveness of SHROs. Additionally, it has been argued that for adolescents, self-reports provide more valid information than structured face-to-face interviews or reports from others on sensitive issues such as suicide behaviors [42]. Fourth, mental health outcomes were measured using single-item indicators, thus failing to capture complex aspects of mental health conditions. However, single-item indicators can be appropriate for measuring population mental health, given their significant associations with multi-item measures [43]. Although information on the validity of KYRBS data is limited, sensitivity and specificity were 92% and 93% for smoking and 69% and 100% for obesity, respectively [22]. The percent agreement ranged from 78% to 100% for the 39 health-risk behaviors, and reliability for suicide attempt was substantial, with percent agreement of 88% and kappa of 0.7 [22,44].

Despite these limitations and the ineffectiveness of SHROs, this study first estimated the effects of human rights legislation on adolescent mental health. The strengths of this study include that the effectiveness of SHROs was assessed using CSDiD models which are robust to treatment effect heterogeneity across provinces over time and that the variation in effect estimates by degree of potential violations of parallel trends was presented, providing insight into the interpretation of treatment effects in situations where pre-treatment parallel trends did not hold [23-25,27].

After the staggered implementation of SHROs in Korea, the mental health of middle and high school students in treated provinces did not improve compared to those in not-yet-treated and never-treated units. The ineffectiveness of SHROs might be due to the absence of enforcement mechanisms despite comprehensively guaranteeing human rights for students or the increased competitiveness of the learning environment in the treated group. Further empirical research is needed on the effectiveness of and effect mechanisms for legal measures to improve human rights on adolescent mental health. Policy support may also be required to create a less competitive and more cooperative learning environment for students.

## Figures and Tables

**Figure f5-epih-47-e2025011:**
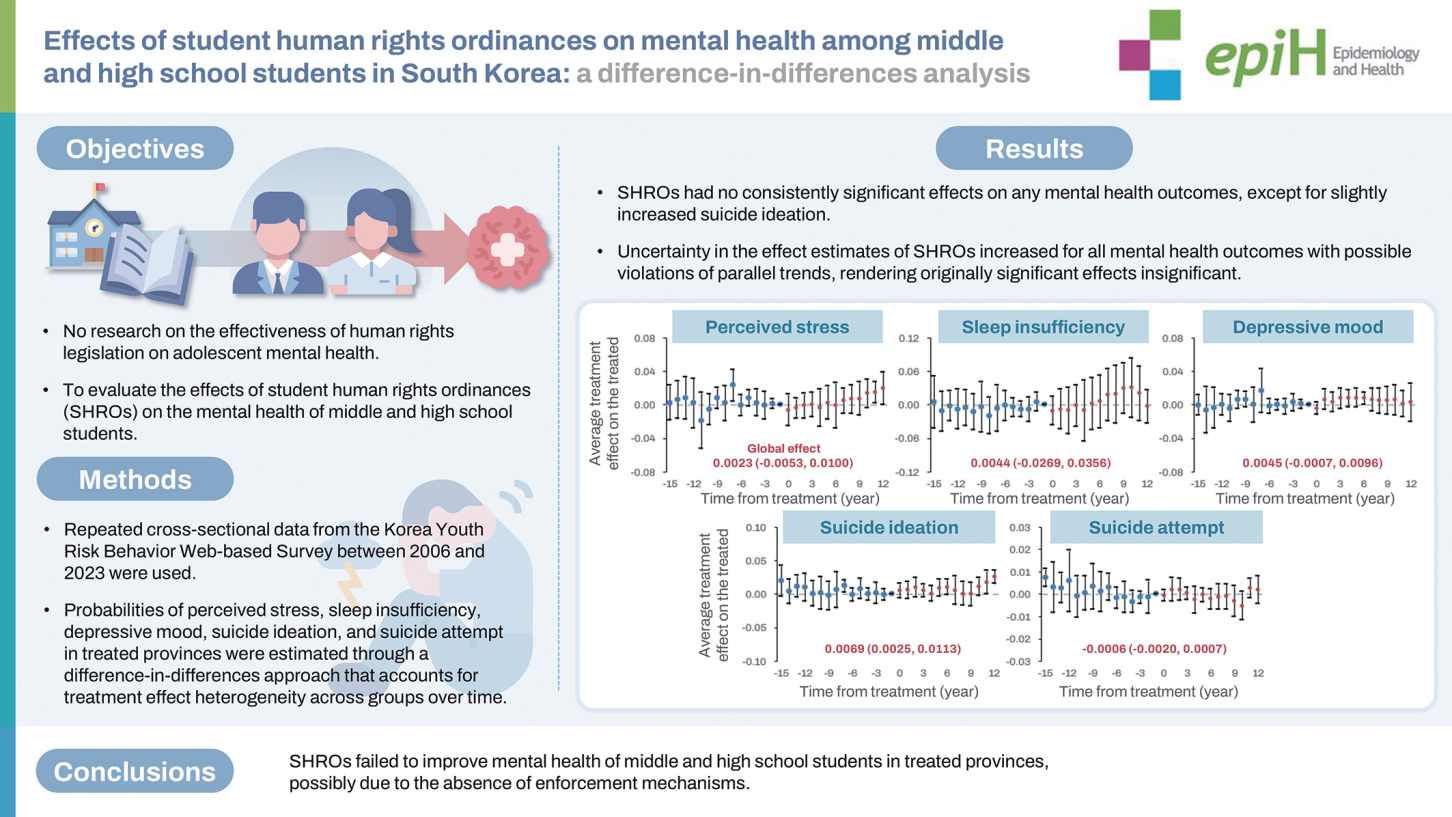


**Table 1.** Characteristics of treated and comparison groups during 2006-2023 in South Korea^1^

Variable	Category	Total		Treated group		Comparison group	
		N_unwt_	%_wt_	N_unwt_	%_wt_	N_unwt_	%_wt_
Total		1,148,257	100	628,459	100	519,798	100
Outcome							
Perceived stress	Yes	465,348	40.6	259,996	41.6	205,352	39.1
	No	682,909	59.4	368,463	58.4	314,446	60.9
Sleep insufficiency	Yes	465,648	40.9	253,662	40.7	211,986	41.2
	No	682,609	59.1	374,797	59.3	307,812	58.8
Depressive mood	Yes	356,569	31.2	197,679	32.0	158,890	30.1
	No	791,688	68.8	430,780	68.0	360,908	69.9
Suicide ideation	Yes	182,986	16.1	102,598	16.6	80,388	15.2
	No	965,271	83.9	525,861	83.4	439,410	84.8
Suicide attempt	Yes	40,509	3.5	22,442	3.6	18,067	3.3
	No	1,107,748	96.5	606,017	96.4	501,731	96.7
Potential covariate							
Sex	Male	591,788	52.4	321,557	52.0	270,231	53.0
	Female	556,469	47.6	306,902	48.0	249,567	47.0
Grade	7th	196,424	16.2	108,153	16.4	88,271	16.1
	8th	196,663	16.5	107,755	16.6	88,908	16.4
	9th	197,453	16.9	108,280	16.9	89,173	16.8
	10th	189,645	17.0	103,609	17.0	86,036	17.0
	11th	187,460	16.7	102,285	16.7	85,175	16.9
	12th	180,612	16.6	98,377	16.5	82,235	16.8
Age (yr)	12	82,470	6.8	45,772	6.9	36,698	6.7
	13	197,614	16.5	108,156	16.5	89,458	16.4
	14	199,128	16.9	109,304	16.9	89,824	16.8
	15	196,406	17.1	107,568	17.1	88,838	17.2
	16	190,411	17.0	103,892	17.0	86,519	17.1
	17	187,248	16.9	102,097	16.8	85,151	17.1
	18	94,980	8.8	51,670	8.8	43,310	8.8
Self-rated health	Good	1,062,883	92.5	580,603	92.4	482,280	92.8
	Poor	85,374	7.5	47,856	7.6	37,518	7.2
Perceived body image	Lean	735,476	64.3	402,538	64.5	332,938	64.1
	Fat	412,781	35.7	225,921	35.5	186,860	35.9
Eating breakfast (day/wk)	0	176,792	15.3	100,012	15.7	76,780	14.7
	1-2	164,321	14.2	91,968	14.5	72,353	13.7
	3-5	264,428	22.8	147,085	23.1	117,343	22.4
	6-7	542,716	47.7	289,394	46.8	253,322	49.2
Eating fast food (frequency/wk)	0	325,187	28.1	172,933	27.7	152,254	28.8
	1-2	602,697	52.5	331,399	52.3	271,298	52.7
	≥3	220,373	19.4	124,127	19.9	96,246	18.5
Vigorous physical activity (day/wk)	0	327,495	28.9	178,805	28.9	148,690	28.8
	1-2	424,438	37.0	231,117	36.7	193,321	37.5
	≥3	396,324	34.1	218,537	34.3	177,787	33.8
Muscle-strengthening activity (day/wk)	0	561,168	49.1	312,150	49.8	249,018	48.0
	1-2	337,668	29.2	180,477	28.6	157,191	30.2
	≥3	249,421	21.7	135,832	21.5	113,589	21.9
Body mass index	Normal or under-weight	950,268	83.1	521,326	83.5	428,942	82.6
	Overweight or obese	197,989	16.9	107,133	16.5	90,856	17.4
Alcohol use	Yes	524,205	46.0	279,318	45.5	244,887	46.8
	No	624,052	54.0	349,141	54.5	274,911	53.2
Cigarette or nicotine use	Yes	140,828	12.6	72,295	12.5	68,533	12.8
	No	1,007,429	87.4	556,164	87.5	451,265	87.2
Academic performance	High	424,269	37.0	232,023	36.9	192,246	37.2
	Middle	324,878	28.3	178,084	28.3	146,794	28.2
	Low	399,110	34.7	218,352	34.8	180,758	34.6
Family socioeconomic status	High	387,038	34.5	218,330	35.4	168,708	33.2
	Middle	546,467	47.3	294,558	46.5	251,909	48.4
	Low	214,752	18.2	115,571	18.1	99,181	18.5
Living arrangement	With family	1,094,746	96.1	603,836	96.7	490,910	95.1
	With non-family	53,511	3.9	24,623	3.3	28,888	4.9
Area type	Metropolitan	730,289	69.2	492,487	84.8	237,802	44.9
	Non-metropolitan	417,968	30.8	135,972	15.2	281,996	55.1

N_unwt_, unweighted frequency; %_wt_, weighted percentage.

1 All variables except area type were measured by self-report.

**Table 2.** Global effects of student human rights ordinances on mental health among middle and high school students in South Korea^1^

Outcome	Total	Male	Female
	Average treatment effect on the treated (95% CI)	Average treatment effect on the treated (95% CI)	Average treatment effect on the treated (95% CI)
Perceived stress	0.0023 (-0.0053, 0.0100)	0.0083 (-0.0000, 0.0167)	-0.0014 (-0.0124, 0.0096)
Sleep insufficiency	0.0044 (-0.0269, 0.0356)	0.0078 (-0.0290, 0.0446)	-0.0021 (-0.0343, 0.0301)
Depressive mood	0.0045 (-0.0007, 0.0096)	0.0068 (-0.0002, 0.0138)	0.0081 (-0.0019, 0.0182)
Suicide ideation	0.0069 (0.0025, 0.0113)	0.0077 (0.0031, 0.0124)	0.0058 (-0.0009, 0.0126)
Suicide attempt	-0.0006 (-0.0020, 0.0007)	-0.0008 (-0.0054, 0.0038)	0.0006 (-0.0018, 0.0029)

CI, confidence interval.

1 The estimated effects represent weighted averages of the overall group-time average treatment effects, with weights being proportional to the size of each group.

## Supplementary materials

Supplementary Material 1. Main contents of student human rights ordinances

Supplementary Material 2. Description of the study variables

Supplementary Material 3. Difference-in-differences model specifications

Supplementary Material 4. Characteristics of treated and comparison groups by sex during 2006-2023 in South Korea

Supplementary Material 5. Results of pre-treatment parallel trends analysis

Supplementary Material 6. Dynamic average treatment effects of student human rights ordinances on mental health among middle and high school students in South Korea

Supplementary Material 7. Group average treatment effects of student human rights ordinances on mental health among middle and high school students in South Korea

Supplementary Material 8. Time average treatment effects of student human rights ordinances on mental health among middle and high school students in South Korea

Supplementary Material 9. Group-time average treatment effects of student human rights ordinances on mental health among middle and high school students in South Korea

Supplementary Material 10. Global effects of student human rights ordinances on mental health among middle and high school students in South Korea estimated using never-treated units as a comparison group

Supplementary Material 11. Global effects of student human rights ordinances on mental health among middle and high school students in South Korea estimated without using the wild cluster bootstrap

Supplementary Material 12. Effects of student human rights ordinances on mental health among middle and high school students in South Korea estimated by two-way fixed effects regression

Supplementary Material 13. Robust confidence intervals for the overall dynamic effects of student human rights ordinances by type and degree of potential violations of parallel trends
