# Supplementary material for: Effects of student human rights ordinances on mental health among middle and high school students in South Korea: a difference-in-differences analysis
Source: Epidemiol Health. 2025 Mar 1;47:e2025011. doi: 10.4178/epih.e2025011 (PMC12062860; doi:10.4178/epih.e2025011)
Supplement: Supplementary Material 1. — Main contents of student human rights ordinances [file epih-47-e2025011-Supplementary-1.docx]

Supplementary Material 1. Main contents of student human rights ordinances

| Substantive and procedural guarantees in student human rights ordinance and examples of articles of the Seoul's ordinance | Relevant articles of student human rights ordinance for each province | | | | | | | Relevant articles of the Conven­tion on the Rights of the Child |
| --- | --- | --- | --- | --- | --- | --- | --- | --- |
|  | Gyeonggi (enacted and enforced on October 5, 2010) | Gwangju (enacted on October 28, 2011 and enforced on January 1, 2012) | Seoul (enacted and enforced on January 26, 2012) | Jeonbuk (enacted and enforced on July 12, 2013) | Chung­nam (enacted and enforced on July 10, 2020) | Jeju (enacted and enforced on January 8, 2021) | Incheon (enacted on April 12, 2021 and enforced on Septem­ber 1, 2021) |  |
| • Right not to be discriminated Students have a right not to be discriminated on grounds of gender, religion, age, social status, place of birth, country of origin, ethnicity, language, disability, physical conditions such as appearance, pregnancy or childbirth, family type or situation, race, economic status, skin color, thought or political opinion, sexual orientation, gender identity, medical history, disciplinary actions, and/or grades. | 5 | 20 | 5 | 8 | 15 | 8 | 5 | 2 |
| • Right to be free from violence Students have a right to be free from all physical and verbal violence, including, but not limited to, corporal punishment, bullying, bullying in group and sexual violence. The Superintendent, the heads of schools and the school personnel shall prevent all physical and verbal violence, including, but not limited to, corporal punishment, group-bullying and sexual violence. | 6 | 19 | 6 | 9 | 13 | 9 | 6, 13 | 19, 28, 34 |
| • Right to safety The Superintendent, the founders and the managers of schools, the heads of schools, and the school personnel shall organize and maintain a safety management system to ensure students' safety. | 7 | 19 | 7 | 10 | 26 | 10 | 21 | 24, 27, 28 |
| • Right to learn Students have a right to learn what is appropriate to their talent, aptitude and environment. | 8 | 10 | 8 | 5 | 23 | 5 | 16 | 28, 29 |
| • Right to educational activities other than regular school curriculum Students have a right to choose freely educational activities other than regular school curriculum, such as prep study and after school programs. Schools shall not run curriculum arbitrarily or force students to participate in non-mandatory events held in and outside of schools. | 9 | 10 | 9 | 6 | 18 | 6 | 16 | 28, 29 |
| • Right to rest and leisure Students have a right to escape from burden of study and get adequate rest and leisure to form and develop a healthy and distinctive ego. The heads of schools and the school personnel shall not force students to participate in educational activities other than regular school curriculum against their will and thereby violating their right to rest and leisure. | 10 | 18 | 10 | 11 | 27 | 7 | 23 | 31 |
| • Right to cultural activity Students have a right to enjoy a variety of cultural activities. | 22 | 18 | 11 | 23 | 27 | 22 | 23 | 31 |
| • Right to individuality Students have a right to express their individuality through their appearance, such as clothes and hair. | 11 | 14 | 12 | 12 | 9 | 11 | 9 | 12 |
| • Freedom of personal life Students have a right to have the freedom and privacy of their personal lives, such as their belongings, private records, private space, and private relationships, not violated or under surveillance. | 12 | 12 | 13 | 13 | 10 | 12 | 10 | 16 |
| • Right to personal information Students have a right to protection of their personal information, related to family, friendship, grade, medical history, records of disciplinary actions, any unpaid educational expenses, counseling records, sexual orientation, etc. | 13 | 12 | 14 | 14 | 10 | 12 | 10 | 16 |
| • Right to access to the personal information Students or guardians have a right to access to their own personal information, such as the school record for themselves or the concerned students, and have a right to request its correction, its deletion, or cessation of its processing. | 14 | 12 | 15 | 15 | 11, 12 | 14 | 11 | 17 |
| • Freedom of conscience and religion Students have a right to freedom of conscience and religion, such as their view of the world, their view of life, or value and ethical judgment. The founders and the managers of schools, the heads of schools and the school personnel shall not force students to make a statement, such as regretting their wrongdoings or taking an oath, against their conscience. | 15 | 13 | 16 | 16 | 7 | 15 | 7 | 14 |
| • Freedom of expression Students have a right to freely express their ideas through various means and to be respected of such ideas. Students have a right to gather the opinions of school community members through collecting signatures for petition or conducting surveys. Students have a right to assembly: Provided, that school rules may restrict the time, place and means of an assembly taking place within school boundaries to the minimum extent necessary to protect students' right to learn and safety. | 16 | 14 | 17 | 17 | 8 | 16 | 8 | 13 |
| • Right to self-governing activities Students have a right to engage in self-governing activities, such as establishing, convening, running and participating in student clubs, student council and other student self-governing organizations. The heads of schools and the school personnel shall not restrict the membership eligibility of student self-governing organizations for such reasons as grade and records of disciplinary actions. | 17 | 15 | 18 | 18 | 19 | 17 | - | 15 |
| • Right to participate in making school rules, such as school disciplines Students have a right to participate in the enactment and amendment of school rules, such as school disciplines. | 18 | 15 | 19 | 19 | 20 | 18 | 17 | 3, 12, 40, 41 |
| • Right to participate in policy decision Students have a right to participate in school operations and in the process of making education policy decisions by the provincial Office of Education. Voluntary associations of student self-governing organizations, including, but not limited to, student councils, shall have a right to submit the opinions on matters related to students' rights. | 19 | 15 | 20 | 20 | 21 | 19 | 17 | 3, 12, 40, 41 |
| • Right to school welfare Students have a right to receive proper support from schools, such as counseling, in order to overcome crises of every kind, including but not limited to learning difficulties, violence victimization, crisis at home and delinquency, and to develop their identity through discovering their aptitude and looking into future career paths. | 20 | 17 | 21 | 21 | 23 | 20 | 20 | 20-23, 26, 39 |
| • Right to educational environment Students have a right to receive education in a healthy and pleasant environment. | 21 | 19 | 22 | 22 | 24 | 21 | 21 | 24, 28 |
| • Right to school meals Students have a right to be provided with school meals prepared with safe ingredients. | 23 | 19 | 23 | 24 | 25 | 23 | 22 | 24, 27 |
| • Right to health Students have a right to maintain optimal physical condition, receive proper treatment when they are sick, and use healthcare facilities conveniently. | 24 | 19 | 24 | 25 | 24 | 24 | 21 | 24 |
| • Procedural rights in disciplinary measures The disciplinary measures against students shall be taken according to justifiable rules that meet the human rights standards and in due process, such as providing prior notice of the reason of such disciplinary measures, forming an impartial review board, ensuring an opportunity to defense, assuring the right to appoint defending counsel, and ensuring the right to demand renewal of procedure. | 25 | 16 | 25 | 26 | 14 | 25 | 14 | 37, 40 |
| • Right to request counseling and investigation Everyone, including students, has a right to request counseling and investigation from the Advocate Officer for Students' Human Rights ("Advocate") when students' human rights are violated. | 26 | 22 | 27 | 27 | 24 | 26 | 20 | 39 |
| • Ensuring rights of minority students The Superintendent, founders and managers of schools, heads of schools and school personnel shall ensure the rights of minority students, including students from low-income families, students with disabilities, students from single-parent families, students from multicultural families, foreign students, student athletes, sexual minority students, and working students ("minority students"), which are required due to their characteristics, to a reasonable level. | 20 | 21 | 28 | 38 | 28 | 8 | 20 | 20-23, 30 |
| • Students' human rights education The Superintendent, the founders and the managers of schools, the heads of schools and the school personnel shall provide education on students' human rights that is required to increase and improve everyone's awareness on students' human rights. The heads of schools shall offer education on students' human rights to the students for two hours or more per semester. When offering the education, heads of schools shall include content related to the right to work in the education, taking into consideration the students of high schools tailored to industrial demand and specialized vocational high schools and an increasing number of working students. | 30 | 35 | 29 | 30 | 44 | 29 | 31 | 42 |
| • Human rights education for school personnel The heads of schools shall provide the school personnel with education on students' human rights for two hours or more per year. | 31 | 36 | 31 | 31 | 46 | 30 | 32 | 42 |
| • Human rights education for guardians The heads of schools shall offer education on students' human rights or hold a meeting on the subject for guardians once or more per year. | 32 | 37 | 31 | 32 | 46 | 31 | 33 | 42 |
| • The Students' Human Rights Commission The Students' Human Rights Commission ("Commission") shall be established in order to deliberate on important policies with which the Office of Education may promote students' human rights and create human rights friendly education culture, and on solutions on human rights violation cases at the sites of education, and to form a public opinion of and collaborate with the local community on the topic of students' human rights. | 35 | 23 | 33 | 40 | 29 | 35 | 24 | 43 |
| • Participatory Organization of Students The Superintendent shall establish a Participatory Organization of Students ("Organization") to collect the opinions of students in the policy making processes to promote students' human rights and create an educational environment that is human rights friendly. | 36 | 29 | 37 | 41 | 35 | Enforce­ment Rule 6 | - | 3, 12 |
| • Advocate Officer for Students' Human Rights The Superintendent shall have one Advocate at the Office of Education to promote students' human rights and create human rights friendly education culture. | 39 | - | 38 | 43 | 32 | - | 27 | 43 |
| • Students' human rights education center The Office of Education shall have a students' human rights education center ("Center") led by the Advocate to ensure effective job performance of the Advocate. | - | 38 | 42 | 42, 46 | 34 | 36 | - | 43 |
| • Students' human rights impact evaluation The Commission may evaluate in advance and present views on the impact of the ordinance or policy to be enacted or drawn up by the Superintendent on students' human rights and human rights friendly education culture. In case the ordinance or policy the Superintendent promotes is deemed to be against the guarantee of students' human rights, the Commission may recommend the improvement or suspension thereof. | - | 27 | 43 | - | 38 | - | - | 3 |
| • Establishment of the action plan on students' human rights The Superintendent shall establish and implement an action plan to substantially promote human rights friendly educational culture every three years after the deliberation of the Commission. | 34 | 4 | 44 | 35 | 36 | 34 | 26 | 43 |
| • Annual implementation plan and factual Survey If students experience violation of their human rights or face a risk thereof, anyone, including students, may make a request for remedy to the Advocate. Each District Office of Education shall have a students' human rights counseling center to handle requests for remedy in an efficient manner. The Superintendent shall provide each school with detailed guidelines, if necessary, to promote students' human rights, and guide and supervise the heads of schools who do not follow the guidelines without valid reasons. | 37, 43 | 5 | 45 | 36, 48 | 37 | 33 | - | 44 |
| • Request for remedy for violation of students' human rights If students experience violation of their human rights or face a risk thereof, anyone, including students, may make a request for remedy to the Advocate. | 44 | 40 | 47 | 49 | 39 | 37 | 28 | 39 |
| • Investigation of students' human rights violation cases The Advocate shall investigate the case with the consent of the person who argues that his/her human rights have been violated. The Advocate may ask the Office of Education and schools for materials when necessary for the investigation, and ask questions to the founder and the manager of school, the head of school, the school personnel, guardians, students and public officials concerned. | 45 | 41 | 48 | 50 | 40 | 38 | 29 | 39 |
