# Supplementary material for: Effects of student human rights ordinances on mental health among middle and high school students in South Korea: a difference-in-differences analysis
Source: Epidemiol Health. 2025 Mar 1;47:e2025011. doi: 10.4178/epih.e2025011 (PMC12062860; doi:10.4178/epih.e2025011)
Supplement: Supplementary Material 2. — Description of the study variables [file epih-47-e2025011-Supplementary-2.docx]

Supplementary Material 2. Description of the study variables

| Variable | | Description | Code |
| --- | --- | --- | --- |
| Intervention | | The year when a province first enforced student human rights ordinance (= the earliest year when the Korea Youth Risk Behavior Web-based Survey was conducted following the first enforcement of student human rights ordinance) | 0: Busan, Daegu, Daejeon, Ulsan, Gangwon, Chungbuk, Jeonnam, Gyeongbuk, Gyeongnam (never treated units) 2011: Gyeonggi 2012: Seoul, Gwangju 2014: Jeonbuk 2020: Chungnam 2021: Incheon, Jeju |
| Outcome | | | |
|  | Perceived stress | "How much stress do you feel in your daily life?" | 0: a little, not much, not at all (no) 1: very much, a lot (yes) |
|  | Sleep insufficiency | "During the last 7 days, do you think the amount of time you slept was enough to recover from fatigue?" | 0: more or less, enough, very enough (no) 1: very not enough, not enough (yes) |
|  | Depressive mood | "During the last 12 months, have you ever felt so sad or hopeless enough to stop your daily life over the two weeks?" | 0: no 1: yes |
|  | Suicide ideation | "During the last 12 months, have you ever seriously considered suicide?" | 0: no 1: yes |
|  | Suicide attempt | "During the last 12 months, have you attempted suicide?" | 0: no 1: yes |
| Potential covariate | | | |
|  | Sex | "What is your sex" | 0: male 1: female |
|  | Grade | "What grade are you in?" | 7th, 8th, 9th, 10th, 11th, and 12th grade |
|  | Age | Calculated from responses to the survey question “What year and month were you born?” | 12, 13, 14, 15, 16, 17, and 18 years old |
|  | Self-rated health | "How healthy do you usually feel?" | 0: average, healthy, very healthy (good) 1: very not healthy, not healthy (poor) |
|  | Perceived body image | "What do you think about your body image?" | 0: average, lean, very lean (lean) 1: very fat, fat (fat) |
|  | Eating breakfast | "During the last 7 days, on how many days did you eat breakfast, excluding just milk or juice?" | 0: none 1: 1-2 days/week 2: 3-5 days/week 3: 6-7 days/week |
|  | Eating fast food | "During the last 7 days, how often did you eat fast food?" | 0: none 1: 1-2 times/week 2: ≥3 times/week |
|  | Vigorous physical activity | "During the last 7 days, on how many days did you do any type of vigorous physical activity that made you out of breath or sweaty for more than 20 minutes?" | 0: none 1: 1-2 days/week 2: ≥3 days/week |
|  | Muscle-strengthening activity | "During the past 7 days, on how many days did you do any type of physical activity that builds muscle strength (muscle-strengthening activity)?" | 0: none 1: 1-2 days/week 2: ≥3 days/week |
|  | Body mass index | After calculating body mass index from responses to the survey question “What is your height and weight?”, overweight and obesity were classified according to the 2017 Korean National Growth Charts. | 0: normal, underweight 1: obesity, overweight |
|  | Alcohol use | "Have you ever drunk more than a sip of alcohol?" | 0: no 1: yes |
|  | Cigarette or nicotine use | "Have you ever smoked more than a puff of a cigarette?", “Have you ever taken a puff of e-cigarette containing nicotine?”, or "Have you ever used heated tobacco products?" | 0: no to all questions 1: yes to any question |
|  | Academic performance | "During the last 12 months, how was your academic performance?" | 0: high 1: middle 2: low |
|  | Family socioeconomic status | "How was your family's financial situation?" | 0: high 1: middle 2: low |
|  | Living arrangement | "What is your current living arrangement?" | 0: living with family 1: living with non-family |
|  | Area type | Seoul, Busan, Daegu, Incheon, Gwangju, Daejeon, Ulsan, and Gyeonggi were classified as metropolitan areas, and Gangwon, Chungbuk, Chungnam, Jeonbuk, Jeonnam, Gyeongbuk, Gyeongnam, and Jeju were classified as non-metropolitan areas. | 0: non-metropolitan areas 1: metropolitan areas |
|  | Outcome variables not used as a regressand |  |  |

Note: All variables except for the intervention variable and area type were measured by self-report.
