# Supplementary material for: Effects of student human rights ordinances on mental health among middle and high school students in South Korea: a difference-in-differences analysis
Source: Epidemiol Health. 2025 Mar 1;47:e2025011. doi: 10.4178/epih.e2025011 (PMC12062860; doi:10.4178/epih.e2025011)
Supplement: Supplementary Material 3. — Difference-in-differences model specifications [file epih-47-e2025011-Supplementary-3.docx]

Supplementary Material 3. Difference-in-differences model specifications

| Outcome | | Covariate | | |
| --- | --- | --- | --- | --- |
|  |  | Total | Male | Female |
|  | Perceived stress | Sex, self-rated health, perceived body image, muscle-strengthening activity, body mass index | Area type | Grade, perceived body image, family socioeconomic status, area type |
|  | Sleep insufficiency | Grade, perceived body image, eating fast food, muscle-strengthening activity, living arrangement | Cigarette or nicotine use | Self-rated health, perceived body image, muscle-strengthening activity, alcohol use, family socioeconomic status, living arrangement, suicide ideation |
|  | Depressive mood | Muscle-strengthening activity, suicide ideation | Grade, body mass index, area type | Academic performance, area type, suicide ideation |
|  | Suicide ideation | Grade | Cigarette or nicotine use | Perceived body image, muscle-strengthening activity, sleep insufficiency |
|  | Suicide attempt | Perceived body image, eating fast food, vigorous physical activity, cigarette or nicotine use, area type | Perceived body image, alcohol use, perceived stress | Muscle-strengthening activity, family socioeconomic status, living arrangement |
