# Supplementary material for: Effects of student human rights ordinances on mental health among middle and high school students in South Korea: a difference-in-differences analysis
Source: Epidemiol Health. 2025 Mar 1;47:e2025011. doi: 10.4178/epih.e2025011 (PMC12062860; doi:10.4178/epih.e2025011)
Supplement: Supplementary Material 4. — Characteristics of treated and comparison groups by sex during 2006-2023 in South Korea [file epih-47-e2025011-Supplementary-4.docx]

Supplementary Material 4. Characteristics of treated and comparison groups by sex during 2006-2023 in South Korea

| Variable | Category | Male | | | | | | Female | | | | | | |
| --- | --- | --- | --- | --- | --- | --- | --- | --- | --- | --- | --- | --- | --- | --- |
|  |  | Total | | Treated group | | Comparison group | | Total | | Treated group | | Comparison group | | |
|  |  | Un­weighted frequency | Weight­ed % | Un­weighted frequency | Weight­ed % | Un­weighted frequency | Weight­ed % | Un­weighted frequency | Weight­ed % | Un­weighted frequency | Weight­ed % | Un­weighted frequency | Weight­ed % |  |
| Total |  | 591,788 | 100.0 | 321,557 | 100.0 | 270,231 | 100.0 | 556,469 | 100.0 | 306,902 | 100.0 | 249,567 | 100.0 |  |
| Outcome | | | | | | | | | | | | | | |
| Perceived stress | Yes | 200,300 | 34.2 | 111,073 | 35.0 | 89,227 | 32.9 | 265,048 | 47.6 | 148,923 | 48.6 | 116,125 | 46.0 |  |
|  | No | 391,488 | 65.8 | 210,484 | 65.0 | 181,004 | 67.1 | 291,421 | 52.4 | 157,979 | 51.4 | 133,442 | 54.0 |  |
| Sleep insufficiency | Yes | 207,548 | 35.6 | 111,512 | 35.2 | 96,036 | 36.1 | 258,100 | 46.8 | 142,150 | 46.6 | 115,950 | 47.0 |  |
|  | No | 384,240 | 64.4 | 210,045 | 64.8 | 174,195 | 63.9 | 298,369 | 53.2 | 164,752 | 53.4 | 133,617 | 53.0 |  |
| Depressive mood | Yes | 153,702 | 26.4 | 84,645 | 27.1 | 69,057 | 25.4 | 202,867 | 36.5 | 113,034 | 37.3 | 89,833 | 35.4 |  |
|  | No | 438,086 | 73.6 | 236,912 | 72.9 | 201,174 | 74.6 | 353,602 | 63.5 | 193,868 | 62.7 | 159,734 | 64.6 |  |
| Suicide ideation | Yes | 73,258 | 12.6 | 40,658 | 13.0 | 32,600 | 12.0 | 109,728 | 19.9 | 61,940 | 20.5 | 47,788 | 18.8 |  |
|  | No | 518,530 | 87.4 | 280,899 | 87.0 | 237,631 | 88.0 | 446,741 | 80.1 | 244,962 | 79.5 | 201,779 | 81.2 |  |
| Suicide attempt | Yes | 15,214 | 2.6 | 8,238 | 2.6 | 6,976 | 2.5 | 25,295 | 4.5 | 14,204 | 4.7 | 11,091 | 4.3 |  |
|  | No | 576,574 | 97.4 | 313,319 | 97.4 | 263,255 | 97.5 | 531,174 | 95.5 | 292,698 | 95.3 | 238,476 | 95.7 |  |
| Potential covariate | | | | | | | | | | | | | | |
| Grade | 7th | 102,740 | 16.2 | 55,849 | 16.4 | 46,891 | 16.0 | 93,684 | 16.3 | 52,304 | 16.3 | 41,380 | 16.2 |  |
|  | 8th | 101,988 | 16.5 | 55,438 | 16.6 | 46,550 | 16.4 | 94,675 | 16.6 | 52,317 | 16.6 | 42,358 | 16.5 |  |
|  | 9th | 102,450 | 16.9 | 55,764 | 16.9 | 46,686 | 16.8 | 95,003 | 16.9 | 52,516 | 16.9 | 42,487 | 16.9 |  |
|  | 10th | 98,202 | 17.0 | 53,249 | 17.0 | 44,953 | 17.1 | 91,443 | 16.9 | 50,360 | 16.9 | 41,083 | 16.9 |  |
|  | 11th | 95,126 | 16.8 | 51,840 | 16.7 | 43,286 | 16.9 | 92,334 | 16.7 | 50,445 | 16.7 | 41,889 | 16.8 |  |
|  | 12th | 91,282 | 16.6 | 49,417 | 16.5 | 41,865 | 16.9 | 89,330 | 16.6 | 48,960 | 16.6 | 40,370 | 16.7 |  |
| Age (years) | 12 | 42,515 | 6.7 | 23,321 | 6.8 | 19,194 | 6.6 | 39,955 | 6.9 | 22,451 | 7.0 | 17,504 | 6.8 |  |
|  | 13 | 103,031 | 16.4 | 55,736 | 16.5 | 47,295 | 16.3 | 94,583 | 16.5 | 52,420 | 16.5 | 42,163 | 16.5 |  |
|  | 14 | 103,324 | 16.9 | 56,356 | 17.0 | 46,968 | 16.7 | 95,804 | 16.9 | 52,948 | 16.9 | 42,856 | 16.9 |  |
|  | 15 | 101,592 | 17.1 | 55,143 | 17.1 | 46,449 | 17.1 | 94,814 | 17.2 | 52,425 | 17.2 | 42,389 | 17.2 |  |
|  | 16 | 97,605 | 17.0 | 52,907 | 17.0 | 44,698 | 17.2 | 92,806 | 17.0 | 50,985 | 17.0 | 41,821 | 17.0 |  |
|  | 17 | 94,620 | 16.9 | 51,496 | 16.7 | 43,124 | 17.1 | 92,628 | 17.0 | 50,601 | 16.9 | 42,027 | 17.1 |  |
|  | 18 | 49,101 | 9.0 | 26,598 | 8.9 | 22,503 | 9.1 | 45,879 | 8.6 | 25,072 | 8.6 | 20,807 | 8.6 |  |
| Self-rated health | Good | 555,430 | 93.8 | 301,485 | 93.7 | 253,945 | 93.9 | 507,453 | 91.1 | 279,118 | 90.9 | 228,335 | 91.5 |  |
|  | Poor | 36,358 | 6.2 | 20,072 | 6.3 | 16,286 | 6.1 | 49,016 | 8.9 | 27,784 | 9.1 | 21,232 | 8.5 |  |
| Perceived body image | Lean | 397,458 | 67.3 | 216,200 | 67.5 | 181,258 | 67.0 | 338,018 | 61.1 | 186,338 | 61.2 | 151,680 | 60.8 |  |
|  | Fat | 194,330 | 32.7 | 105,357 | 32.5 | 88,973 | 33.0 | 218,451 | 38.9 | 120,564 | 38.8 | 97,887 | 39.2 |  |
| Eating breakfast (days/week) | 0 | 92,967 | 15.7 | 52,318 | 16.0 | 40,649 | 15.1 | 83,825 | 14.9 | 47,694 | 15.2 | 36,131 | 14.4 |  |
|  | 1-2 | 78,490 | 13.2 | 43,584 | 13.5 | 34,906 | 12.8 | 85,831 | 15.3 | 48,384 | 15.6 | 37,447 | 14.7 |  |
|  | 3-5 | 127,214 | 21.4 | 70,442 | 21.6 | 56,772 | 21.0 | 137,214 | 24.4 | 76,643 | 24.6 | 60,571 | 24.0 |  |
|  | 6-7 | 293,117 | 49.7 | 155,213 | 48.8 | 137,904 | 51.2 | 249,599 | 45.4 | 134,181 | 44.5 | 115,418 | 46.9 |  |
| Eating fast food (frequency/week) | 0 | 164,995 | 27.7 | 86,513 | 27.1 | 78,482 | 28.5 | 160,192 | 28.7 | 86,420 | 28.4 | 73,772 | 29.1 |  |
|  | 1-2 | 304,955 | 51.6 | 166,863 | 51.5 | 138,092 | 51.7 | 297,742 | 53.5 | 164,536 | 53.2 | 133,206 | 53.9 |  |
|  | ≥3 | 121,838 | 20.7 | 68,181 | 21.3 | 53,657 | 19.8 | 98,535 | 17.9 | 55,946 | 18.4 | 42,589 | 16.9 |  |
| Vigorous physical activity (days/week) | 0 | 100,895 | 17.4 | 55,006 | 17.4 | 45,889 | 17.4 | 226,600 | 41.5 | 123,799 | 41.4 | 102,801 | 41.6 |  |
|  | 1-2 | 214,005 | 36.5 | 114,427 | 35.9 | 99,578 | 37.3 | 210,433 | 37.6 | 116,690 | 37.7 | 93,743 | 37.6 |  |
|  | ≥3 | 276,888 | 46.2 | 152,124 | 46.7 | 124,764 | 45.3 | 119,436 | 20.9 | 66,413 | 20.9 | 53,023 | 20.8 |  |
| Muscle-strengthening activity (days/week) | 0 | 201,833 | 34.5 | 111,941 | 35.2 | 89,892 | 33.6 | 359,335 | 65.1 | 200,209 | 65.7 | 159,126 | 64.2 |  |
|  | 1-2 | 197,789 | 33.2 | 105,076 | 32.6 | 92,713 | 34.2 | 139,879 | 24.8 | 75,401 | 24.3 | 64,478 | 25.6 |  |
|  | ≥3 | 192,166 | 32.2 | 104,540 | 32.2 | 87,626 | 32.2 | 57,255 | 10.1 | 31,292 | 10.0 | 25,963 | 10.2 |  |
| Body mass index | Normal or under­weight | 473,944 | 80.4 | 257,984 | 80.8 | 215,960 | 79.9 | 476,324 | 86.1 | 263,342 | 86.5 | 212,982 | 85.6 |  |
|  | Over­weight or obese | 117,844 | 19.6 | 63,573 | 19.2 | 54,271 | 20.1 | 80,145 | 13.9 | 43,560 | 13.5 | 36,585 | 14.4 |  |
| Alcohol use | Yes | 289,459 | 49.5 | 154,110 | 49.0 | 135,349 | 50.3 | 234,746 | 42.1 | 125,208 | 41.6 | 109,538 | 42.8 |  |
|  | No | 302,329 | 50.5 | 167,447 | 51.0 | 134,882 | 49.7 | 321,723 | 57.9 | 181,694 | 58.4 | 140,029 | 57.2 |  |
| Cigarette or nicotine use | Yes | 97,525 | 16.8 | 50,029 | 16.5 | 47,496 | 17.3 | 43,303 | 8.0 | 22,266 | 8.1 | 21,037 | 7.7 |  |
|  | No | 494,263 | 83.2 | 271,528 | 83.5 | 222,735 | 82.7 | 513,166 | 92.0 | 284,636 | 91.9 | 228,530 | 92.3 |  |
| Academic performance | High | 222,699 | 37.6 | 120,386 | 37.4 | 102,313 | 38.1 | 201,570 | 36.2 | 111,637 | 36.3 | 89,933 | 36.1 |  |
|  | Middle | 163,443 | 27.6 | 89,042 | 27.7 | 74,401 | 27.5 | 161,435 | 29.0 | 89,042 | 29.0 | 72,393 | 29.0 |  |
|  | Low | 205,646 | 34.7 | 112,129 | 35.0 | 93,517 | 34.4 | 193,464 | 34.8 | 106,223 | 34.7 | 87,241 | 34.9 |  |
| Family socioeco­nomic status | High | 215,506 | 37.0 | 120,317 | 37.9 | 95,189 | 35.6 | 171,532 | 31.8 | 98,013 | 32.6 | 73,519 | 30.4 |  |
|  | Middle | 268,180 | 45.0 | 143,421 | 44.3 | 124,759 | 46.0 | 278,287 | 49.7 | 151,137 | 49.0 | 127,150 | 51.0 |  |
|  | Low | 108,102 | 18.0 | 57,819 | 17.8 | 50,283 | 18.3 | 106,650 | 18.5 | 57,752 | 18.4 | 48,898 | 18.6 |  |
| Living arrangement | Living with family | 562,259 | 95.7 | 307,755 | 96.4 | 254,504 | 94.6 | 532,487 | 96.5 | 296,081 | 97.1 | 236,406 | 95.6 |  |
|  | Living with non-family | 29,529 | 4.3 | 13,802 | 3.6 | 15,727 | 5.4 | 23,982 | 3.5 | 10,821 | 2.9 | 13,161 | 4.4 |  |
| Area type | Metropoli­tan | 375,311 | 69.0 | 249,660 | 84.7 | 125,651 | 45.0 | 354,978 | 69.4 | 242,827 | 84.9 | 112,151 | 44.7 |  |
|  | Non-metropoli­tan | 216,477 | 31.0 | 71,897 | 15.3 | 144,580 | 55.0 | 201,491 | 30.6 | 64,075 | 15.1 | 137,416 | 55.3 |  |

Note: The treated groups included Gyeonggi, Seoul, Gwangju, Jeonbuk, Chungnam, Incheon, and Jeju, and the never-treated comparison groups included Busan, Daegu, Daejeon, Ulsan, Gangwon, Chungbuk, Jeonnam, Gyeongbuk, and Gyeongnam. All variables except area type were measured by self-report.
