# Supplementary material for: Effects of student human rights ordinances on mental health among middle and high school students in South Korea: a difference-in-differences analysis
Source: Epidemiol Health. 2025 Mar 1;47:e2025011. doi: 10.4178/epih.e2025011 (PMC12062860; doi:10.4178/epih.e2025011)
Supplement: Supplementary Material 5. — Results of pre-treatment parallel trends analysis [file epih-47-e2025011-Supplementary-5.docx]

Supplementary Material 5. Results of pre-treatment parallel trends analysis

| Outcome | | Total | | Male | | Female | |
| --- | --- | --- | --- | --- | --- | --- | --- |
|  |  | χ^2^ | p-value | χ^2^ | p-value | χ^2^ | p-value |
|  | Perceived stress | 108.4 | <0.0001 | 122.0 | <0.0001 | 81.7 | 0.0003 |
|  | Sleep insufficiency | 97.4 | <0.0001 | 272.7 | <0.0001 | 100.5 | <0.0001 |
|  | Depressive mood | 160.8 | <0.0001 | 156.8 | <0.0001 | 124.0 | <0.0001 |
|  | Suicide ideation | 115.0 | <0.0001 | 262.7 | <0.0001 | 57.5 | 0.0689 |
|  | Suicide attempt | 118.8 | <0.0001 | 224.6 | <0.0001 | 168.1 | <0.0001 |

Note: P-value was calculated by Wald test.
