# Supplementary material for: Effects of student human rights ordinances on mental health among middle and high school students in South Korea: a difference-in-differences analysis
Source: Epidemiol Health. 2025 Mar 1;47:e2025011. doi: 10.4178/epih.e2025011 (PMC12062860; doi:10.4178/epih.e2025011)
Supplement: Supplementary Material 6. — Dynamic average treatment effects of student human rights ordinances on mental health among middle and high school students in South Korea [file epih-47-e2025011-Supplementary-6.docx]

Supplementary Material 6. Dynamic average treatment effects of student human rights ordinances on mental health among middle and high school students in South Korea

| Outcome | Time from treatment (year) | Total | | Male | | Female | |
| --- | --- | --- | --- | --- | --- | --- | --- |
|  |  | Average treatment effect on the treated | 95% confidence interval | Average treatment effect on the treated | 95% confidence interval | Average treatment effect on the treated | 95% confidence interval |
| Perceived stress | Pre-treatment average | 0.0024 | (-0.0119, 0.0168) | 0.0053 | (-0.0166, 0.0272) | -0.0047 | (-0.0173, 0.0080) |
|  | Post-treatment average | 0.0038 | (-0.0073, 0.0150) | 0.0103 | (-0.0027, 0.0233) | 0.0000 | (-0.0158, 0.0159) |
|  | -15 | 0.0022 | (-0.0186, 0.0230) | -0.0027 | (-0.0176, 0.0122) | 0.0010 | (-0.0429, 0.0448) |
|  | -14 | 0.0054 | (-0.0169, 0.0277) | 0.0134 | (-0.0219, 0.0487) | -0.0063 | (-0.0300, 0.0174) |
|  | -13 | 0.0081 | (-0.0172, 0.0334) | 0.0090 | (-0.0243, 0.0422) | 0.0047 | (-0.0187, 0.0281) |
|  | -12 | 0.0018 | (-0.0277, 0.0314) | -0.0014 | (-0.0414, 0.0386) | -0.0024 | (-0.0184, 0.0136) |
|  | -11 | -0.0190 | (-0.0525, 0.0145) | -0.0150 | (-0.0634, 0.0335) | -0.0233 | (-0.0493, 0.0026) |
|  | -10 | -0.0058 | (-0.0242, 0.0127) | -0.0005 | (-0.0347, 0.0337) | -0.0306 | (-0.0622, 0.0010) |
|  | -9 | 0.0076 | (-0.0049, 0.0202) | 0.0039 | (-0.0218, 0.0296) | -0.0003 | (-0.0299, 0.0294) |
|  | -8 | 0.0015 | (-0.0196, 0.0227) | 0.0077 | (-0.0197, 0.0351) | -0.0148 | (-0.0388, 0.0092) |
|  | -7 | 0.0230 | (0.0041, 0.0419) | 0.0317 | (-0.0023, 0.0657) | 0.0120 | (-0.0009, 0.0249) |
|  | -6 | -0.0009 | (-0.0136, 0.0118) | 0.0031 | (-0.0114, 0.0177) | -0.0038 | (-0.0222, 0.0146) |
|  | -5 | 0.0081 | (-0.0016, 0.0177) | 0.0170 | (-0.0046, 0.0385) | 0.0017 | (-0.0139, 0.0174) |
|  | -4 | 0.0022 | (-0.0137, 0.0181) | 0.0092 | (-0.0076, 0.0259) | -0.0039 | (-0.0232, 0.0154) |
|  | -3 | -0.0010 | (-0.0178, 0.0158) | 0.0020 | (-0.0150, 0.0191) | -0.0047 | (-0.0279, 0.0186) |
|  | -2 | 0.0008 | (-0.0048, 0.0065) | -0.0032 | (-0.0142, 0.0079) | 0.0051 | (-0.0026, 0.0129) |
|  | 0 | -0.0067 | (-0.0256, 0.0121) | -0.0017 | (-0.0224, 0.0190) | -0.0156 | (-0.0340, 0.0028) |
|  | 1 | -0.0044 | (-0.0163, 0.0075) | -0.0030 | (-0.0241, 0.0181) | -0.0062 | (-0.0220, 0.0096) |
|  | 2 | -0.0015 | (-0.0162, 0.0132) | 0.0021 | (-0.0202, 0.0245) | -0.0037 | (-0.0212, 0.0139) |
|  | 3 | -0.0001 | (-0.0150, 0.0148) | 0.0124 | (-0.0103, 0.0351) | -0.0121 | (-0.0312, 0.0069) |
|  | 4 | -0.0038 | (-0.0260, 0.0183) | -0.0065 | (-0.0238, 0.0108) | -0.0022 | (-0.0412, 0.0368) |
|  | 5 | 0.0022 | (-0.0176, 0.0221) | 0.0059 | (-0.0155, 0.0273) | 0.0026 | (-0.0221, 0.0273) |
|  | 6 | -0.0011 | (-0.0281, 0.0260) | 0.0033 | (-0.0299, 0.0364) | 0.0028 | (-0.0254, 0.0310) |
|  | 7 | 0.0044 | (-0.0103, 0.0192) | 0.0132 | (-0.0026, 0.0290) | 0.0003 | (-0.0199, 0.0204) |
|  | 8 | 0.0064 | (-0.0109, 0.0237) | 0.0123 | (-0.0039, 0.0284) | 0.0026 | (-0.0172, 0.0223) |
|  | 9 | 0.0070 | (-0.0129, 0.0269) | 0.0202 | (0.0020, 0.0383) | -0.0026 | (-0.0304, 0.0252) |
|  | 10 | 0.0133 | (-0.0038, 0.0303) | 0.0242 | (0.0132, 0.0352) | 0.0083 | (-0.0205, 0.0371) |
|  | 11 | 0.0146 | (0.0017, 0.0274) | 0.0202 | (0.0048, 0.0356) | 0.0159 | (0.0008, 0.0310) |
|  | 12 | 0.0198 | (0.0003, 0.0392) | 0.0313 | (0.0096, 0.0531) | 0.0105 | (-0.0135, 0.0345) |
| Sleep insufficiency | Pre-treatment average | -0.0063 | (-0.0225, 0.0098) | -0.0054 | (-0.0259, 0.0152) | 0.0019 | (-0.0213, 0.0251) |
|  | Post-treatment average | 0.0057 | (-0.0302, 0.0417) | 0.0092 | (-0.0321, 0.0505) | -0.0010 | (-0.0434, 0.0415) |
|  | -15 | 0.0039 | (-0.0425, 0.0503) | 0.0030 | (-0.0407, 0.0467) | 0.0151 | (-0.0451, 0.0753) |
|  | -14 | -0.0120 | (-0.0476, 0.0236) | -0.0104 | (-0.0342, 0.0134) | 0.0086 | (-0.0424, 0.0596) |
|  | -13 | -0.0029 | (-0.0297, 0.0239) | 0.0013 | (-0.0244, 0.0271) | 0.0092 | (-0.0383, 0.0568) |
|  | -12 | -0.0085 | (-0.0443, 0.0272) | -0.0118 | (-0.0527, 0.0291) | 0.0116 | (-0.0746, 0.0979) |
|  | -11 | -0.0058 | (-0.0374, 0.0257) | -0.0137 | (-0.0452, 0.0178) | 0.0196 | (-0.0296, 0.0688) |
|  | -10 | -0.0126 | (-0.0446, 0.0195) | -0.0018 | (-0.0369, 0.0332) | -0.0055 | (-0.0511, 0.0402) |
|  | -9 | -0.0045 | (-0.0491, 0.0402) | -0.0224 | (-0.0554, 0.0106) | 0.0284 | (-0.0375, 0.0944) |
|  | -8 | -0.0200 | (-0.0526, 0.0125) | -0.0119 | (-0.0347, 0.0109) | -0.0221 | (-0.0710, 0.0268) |
|  | -7 | -0.0069 | (-0.0484, 0.0347) | -0.0081 | (-0.0658, 0.0495) | -0.0022 | (-0.0390, 0.0347) |
|  | -6 | -0.0018 | (-0.0284, 0.0248) | 0.0092 | (-0.0325, 0.0508) | -0.0102 | (-0.0509, 0.0305) |
|  | -5 | -0.0047 | (-0.0214, 0.0121) | -0.0009 | (-0.0325, 0.0308) | -0.0052 | (-0.0206, 0.0102) |
|  | -4 | -0.0086 | (-0.0223, 0.0052) | -0.0049 | (-0.0321, 0.0223) | -0.0109 | (-0.0363, 0.0146) |
|  | -3 | -0.0091 | (-0.0307, 0.0125) | -0.0060 | (-0.0268, 0.0149) | -0.0153 | (-0.0446, 0.0140) |
|  | -2 | 0.0046 | (-0.0123, 0.0214) | 0.0031 | (-0.0193, 0.0254) | 0.0052 | (-0.0235, 0.0339) |
|  | 0 | -0.0112 | (-0.0369, 0.0146) | -0.0054 | (-0.0474, 0.0367) | -0.0163 | (-0.0432, 0.0106) |
|  | 1 | -0.0083 | (-0.0440, 0.0273) | -0.0041 | (-0.0436, 0.0354) | -0.0145 | (-0.0575, 0.0284) |
|  | 2 | -0.0109 | (-0.0530, 0.0311) | -0.0054 | (-0.0550, 0.0442) | -0.0220 | (-0.0686, 0.0245) |
|  | 3 | -0.0017 | (-0.0387, 0.0352) | 0.0040 | (-0.0392, 0.0472) | -0.0113 | (-0.0507, 0.0280) |
|  | 4 | -0.0108 | (-0.0656, 0.0439) | -0.0130 | (-0.0838, 0.0579) | -0.0163 | (-0.0654, 0.0328) |
|  | 5 | 0.0016 | (-0.0448, 0.0480) | -0.0003 | (-0.0592, 0.0586) | -0.0007 | (-0.0540, 0.0527) |
|  | 6 | 0.0055 | (-0.0418, 0.0528) | 0.0066 | (-0.0603, 0.0735) | -0.0026 | (-0.0563, 0.0511) |
|  | 7 | 0.0170 | (-0.0294, 0.0634) | 0.0190 | (-0.0317, 0.0697) | 0.0158 | (-0.0474, 0.0791) |
|  | 8 | 0.0188 | (-0.0328, 0.0704) | 0.0303 | (-0.0145, 0.0751) | 0.0163 | (-0.0494, 0.0820) |
|  | 9 | 0.0285 | (-0.0164, 0.0735) | 0.0296 | (-0.0123, 0.0715) | 0.0247 | (-0.0528, 0.1023) |
|  | 10 | 0.0299 | (-0.0235, 0.0832) | 0.0307 | (-0.0295, 0.0909) | 0.0125 | (-0.0525, 0.0776) |
|  | 11 | 0.0197 | (-0.0290, 0.0684) | 0.0235 | (-0.0384, 0.0854) | 0.0190 | (-0.0381, 0.0761) |
|  | 12 | -0.0036 | (-0.0333, 0.0261) | 0.0035 | (-0.0314, 0.0385) | -0.0171 | (-0.0513, 0.0171) |
| Depressive mood | Pre-treatment average | 0.0007 | (-0.0103, 0.0118) | -0.0001 | (-0.0211, 0.0208) | 0.0044 | (-0.0099, 0.0187) |
|  | Post-treatment average | 0.0045 | (-0.0037, 0.0128) | 0.0076 | (-0.0058, 0.0210) | 0.0086 | (-0.0091, 0.0263) |
|  | -15 | -0.0006 | (-0.0124, 0.0112) | 0.0041 | (-0.0173, 0.0256) | 0.0031 | (-0.0276, 0.0337) |
|  | -14 | -0.0067 | (-0.0343, 0.0210) | -0.0131 | (-0.0716, 0.0455) | -0.0012 | (-0.0279, 0.0254) |
|  | -13 | -0.0042 | (-0.0283, 0.0199) | -0.0023 | (-0.0485, 0.0438) | 0.0027 | (-0.0254, 0.0309) |
|  | -12 | 0.0000 | (-0.0129, 0.0129) | 0.0047 | (-0.0564, 0.0659) | 0.0060 | (-0.0149, 0.0268) |
|  | -11 | -0.0045 | (-0.0200, 0.0111) | -0.0138 | (-0.0407, 0.0131) | 0.0056 | (-0.0151, 0.0263) |
|  | -10 | 0.0058 | (-0.0048, 0.0165) | 0.0006 | (-0.0327, 0.0339) | 0.0076 | (-0.0063, 0.0214) |
|  | -9 | 0.0060 | (-0.0031, 0.0151) | -0.0026 | (-0.0258, 0.0206) | 0.0119 | (-0.0093, 0.0331) |
|  | -8 | -0.0004 | (-0.0211, 0.0202) | -0.0076 | (-0.0622, 0.0470) | 0.0103 | (-0.0061, 0.0266) |
|  | -7 | 0.0166 | (-0.0093, 0.0424) | 0.0218 | (-0.0088, 0.0524) | 0.0244 | (-0.0021, 0.0510) |
|  | -6 | -0.0024 | (-0.0112, 0.0063) | -0.0001 | (-0.0151, 0.0148) | -0.0062 | (-0.0328, 0.0203) |
|  | -5 | -0.0001 | (-0.0064, 0.0062) | 0.0072 | (-0.0170, 0.0315) | -0.0001 | (-0.0155, 0.0152) |
|  | -4 | -0.0024 | (-0.0112, 0.0064) | -0.0020 | (-0.0222, 0.0183) | -0.0038 | (-0.0150, 0.0073) |
|  | -3 | 0.0025 | (-0.0079, 0.0130) | -0.0014 | (-0.0236, 0.0208) | 0.0050 | (-0.0042, 0.0141) |
|  | -2 | 0.0006 | (-0.0050, 0.0061) | 0.0025 | (-0.0181, 0.0232) | -0.0035 | (-0.0163, 0.0092) |
|  | 0 | -0.0044 | (-0.0117, 0.0028) | -0.0053 | (-0.0167, 0.0062) | 0.0002 | (-0.0207, 0.0211) |
|  | 1 | 0.0061 | (-0.0057, 0.0180) | 0.0067 | (-0.0165, 0.0298) | 0.0075 | (-0.0083, 0.0233) |
|  | 2 | 0.0031 | (-0.0083, 0.0145) | 0.0027 | (-0.0215, 0.0269) | 0.0062 | (-0.0126, 0.0250) |
|  | 3 | 0.0074 | (-0.0049, 0.0197) | 0.0070 | (-0.0117, 0.0256) | 0.0115 | (-0.0046, 0.0276) |
|  | 4 | 0.0073 | (-0.0032, 0.0177) | -0.0011 | (-0.0241, 0.0219) | 0.0144 | (0.0027, 0.0261) |
|  | 5 | 0.0078 | (-0.0034, 0.0189) | 0.0111 | (-0.0056, 0.0278) | 0.0103 | (-0.0075, 0.0282) |
|  | 6 | 0.0076 | (0.0004, 0.0148) | 0.0137 | (-0.0142, 0.0416) | 0.0099 | (-0.0114, 0.0311) |
|  | 7 | 0.0057 | (-0.0079, 0.0194) | 0.0132 | (-0.0059, 0.0322) | 0.0050 | (-0.0285, 0.0384) |
|  | 8 | 0.0044 | (-0.0108, 0.0196) | 0.0030 | (-0.0147, 0.0206) | 0.0091 | (-0.0152, 0.0334) |
|  | 9 | 0.0048 | (-0.0118, 0.0214) | 0.0045 | (-0.0195, 0.0285) | 0.0121 | (-0.0100, 0.0342) |
|  | 10 | 0.0054 | (-0.0126, 0.0235) | 0.0179 | (-0.0096, 0.0453) | 0.0052 | (-0.0224, 0.0329) |
|  | 11 | 0.0010 | (-0.0145, 0.0165) | 0.0130 | (-0.0071, 0.0332) | 0.0036 | (-0.0254, 0.0326) |
|  | 12 | 0.0025 | (-0.0201, 0.0251) | 0.0125 | (-0.0253, 0.0503) | 0.0170 | (-0.0038, 0.0377) |
| Suicide ideation | Pre-treatment average | 0.0047 | (-0.0068, 0.0162) | 0.0071 | (-0.0127, 0.0268) | 0.0026 | (-0.0089, 0.0142) |
|  | Post-treatment average | 0.0079 | (0.0004, 0.0153) | 0.0092 | (-0.0004, 0.0188) | 0.0065 | (-0.0052, 0.0181) |
|  | -15 | 0.0189 | (-0.0041, 0.0420) | 0.0221 | (-0.0191, 0.0632) | 0.0172 | (-0.0116, 0.0459) |
|  | -14 | 0.0035 | (-0.0151, 0.0222) | 0.0066 | (-0.0129, 0.0261) | 0.0010 | (-0.0268, 0.0288) |
|  | -13 | 0.0111 | (-0.0053, 0.0274) | 0.0152 | (-0.0276, 0.0581) | 0.0066 | (-0.0160, 0.0293) |
|  | -12 | 0.0097 | (-0.0110, 0.0304) | 0.0154 | (-0.0162, 0.0471) | 0.0023 | (-0.0179, 0.0224) |
|  | -11 | 0.0000 | (-0.0187, 0.0188) | 0.0039 | (-0.0263, 0.0341) | -0.0033 | (-0.0321, 0.0255) |
|  | -10 | 0.0010 | (-0.0236, 0.0256) | 0.0060 | (-0.0238, 0.0358) | 0.0019 | (-0.0277, 0.0315) |
|  | -9 | -0.0029 | (-0.0239, 0.0180) | 0.0011 | (-0.0217, 0.0239) | -0.0071 | (-0.0332, 0.0191) |
|  | -8 | 0.0062 | (-0.0206, 0.0330) | 0.0073 | (-0.0765, 0.0910) | 0.0091 | (-0.0060, 0.0242) |
|  | -7 | 0.0117 | (0.0023, 0.0210) | 0.0219 | (-0.0022, 0.0459) | 0.0025 | (-0.0184, 0.0234) |
|  | -6 | -0.0015 | (-0.0114, 0.0085) | -0.0024 | (-0.0305, 0.0257) | 0.0003 | (-0.0152, 0.0158) |
|  | -5 | 0.0077 | (-0.0078, 0.0231) | 0.0127 | (-0.0106, 0.0359) | -0.0016 | (-0.0222, 0.0190) |
|  | -4 | -0.0003 | (-0.0092, 0.0085) | -0.0040 | (-0.0230, 0.0150) | 0.0034 | (-0.0050, 0.0117) |
|  | -3 | 0.0014 | (-0.0145, 0.0173) | -0.0048 | (-0.0243, 0.0147) | 0.0068 | (-0.0079, 0.0215) |
|  | -2 | -0.0008 | (-0.0093, 0.0076) | -0.0020 | (-0.0143, 0.0103) | -0.0021 | (-0.0148, 0.0105) |
|  | 0 | 0.0045 | (-0.0063, 0.0154) | 0.0089 | (-0.0072, 0.0249) | -0.0003 | (-0.0172, 0.0166) |
|  | 1 | 0.0055 | (-0.0069, 0.0179) | 0.0065 | (-0.0078, 0.0209) | 0.0058 | (-0.0134, 0.0250) |
|  | 2 | 0.0098 | (-0.0050, 0.0246) | 0.0017 | (-0.0241, 0.0274) | 0.0166 | (0.0027, 0.0305) |
|  | 3 | 0.0049 | (-0.0044, 0.0141) | 0.0032 | (-0.0108, 0.0171) | 0.0065 | (-0.0066, 0.0196) |
|  | 4 | 0.0005 | (-0.0114, 0.0124) | -0.0048 | (-0.0204, 0.0109) | 0.0070 | (-0.0075, 0.0215) |
|  | 5 | 0.0090 | (-0.0033, 0.0212) | 0.0053 | (-0.0057, 0.0164) | 0.0112 | (-0.0056, 0.0280) |
|  | 6 | 0.0097 | (-0.0018, 0.0212) | 0.0119 | (-0.0040, 0.0277) | 0.0066 | (-0.0069, 0.0200) |
|  | 7 | 0.0051 | (-0.0158, 0.0261) | 0.0019 | (-0.0188, 0.0225) | 0.0021 | (-0.0213, 0.0255) |
|  | 8 | 0.0004 | (-0.0168, 0.0175) | 0.0037 | (-0.0127, 0.0201) | -0.0037 | (-0.0305, 0.0231) |
|  | 9 | -0.0006 | (-0.0182, 0.0170) | 0.0093 | (-0.0057, 0.0242) | -0.0123 | (-0.0463, 0.0217) |
|  | 10 | 0.0104 | (-0.0029, 0.0237) | 0.0194 | (-0.0041, 0.0430) | -0.0001 | (-0.0267, 0.0265) |
|  | 11 | 0.0175 | (-0.0000, 0.0350) | 0.0220 | (-0.0037, 0.0476) | 0.0210 | (-0.0057, 0.0478) |
|  | 12 | 0.0256 | (0.0157, 0.0355) | 0.0308 | (0.0179, 0.0436) | 0.0236 | (0.0009, 0.0463) |
| Suicide attempt | Pre-treatment average | 0.0011 | (-0.0031, 0.0052) | 0.0015 | (-0.0024, 0.0054) | 0.0009 | (-0.0065, 0.0082) |
|  | Post-treatment average | -0.0007 | (-0.0032, 0.0018) | -0.0010 | (-0.0087, 0.0067) | 0.0006 | (-0.0034, 0.0046) |
|  | -15 | 0.0071 | (0.0031, 0.0112) | 0.0066 | (0.0001, 0.0132) | 0.0079 | (-0.0034, 0.0192) |
|  | -14 | 0.0029 | (-0.0083, 0.0142) | 0.0073 | (-0.0012, 0.0159) | 0.0027 | (-0.0097, 0.0152) |
|  | -13 | 0.0026 | (-0.0041, 0.0093) | 0.0039 | (-0.0033, 0.0111) | -0.0014 | (-0.0222, 0.0195) |
|  | -12 | 0.0056 | (-0.0082, 0.0195) | 0.0064 | (-0.0016, 0.0144) | 0.0039 | (-0.0158, 0.0237) |
|  | -11 | -0.0013 | (-0.0104, 0.0079) | -0.0006 | (-0.0077, 0.0065) | -0.0021 | (-0.0140, 0.0097) |
|  | -10 | 0.0004 | (-0.0073, 0.0081) | -0.0008 | (-0.0094, 0.0078) | -0.0002 | (-0.0087, 0.0084) |
|  | -9 | 0.0033 | (-0.0068, 0.0135) | 0.0036 | (-0.0060, 0.0132) | -0.0004 | (-0.0182, 0.0174) |
|  | -8 | 0.0010 | (-0.0079, 0.0099) | 0.0028 | (-0.0143, 0.0200) | -0.0029 | (-0.0133, 0.0075) |
|  | -7 | 0.0030 | (-0.0030, 0.0090) | 0.0043 | (0.0007, 0.0080) | -0.0006 | (-0.0106, 0.0095) |
|  | -6 | -0.0018 | (-0.0066, 0.0030) | -0.0003 | (-0.0079, 0.0072) | 0.0011 | (-0.0073, 0.0095) |
|  | -5 | -0.0014 | (-0.0084, 0.0055) | -0.0003 | (-0.0069, 0.0063) | 0.0003 | (-0.0098, 0.0105) |
|  | -4 | -0.0035 | (-0.0083, 0.0013) | -0.0056 | (-0.0136, 0.0025) | 0.0016 | (-0.0023, 0.0054) |
|  | -3 | -0.0016 | (-0.0043, 0.0010) | -0.0049 | (-0.0142, 0.0043) | 0.0024 | (-0.0052, 0.0100) |
|  | -2 | -0.0014 | (-0.0088, 0.0060) | -0.0013 | (-0.0085, 0.0059) | -0.0002 | (-0.0051, 0.0047) |
|  | 0 | -0.0007 | (-0.0032, 0.0017) | -0.0003 | (-0.0101, 0.0095) | 0.0002 | (-0.0066, 0.0071) |
|  | 1 | 0.0019 | (-0.0034, 0.0072) | 0.0018 | (-0.0063, 0.0098) | 0.0025 | (-0.0055, 0.0106) |
|  | 2 | 0.0020 | (-0.0031, 0.0071) | -0.0008 | (-0.0100, 0.0083) | 0.0067 | (0.0018, 0.0117) |
|  | 3 | 0.0002 | (-0.0026, 0.0030) | -0.0006 | (-0.0063, 0.0051) | 0.0000 | (-0.0053, 0.0054) |
|  | 4 | -0.0026 | (-0.0086, 0.0033) | -0.0025 | (-0.0153, 0.0102) | 0.0008 | (-0.0077, 0.0093) |
|  | 5 | -0.0005 | (-0.0036, 0.0026) | -0.0012 | (-0.0103, 0.0080) | 0.0009 | (-0.0045, 0.0064) |
|  | 6 | -0.0024 | (-0.0070, 0.0023) | 0.0002 | (-0.0111, 0.0116) | -0.0029 | (-0.0094, 0.0037) |
|  | 7 | -0.0014 | (-0.0070, 0.0042) | -0.0003 | (-0.0080, 0.0075) | 0.0006 | (-0.0096, 0.0109) |
|  | 8 | -0.0012 | (-0.0068, 0.0045) | -0.0012 | (-0.0141, 0.0117) | -0.0013 | (-0.0097, 0.0071) |
|  | 9 | -0.0033 | (-0.0100, 0.0035) | -0.0019 | (-0.0091, 0.0052) | -0.0044 | (-0.0134, 0.0045) |
|  | 10 | -0.0053 | (-0.0116, 0.0010) | -0.0012 | (-0.0074, 0.0049) | -0.0055 | (-0.0134, 0.0023) |
|  | 11 | 0.0025 | (-0.0017, 0.0067) | -0.0015 | (-0.0102, 0.0071) | 0.0047 | (-0.0026, 0.0119) |
|  | 12 | 0.0019 | (-0.0042, 0.0080) | -0.0032 | (-0.0097, 0.0034) | 0.0049 | (-0.0038, 0.0137) |
