# Supplementary material for: Effects of student human rights ordinances on mental health among middle and high school students in South Korea: a difference-in-differences analysis
Source: Epidemiol Health. 2025 Mar 1;47:e2025011. doi: 10.4178/epih.e2025011 (PMC12062860; doi:10.4178/epih.e2025011)
Supplement: Supplementary Material 7. — Group average treatment effects of student human rights ordinances on mental health among middle and high school students in South Korea [file epih-47-e2025011-Supplementary-7.docx]

Supplementary Material 7. Group average treatment effects of student human rights ordinances on mental health among middle and high school students in South Korea

| Outcome | Group | Total | | Male | | Female | |
| --- | --- | --- | --- | --- | --- | --- | --- |
|  |  | Average treatment effect on the treated | 95% confidence interval | Average treatment effect on the treated | 95% confidence interval | Average treatment effect on the treated | 95% confidence interval |
| Perceived stress | Average | 0.0023 | (-0.0071, 0.0117) | 0.0091 | (-0.0039, 0.0222) | -0.0030 | (-0.0205, 0.0145) |
|  | 2011 | 0.0004 | (-0.0131, 0.0139) | 0.0056 | (-0.0066, 0.0178) | -0.0048 | (-0.0267, 0.0170) |
|  | 2012 | 0.0004 | (-0.0061, 0.0070) | 0.0050 | (-0.0033, 0.0134) | 0.0018 | (-0.0145, 0.0181) |
|  | 2014 | 0.0315 | (0.0223, 0.0407) | 0.0445 | (0.0320, 0.0571) | 0.0197 | (0.0028, 0.0366) |
|  | 2020 | 0.0061 | (-0.0071, 0.0194) | 0.0393 | (0.0264, 0.0521) | -0.0209 | (-0.0402, -0.0017) |
|  | 2021 | -0.0054 | (-0.0168, 0.0059) | -0.0052 | (-0.0192, 0.0089) | -0.0171 | (-0.0398, 0.0055) |
| Sleep insufficiency | Average | 0.0039 | (-0.0228, 0.0306) | 0.0066 | (-0.0255, 0.0387) | -0.0011 | (-0.0293, 0.0270) |
|  | 2011 | -0.0212 | (-0.0413, -0.0012) | -0.0237 | (-0.0393, -0.0081) | -0.0266 | (-0.0514, -0.0017) |
|  | 2012 | 0.0354 | (0.0220, 0.0488) | 0.0452 | (0.0267, 0.0636) | 0.0277 | (0.0057, 0.0497) |
|  | 2014 | 0.0160 | (0.0023, 0.0297) | 0.0390 | (0.0241, 0.0538) | -0.0085 | (-0.0224, 0.0054) |
|  | 2020 | 0.0059 | (-0.0144, 0.0262) | 0.0030 | (-0.0163, 0.0223) | 0.0202 | (-0.0069, 0.0473) |
|  | 2021 | -0.0152 | (-0.0383, 0.0080) | -0.0232 | (-0.0433, -0.0030) | -0.0113 | (-0.0434, 0.0208) |
| Depressive mood | Average | 0.0047 | (-0.0019, 0.0112) | 0.0067 | (-0.0021, 0.0155) | 0.0087 | (-0.0032, 0.0207) |
|  | 2011 | 0.0058 | (-0.0004, 0.0120) | 0.0048 | (-0.0058, 0.0153) | 0.0144 | (0.0068, 0.0220) |
|  | 2012 | 0.0005 | (-0.0103, 0.0112) | 0.0094 | (-0.0010, 0.0199) | -0.0032 | (-0.0195, 0.0131) |
|  | 2014 | 0.0179 | (0.0115, 0.0243) | 0.0068 | (-0.0064, 0.0200) | 0.0261 | (0.0169, 0.0352) |
|  | 2020 | 0.0157 | (0.0088, 0.0225) | 0.0120 | (-0.0024, 0.0264) | 0.0323 | (0.0239, 0.0408) |
|  | 2021 | -0.0007 | (-0.0100, 0.0086) | 0.0018 | (-0.0222, 0.0257) | 0.0034 | (-0.0198, 0.0265) |
| Suicide ideation | Average | 0.0079 | (0.0016, 0.0143) | 0.0088 | (0.0032, 0.0144) | 0.0067 | (-0.0028, 0.0162) |
|  | 2011 | 0.0080 | (0.0009, 0.0152) | 0.0100 | (0.0042, 0.0158) | 0.0066 | (-0.0047, 0.0179) |
|  | 2012 | 0.0044 | (-0.0018, 0.0105) | 0.0048 | (-0.0033, 0.0130) | 0.0024 | (-0.0046, 0.0095) |
|  | 2014 | 0.0074 | (-0.0015, 0.0162) | -0.0014 | (-0.0109, 0.0081) | 0.0182 | (0.0047, 0.0317) |
|  | 2020 | 0.0097 | (0.0021, 0.0174) | 0.0177 | (0.0094, 0.0260) | -0.0018 | (-0.0113, 0.0077) |
|  | 2021 | 0.0192 | (0.0083, 0.0302) | 0.0190 | (0.0048, 0.0332) | 0.0197 | (0.0098, 0.0296) |
| Suicide attempt | Average | -0.0001 | (-0.0022, 0.0020) | 0.0000 | (-0.0049, 0.0049) | 0.0005 | (-0.0035, 0.0045) |
|  | 2011 | -0.0014 | (-0.0035, 0.0006) | -0.0030 | (-0.0071, 0.0010) | 0.0011 | (-0.0030, 0.0051) |
|  | 2012 | 0.0002 | (-0.0016, 0.0020) | 0.0024 | (-0.0030, 0.0079) | -0.0001 | (-0.0053, 0.0051) |
|  | 2014 | -0.0035 | (-0.0065, -0.0004) | -0.0093 | (-0.0118, -0.0067) | 0.0027 | (-0.0035, 0.0089) |
|  | 2020 | 0.0021 | (-0.0008, 0.0051) | 0.0078 | (0.0046, 0.0111) | -0.0074 | (-0.0123, -0.0025) |
|  | 2021 | 0.0051 | (-0.0024, 0.0125) | 0.0050 | (0.0014, 0.0085) | 0.0039 | (-0.0072, 0.0151) |
