# Supplementary material for: Effects of student human rights ordinances on mental health among middle and high school students in South Korea: a difference-in-differences analysis
Source: Epidemiol Health. 2025 Mar 1;47:e2025011. doi: 10.4178/epih.e2025011 (PMC12062860; doi:10.4178/epih.e2025011)
Supplement: Supplementary Material 8. — Time average treatment effects of student human rights ordinances on mental health among middle and high school students in South Korea [file epih-47-e2025011-Supplementary-8.docx]

Supplementary Material 8. Time average treatment effects of student human rights ordinances on mental health among middle and high school students in South Korea

| Outcome | Year | Total | | Male | | Female | |
| --- | --- | --- | --- | --- | --- | --- | --- |
|  |  | Average treatment effect on the treated | 95% confidence interval | Average treatment effect on the treated | 95% confidence interval | Average treatment effect on the treated | 95% confidence interval |
| Perceived stress | Average | 0.0019 | (-0.0083, 0.0121) | 0.0076 | (-0.0032, 0.0184) | -0.0018 | (-0.0163, 0.0127) |
|  | 2011 | -0.0045 | (-0.0156, 0.0065) | -0.0019 | (-0.0145, 0.0106) | -0.0122 | (-0.0259, 0.0014) |
|  | 2012 | -0.0119 | (-0.0311, 0.0073) | -0.0099 | (-0.0273, 0.0076) | -0.0184 | (-0.0401, 0.0033) |
|  | 2013 | -0.0099 | (-0.0222, 0.0024) | -0.0134 | (-0.0292, 0.0025) | -0.0042 | (-0.0197, 0.0112) |
|  | 2014 | 0.0016 | (-0.0139, 0.0171) | 0.0117 | (-0.0057, 0.0291) | -0.0078 | (-0.0345, 0.0189) |
|  | 2015 | -0.0072 | (-0.0246, 0.0101) | -0.0035 | (-0.0259, 0.0188) | -0.0119 | (-0.0388, 0.0150) |
|  | 2016 | 0.0061 | (-0.0122, 0.0243) | 0.0029 | (-0.0125, 0.0183) | 0.0126 | (-0.0184, 0.0437) |
|  | 2017 | -0.0059 | (-0.0286, 0.0169) | 0.0001 | (-0.0313, 0.0315) | -0.0054 | (-0.0317, 0.0209) |
|  | 2018 | 0.0047 | (-0.0092, 0.0187) | 0.0099 | (-0.0073, 0.0271) | 0.0076 | (-0.0142, 0.0294) |
|  | 2019 | 0.0095 | (-0.0093, 0.0284) | 0.0158 | (-0.0008, 0.0325) | 0.0035 | (-0.0222, 0.0291) |
|  | 2020 | 0.0092 | (-0.0081, 0.0265) | 0.0212 | (0.0005, 0.0420) | 0.0032 | (-0.0191, 0.0256) |
|  | 2021 | 0.0038 | (-0.0179, 0.0254) | 0.0185 | (0.0020, 0.0350) | -0.0094 | (-0.0440, 0.0252) |
|  | 2022 | 0.0133 | (-0.0016, 0.0282) | 0.0217 | (0.0097, 0.0338) | 0.0094 | (-0.0164, 0.0352) |
|  | 2023 | 0.0157 | (0.0012, 0.0302) | 0.0255 | (0.0067, 0.0444) | 0.0094 | (-0.0090, 0.0279) |
| Sleep insufficiency | Average | 0.0029 | (-0.0291, 0.0349) | 0.0059 | (-0.0298, 0.0416) | -0.0031 | (-0.0371, 0.0309) |
|  | 2011 | -0.0284 | (-0.0375, -0.0193) | -0.0356 | (-0.0501, -0.0210) | -0.0252 | (-0.0411, -0.0093) |
|  | 2012 | -0.0149 | (-0.0511, 0.0213) | -0.0076 | (-0.0533, 0.0381) | -0.0220 | (-0.0572, 0.0132) |
|  | 2013 | -0.0156 | (-0.0635, 0.0323) | -0.0108 | (-0.0580, 0.0364) | -0.0269 | (-0.0756, 0.0218) |
|  | 2014 | -0.0069 | (-0.0468, 0.0330) | 0.0000 | (-0.0531, 0.0531) | -0.0163 | (-0.0543, 0.0217) |
|  | 2015 | -0.0118 | (-0.0651, 0.0415) | -0.0062 | (-0.0752, 0.0629) | -0.0257 | (-0.0697, 0.0183) |
|  | 2016 | -0.0002 | (-0.0519, 0.0515) | -0.0024 | (-0.0669, 0.0622) | -0.0039 | (-0.0429, 0.0351) |
|  | 2017 | -0.0026 | (-0.0470, 0.0418) | -0.0003 | (-0.0575, 0.0569) | -0.0095 | (-0.0666, 0.0475) |
|  | 2018 | 0.0130 | (-0.0227, 0.0487) | 0.0157 | (-0.0320, 0.0634) | 0.0047 | (-0.0404, 0.0497) |
|  | 2019 | 0.0262 | (-0.0335, 0.0859) | 0.0289 | (-0.0272, 0.0851) | 0.0283 | (-0.0401, 0.0968) |
|  | 2020 | 0.0263 | (-0.0169, 0.0695) | 0.0352 | (-0.0041, 0.0744) | 0.0189 | (-0.0356, 0.0733) |
|  | 2021 | 0.0174 | (-0.0355, 0.0704) | 0.0148 | (-0.0328, 0.0624) | 0.0130 | (-0.0662, 0.0923) |
|  | 2022 | 0.0178 | (-0.0252, 0.0607) | 0.0229 | (-0.0442, 0.0901) | 0.0098 | (-0.0270, 0.0466) |
|  | 2023 | 0.0174 | (-0.0295, 0.0643) | 0.0220 | (-0.0247, 0.0687) | 0.0143 | (-0.0429, 0.0715) |
| Depressive mood | Average | 0.0043 | (-0.0031, 0.0117) | 0.0062 | (-0.0030, 0.0154) | 0.0082 | (-0.0065, 0.0229) |
|  | 2011 | -0.0011 | (-0.0075, 0.0054) | -0.0069 | (-0.0215, 0.0077) | 0.0107 | (0.0030, 0.0183) |
|  | 2012 | -0.0060 | (-0.0178, 0.0057) | -0.0054 | (-0.0191, 0.0083) | -0.0063 | (-0.0260, 0.0135) |
|  | 2013 | 0.0039 | (-0.0087, 0.0164) | 0.0033 | (-0.0206, 0.0272) | 0.0019 | (-0.0125, 0.0163) |
|  | 2014 | 0.0022 | (-0.0105, 0.0148) | 0.0053 | (-0.0145, 0.0252) | 0.0044 | (-0.0129, 0.0218) |
|  | 2015 | 0.0083 | (-0.0063, 0.0228) | 0.0009 | (-0.0220, 0.0238) | 0.0165 | (0.0019, 0.0311) |
|  | 2016 | 0.0108 | (0.0029, 0.0186) | 0.0091 | (-0.0033, 0.0214) | 0.0160 | (0.0048, 0.0272) |
|  | 2017 | 0.0062 | (-0.0058, 0.0181) | 0.0090 | (-0.0100, 0.0279) | 0.0120 | (-0.0074, 0.0313) |
|  | 2018 | 0.0103 | (0.0016, 0.0190) | 0.0181 | (0.0005, 0.0356) | 0.0105 | (-0.0092, 0.0302) |
|  | 2019 | 0.0076 | (-0.0079, 0.0230) | 0.0108 | (-0.0040, 0.0255) | 0.0074 | (-0.0264, 0.0413) |
|  | 2020 | 0.0044 | (-0.0099, 0.0187) | 0.0058 | (-0.0103, 0.0218) | 0.0088 | (-0.0143, 0.0320) |
|  | 2021 | 0.0050 | (-0.0132, 0.0231) | 0.0114 | (-0.0155, 0.0383) | 0.0098 | (-0.0092, 0.0289) |
|  | 2022 | 0.0009 | (-0.0110, 0.0129) | 0.0110 | (-0.0022, 0.0241) | 0.0034 | (-0.0177, 0.0244) |
|  | 2023 | 0.0036 | (-0.0134, 0.0207) | 0.0089 | (-0.0190, 0.0368) | 0.0115 | (-0.0111, 0.0341) |
| Suicide ideation | Average | 0.0070 | (0.0008, 0.0131) | 0.0076 | (0.0010, 0.0142) | 0.0058 | (-0.0032, 0.0149) |
|  | 2011 | 0.0113 | (0.0062, 0.0164) | 0.0124 | (0.0060, 0.0187) | 0.0080 | (0.0014, 0.0147) |
|  | 2012 | -0.0030 | (-0.0168, 0.0108) | 0.0053 | (-0.0076, 0.0181) | -0.0099 | (-0.0268, 0.0070) |
|  | 2013 | 0.0044 | (-0.0073, 0.0161) | -0.0082 | (-0.0249, 0.0084) | 0.0164 | (0.0032, 0.0295) |
|  | 2014 | 0.0078 | (-0.0034, 0.0191) | 0.0067 | (-0.0069, 0.0203) | 0.0083 | (-0.0036, 0.0201) |
|  | 2015 | 0.0024 | (-0.0087, 0.0135) | -0.0005 | (-0.0112, 0.0102) | 0.0075 | (-0.0053, 0.0202) |
|  | 2016 | 0.0067 | (-0.0030, 0.0164) | 0.0019 | (-0.0072, 0.0110) | 0.0106 | (-0.0047, 0.0259) |
|  | 2017 | 0.0086 | (-0.0030, 0.0203) | 0.0078 | (-0.0013, 0.0169) | 0.0080 | (-0.0090, 0.0249) |
|  | 2018 | 0.0115 | (-0.0004, 0.0234) | 0.0071 | (-0.0073, 0.0215) | 0.0114 | (-0.0019, 0.0246) |
|  | 2019 | 0.0020 | (-0.0122, 0.0162) | 0.0018 | (-0.0126, 0.0162) | 0.0002 | (-0.0178, 0.0182) |
|  | 2020 | 0.0002 | (-0.0145, 0.0149) | 0.0089 | (-0.0060, 0.0238) | -0.0092 | (-0.0285, 0.0101) |
|  | 2021 | 0.0017 | (-0.0141, 0.0175) | 0.0176 | (0.0018, 0.0334) | -0.0166 | (-0.0377, 0.0046) |
|  | 2022 | 0.0184 | (0.0045, 0.0323) | 0.0229 | (0.0016, 0.0442) | 0.0204 | (0.0013, 0.0395) |
|  | 2023 | 0.0182 | (0.0046, 0.0319) | 0.0154 | (-0.0032, 0.0341) | 0.0210 | (0.0011, 0.0408) |
| Suicide attempt | Average | -0.0006 | (-0.0025, 0.0012) | -0.0009 | (-0.0067, 0.0049) | 0.0008 | (-0.0024, 0.0041) |
|  | 2011 | -0.0008 | (-0.0034, 0.0017) | -0.0046 | (-0.0078, -0.0014) | 0.0060 | (0.0025, 0.0095) |
|  | 2012 | -0.0020 | (-0.0060, 0.0020) | 0.0005 | (-0.0053, 0.0064) | -0.0040 | (-0.0116, 0.0036) |
|  | 2013 | 0.0022 | (-0.0036, 0.0080) | -0.0008 | (-0.0112, 0.0095) | 0.0083 | (0.0041, 0.0125) |
|  | 2014 | 0.0021 | (-0.0011, 0.0052) | -0.0007 | (-0.0081, 0.0066) | 0.0045 | (-0.0029, 0.0120) |
|  | 2015 | -0.0031 | (-0.0096, 0.0034) | -0.0027 | (-0.0090, 0.0036) | -0.0008 | (-0.0085, 0.0069) |
|  | 2016 | 0.0005 | (-0.0034, 0.0045) | 0.0000 | (-0.0071, 0.0071) | 0.0024 | (-0.0010, 0.0059) |
|  | 2017 | -0.0026 | (-0.0062, 0.0011) | -0.0017 | (-0.0101, 0.0067) | -0.0019 | (-0.0067, 0.0028) |
|  | 2018 | -0.0001 | (-0.0043, 0.0041) | 0.0019 | (-0.0060, 0.0098) | 0.0022 | (-0.0100, 0.0144) |
|  | 2019 | -0.0007 | (-0.0059, 0.0045) | -0.0022 | (-0.0084, 0.0041) | -0.0005 | (-0.0092, 0.0083) |
|  | 2020 | -0.0012 | (-0.0038, 0.0015) | 0.0003 | (-0.0092, 0.0098) | -0.0043 | (-0.0104, 0.0019) |
|  | 2021 | -0.0054 | (-0.0105, -0.0003) | 0.0006 | (-0.0043, 0.0054) | -0.0067 | (-0.0142, 0.0009) |
|  | 2022 | 0.0005 | (-0.0044, 0.0053) | -0.0018 | (-0.0081, 0.0045) | 0.0000 | (-0.0075, 0.0076) |
|  | 2023 | 0.0022 | (-0.0022, 0.0067) | -0.0011 | (-0.0068, 0.0046) | 0.0051 | (-0.0023, 0.0125) |
