# Supplementary material for: Effects of student human rights ordinances on mental health among middle and high school students in South Korea: a difference-in-differences analysis
Source: Epidemiol Health. 2025 Mar 1;47:e2025011. doi: 10.4178/epih.e2025011 (PMC12062860; doi:10.4178/epih.e2025011)
Supplement: Supplementary Material 9. — Group-time average treatment effects of student human rights ordinances on mental health among middle and high school students in South Korea [file epih-47-e2025011-Supplementary-9.docx]

Supplementary Material 9. Group-time average treatment effects of student human rights ordinances on mental health among middle and high school students in South Korea

| Outcome | Group | Year | Total | | Male | | Female | |
| --- | --- | --- | --- | --- | --- | --- | --- | --- |
|  |  |  | Average treatment effect on the treated | 95% confidence interval | Average treatment effect on the treated | 95% confidence interval | Average treatment effect on the treated | 95% confidence interval |
| Perceived stress | 2011 | 2006 | 0.0104 | (-0.0024, 0.0231) | 0.0299 | (0.0154, 0.0445) | -0.0050 | (-0.0194, 0.0094) |
|  |  | 2007 | 0.0014 | (-0.0119, 0.0147) | 0.0032 | (-0.0122, 0.0186) | 0.0002 | (-0.0184, 0.0189) |
|  |  | 2008 | 0.0093 | (-0.0043, 0.0229) | 0.0039 | (-0.0134, 0.0212) | 0.0155 | (-0.0027, 0.0337) |
|  |  | 2009 | 0.0048 | (-0.0085, 0.0181) | 0.0048 | (-0.0074, 0.0170) | 0.0060 | (-0.0125, 0.0246) |
|  |  | 2011 | -0.0045 | (-0.0166, 0.0075) | -0.0019 | (-0.0158, 0.0120) | -0.0122 | (-0.0276, 0.0031) |
|  |  | 2012 | -0.0077 | (-0.0282, 0.0128) | -0.0082 | (-0.0246, 0.0082) | -0.0141 | (-0.0418, 0.0136) |
|  |  | 2013 | -0.0080 | (-0.0233, 0.0073) | -0.0104 | (-0.0271, 0.0063) | -0.0067 | (-0.0294, 0.0161) |
|  |  | 2014 | -0.0029 | (-0.0220, 0.0162) | 0.0092 | (-0.0107, 0.0291) | -0.0206 | (-0.0442, 0.0031) |
|  |  | 2015 | -0.0165 | (-0.0308, -0.0022) | -0.0140 | (-0.0337, 0.0057) | -0.0265 | (-0.0496, -0.0035) |
|  |  | 2016 | 0.0019 | (-0.0198, 0.0235) | 0.0031 | (-0.0159, 0.0220) | 0.0031 | (-0.0343, 0.0404) |
|  |  | 2017 | -0.0146 | (-0.0362, 0.0069) | -0.0118 | (-0.0379, 0.0143) | -0.0117 | (-0.0437, 0.0204) |
|  |  | 2018 | 0.0014 | (-0.0188, 0.0217) | 0.0088 | (-0.0153, 0.0328) | -0.0001 | (-0.0251, 0.0249) |
|  |  | 2019 | 0.0096 | (-0.0131, 0.0322) | 0.0117 | (-0.0100, 0.0334) | 0.0057 | (-0.0264, 0.0378) |
|  |  | 2020 | 0.0109 | (-0.0112, 0.0330) | 0.0248 | (0.0029, 0.0467) | 0.0007 | (-0.0263, 0.0277) |
|  |  | 2021 | 0.0100 | (-0.0119, 0.0319) | 0.0217 | (0.0091, 0.0343) | 0.0004 | (-0.0362, 0.0370) |
|  |  | 2022 | 0.0126 | (-0.0064, 0.0316) | 0.0171 | (0.0023, 0.0320) | 0.0155 | (-0.0102, 0.0413) |
|  |  | 2023 | 0.0198 | (-0.0012, 0.0407) | 0.0313 | (0.0088, 0.0538) | 0.0105 | (-0.0162, 0.0373) |
|  | 2012 | 2006 | 0.0020 | (-0.0179, 0.0219) | 0.0054 | (-0.0087, 0.0195) | 0.0044 | (-0.0230, 0.0317) |
|  |  | 2007 | 0.0021 | (-0.0090, 0.0132) | 0.0073 | (-0.0046, 0.0192) | -0.0023 | (-0.0247, 0.0201) |
|  |  | 2008 | -0.0077 | (-0.0222, 0.0068) | 0.0035 | (-0.0142, 0.0213) | -0.0166 | (-0.0409, 0.0078) |
|  |  | 2009 | -0.0103 | (-0.0289, 0.0082) | -0.0035 | (-0.0261, 0.0191) | -0.0182 | (-0.0399, 0.0035) |
|  |  | 2010 | -0.0034 | (-0.0203, 0.0136) | -0.0085 | (-0.0275, 0.0106) | 0.0030 | (-0.0212, 0.0272) |
|  |  | 2012 | -0.0165 | (-0.0376, 0.0046) | -0.0118 | (-0.0393, 0.0158) | -0.0230 | (-0.0464, 0.0004) |
|  |  | 2013 | -0.0120 | (-0.0254, 0.0013) | -0.0167 | (-0.0359, 0.0025) | -0.0016 | (-0.0235, 0.0203) |
|  |  | 2014 | 0.0033 | (-0.0119, 0.0185) | 0.0105 | (-0.0146, 0.0357) | 0.0036 | (-0.0199, 0.0271) |
|  |  | 2015 | -0.0036 | (-0.0190, 0.0119) | 0.0004 | (-0.0147, 0.0156) | -0.0025 | (-0.0253, 0.0203) |
|  |  | 2016 | 0.0073 | (-0.0125, 0.0271) | -0.0024 | (-0.0208, 0.0161) | 0.0215 | (-0.0096, 0.0525) |
|  |  | 2017 | -0.0050 | (-0.0184, 0.0085) | -0.0004 | (-0.0308, 0.0301) | -0.0016 | (-0.0323, 0.0292) |
|  |  | 2018 | 0.0066 | (-0.0047, 0.0179) | 0.0095 | (-0.0094, 0.0283) | 0.0145 | (-0.0030, 0.0320) |
|  |  | 2019 | 0.0030 | (-0.0136, 0.0196) | 0.0127 | (-0.0001, 0.0255) | -0.0028 | (-0.0344, 0.0289) |
|  |  | 2020 | -0.0026 | (-0.0182, 0.0130) | 0.0060 | (-0.0208, 0.0327) | -0.0027 | (-0.0273, 0.0218) |
|  |  | 2021 | -0.0036 | (-0.0228, 0.0156) | 0.0103 | (-0.0059, 0.0265) | -0.0141 | (-0.0497, 0.0215) |
|  |  | 2022 | 0.0171 | (0.0021, 0.0321) | 0.0272 | (0.0135, 0.0409) | 0.0172 | (-0.0072, 0.0416) |
|  |  | 2023 | 0.0168 | (0.0072, 0.0264) | 0.0238 | (0.0061, 0.0414) | 0.0163 | (-0.0031, 0.0356) |
|  | 2014 | 2006 | 0.0103 | (-0.0061, 0.0268) | 0.0055 | (-0.0142, 0.0252) | 0.0039 | (-0.0217, 0.0295) |
|  |  | 2007 | 0.0285 | (0.0151, 0.0419) | 0.0398 | (0.0183, 0.0612) | 0.0097 | (-0.0107, 0.0301) |
|  |  | 2008 | 0.0035 | (-0.0130, 0.0200) | 0.0059 | (-0.0211, 0.0329) | -0.0055 | (-0.0210, 0.0100) |
|  |  | 2009 | 0.0282 | (0.0137, 0.0427) | 0.0462 | (0.0283, 0.0641) | 0.0031 | (-0.0238, 0.0299) |
|  |  | 2010 | 0.0313 | (0.0144, 0.0482) | 0.0375 | (0.0174, 0.0575) | 0.0289 | (0.0052, 0.0526) |
|  |  | 2011 | -0.0027 | (-0.015, 0.0096) | 0.0209 | (-0.0011, 0.0428) | -0.0301 | (-0.0505, -0.0097) |
|  |  | 2012 | 0.0080 | (-0.0094, 0.0253) | 0.0065 | (-0.0111, 0.0241) | 0.0043 | (-0.0212, 0.0298) |
|  |  | 2014 | 0.0187 | (0.0044, 0.0331) | 0.0323 | (0.0136, 0.0510) | 0.0058 | (-0.016, 0.0276) |
|  |  | 2015 | 0.0283 | (0.0203, 0.0363) | 0.0382 | (0.0211, 0.0554) | 0.0231 | (0.0044, 0.0418) |
|  |  | 2016 | 0.0244 | (0.0121, 0.0367) | 0.0285 | (0.0101, 0.0469) | 0.0216 | (-0.0079, 0.051) |
|  |  | 2017 | 0.0417 | (0.0235, 0.0599) | 0.0738 | (0.0506, 0.0969) | 0.0107 | (-0.0121, 0.0336) |
|  |  | 2018 | 0.0149 | (-0.0001, 0.0299) | 0.0188 | (-0.0051, 0.0428) | 0.0166 | (-0.0065, 0.0397) |
|  |  | 2019 | 0.0435 | (0.0257, 0.0613) | 0.0562 | (0.0327, 0.0797) | 0.0232 | (0.0041, 0.0423) |
|  |  | 2020 | 0.0422 | (0.0292, 0.0551) | 0.0652 | (0.0458, 0.0845) | 0.0281 | (0.0094, 0.0467) |
|  |  | 2021 | 0.0309 | (0.0090, 0.0527) | 0.0428 | (0.0167, 0.0689) | 0.0194 | (-0.0136, 0.0523) |
|  |  | 2022 | 0.0344 | (0.0175, 0.0513) | 0.0496 | (0.0326, 0.0666) | 0.0119 | (-0.0178, 0.0416) |
|  |  | 2023 | 0.0391 | (0.0249, 0.0532) | 0.0431 | (0.0205, 0.0657) | 0.0390 | (0.0184, 0.0596) |
|  | 2020 | 2006 | 0.0186 | (-0.0025, 0.0396) | 0.0358 | (0.0124, 0.0591) | 0.0021 | (-0.0291, 0.0334) |
|  |  | 2007 | 0.0172 | (0.0034, 0.0309) | 0.0233 | (0.0057, 0.0408) | 0.0140 | (-0.0071, 0.0351) |
|  |  | 2008 | 0.0160 | (-0.0065, 0.0384) | 0.0224 | (-0.0098, 0.0546) | -0.0060 | (-0.0248, 0.0129) |
|  |  | 2009 | -0.0085 | (-0.0304, 0.0134) | 0.0040 | (-0.0178, 0.0258) | -0.0270 | (-0.0579, 0.0039) |
|  |  | 2010 | 0.0038 | (-0.0215, 0.0291) | 0.0305 | (0.0055, 0.0555) | -0.0462 | (-0.0790, -0.0134) |
|  |  | 2011 | 0.0047 | (-0.0126, 0.0220) | 0.0137 | (-0.0002, 0.0275) | -0.0098 | (-0.0410, 0.0213) |
|  |  | 2012 | 0.0162 | (-0.0013, 0.0336) | 0.0392 | (0.0228, 0.0555) | -0.0194 | (-0.0426, 0.0037) |
|  |  | 2013 | 0.0343 | (0.0183, 0.0504) | 0.0531 | (0.0323, 0.0740) | 0.0222 | (0.0006, 0.0438) |
|  |  | 2014 | -0.0044 | (-0.0256, 0.0168) | 0.0169 | (-0.0093, 0.0431) | -0.0299 | (-0.0504, -0.0095) |
|  |  | 2015 | 0.0074 | (-0.0105, 0.0254) | 0.0150 | (-0.0006, 0.0305) | 0.0067 | (-0.0160, 0.0295) |
|  |  | 2016 | 0.0293 | (0.0127, 0.0459) | 0.0409 | (0.0202, 0.0616) | 0.0107 | (-0.0156, 0.0371) |
|  |  | 2017 | 0.0032 | (-0.0201, 0.0264) | 0.0061 | (-0.0302, 0.0424) | 0.0068 | (-0.0144, 0.0279) |
|  |  | 2018 | -0.0054 | (-0.0219, 0.0111) | -0.0039 | (-0.0258, 0.0180) | -0.0109 | (-0.0324, 0.0107) |
|  |  | 2020 | 0.0281 | (0.0162, 0.0400) | 0.0334 | (0.0179, 0.0490) | 0.0267 | (0.0057, 0.0476) |
|  |  | 2021 | 0.0126 | (-0.0063, 0.0315) | 0.0596 | (0.0390, 0.0801) | -0.0193 | (-0.0421, 0.0035) |
|  |  | 2022 | -0.0110 | (-0.0325, 0.0104) | 0.0258 | (0.0120, 0.0396) | -0.0541 | (-0.0842, -0.0240) |
|  |  | 2023 | -0.0058 | (-0.0252, 0.0137) | 0.0376 | (0.0130, 0.0622) | -0.0377 | (-0.0546, -0.0209) |
|  | 2021 | 2006 | 0.0022 | (-0.0202, 0.0247) | -0.0027 | (-0.0181, 0.0127) | 0.0010 | (-0.0479, 0.0498) |
|  |  | 2007 | -0.0019 | (-0.0179, 0.0142) | 0.0008 | (-0.0352, 0.0368) | -0.0109 | (-0.0328, 0.0111) |
|  |  | 2008 | 0.0030 | (-0.0253, 0.0312) | 0.0006 | (-0.0290, 0.0302) | -0.0003 | (-0.0308, 0.0301) |
|  |  | 2009 | -0.0063 | (-0.0276, 0.0150) | -0.0154 | (-0.0406, 0.0098) | -0.0003 | (-0.0250, 0.0244) |
|  |  | 2010 | -0.0252 | (-0.0641, 0.0137) | -0.0263 | (-0.0849, 0.0322) | -0.0213 | (-0.0636, 0.0210) |
|  |  | 2011 | -0.0118 | (-0.0296, 0.0060) | -0.0210 | (-0.0407, -0.0013) | -0.0213 | (-0.0631, 0.0206) |
|  |  | 2012 | 0.0095 | (-0.0065, 0.0254) | -0.0020 | (-0.0349, 0.0308) | 0.0058 | (-0.0495, 0.0610) |
|  |  | 2013 | -0.0135 | (-0.0282, 0.0012) | -0.0113 | (-0.0330, 0.0104) | -0.0249 | (-0.0449, -0.0048) |
|  |  | 2014 | 0.0122 | (-0.0106, 0.0350) | 0.0127 | (-0.0230, 0.0485) | 0.0076 | (-0.0152, 0.0304) |
|  |  | 2015 | -0.0127 | (-0.0388, 0.0135) | -0.0160 | (-0.0409, 0.0088) | -0.0170 | (-0.0597, 0.0257) |
|  |  | 2016 | 0.0084 | (-0.0117, 0.0285) | -0.0130 | (-0.0290, 0.0030) | 0.0378 | (-0.0083, 0.0838) |
|  |  | 2017 | 0.0057 | (-0.0316, 0.0430) | 0.0131 | (-0.0229, 0.0491) | -0.0022 | (-0.0548, 0.0503) |
|  |  | 2018 | -0.0055 | (-0.0230, 0.0120) | 0.0008 | (-0.0228, 0.0244) | -0.0163 | (-0.0638, 0.0312) |
|  |  | 2019 | 0.0004 | (-0.0182, 0.0190) | -0.0204 | (-0.0341, -0.0067) | 0.0210 | (-0.0106, 0.0527) |
|  |  | 2021 | -0.0176 | (-0.0325, -0.0026) | -0.0086 | (-0.0199, 0.0027) | -0.0417 | (-0.0864, 0.0031) |
|  |  | 2022 | 0.0058 | (-0.0127, 0.0243) | 0.0019 | (-0.0214, 0.0252) | -0.0027 | (-0.0472, 0.0419) |
|  |  | 2023 | -0.0041 | (-0.0243, 0.0161) | -0.0086 | (-0.0473, 0.0301) | -0.0069 | (-0.0452, 0.0314) |
| Sleep insufficiency | 2011 | 2006 | -0.0049 | (-0.0210, 0.0113) | -0.0090 | (-0.0367, 0.0186) | -0.0056 | (-0.0252, 0.0141) |
|  |  | 2007 | -0.0106 | (-0.0224, 0.0012) | -0.0202 | (-0.0388, -0.0016) | -0.0022 | (-0.0141, 0.0096) |
|  |  | 2008 | -0.0039 | (-0.0172, 0.0094) | -0.0062 | (-0.0209, 0.0084) | -0.0082 | (-0.0312, 0.0147) |
|  |  | 2009 | 0.0148 | (-0.0056, 0.0351) | 0.0144 | (0.0016, 0.0273) | 0.0126 | (-0.0239, 0.0491) |
|  |  | 2011 | -0.0284 | (-0.0364, -0.0204) | -0.0356 | (-0.0495, -0.0216) | -0.0252 | (-0.0398, -0.0107) |
|  |  | 2012 | -0.0313 | (-0.0477, -0.0149) | -0.0317 | (-0.0487, -0.0148) | -0.0345 | (-0.0573, -0.0118) |
|  |  | 2013 | -0.0385 | (-0.0553, -0.0216) | -0.0399 | (-0.0532, -0.0266) | -0.0496 | (-0.0745, -0.0247) |
|  |  | 2014 | -0.0273 | (-0.0468, -0.0077) | -0.0310 | (-0.0479, -0.014) | -0.0289 | (-0.0581, 0.0003) |
|  |  | 2015 | -0.0510 | (-0.0716, -0.0304) | -0.0578 | (-0.0791, -0.0365) | -0.0550 | (-0.0799, -0.0301) |
|  |  | 2016 | -0.0300 | (-0.0501, -0.0098) | -0.0383 | (-0.0633, -0.0132) | -0.0297 | (-0.0536, -0.0057) |
|  |  | 2017 | -0.0346 | (-0.0591, -0.0101) | -0.0379 | (-0.063, -0.0127) | -0.0382 | (-0.0700, -0.0064) |
|  |  | 2018 | -0.0161 | (-0.0346, 0.0025) | -0.0165 | (-0.0336, 0.0006) | -0.0198 | (-0.0422, 0.0027) |
|  |  | 2019 | -0.0019 | (-0.0341, 0.0304) | 0.0014 | (-0.0293, 0.0321) | -0.0006 | (-0.0394, 0.0382) |
|  |  | 2020 | 0.0083 | (-0.0155, 0.0322) | 0.0053 | (-0.0189, 0.0295) | 0.0013 | (-0.0332, 0.0357) |
|  |  | 2021 | -0.0004 | (-0.0271, 0.0263) | -0.0107 | (-0.0372, 0.0159) | -0.0188 | (-0.0614, 0.0238) |
|  |  | 2022 | -0.0121 | (-0.0338, 0.0097) | -0.0093 | (-0.0435, 0.0248) | -0.0218 | (-0.0474, 0.0037) |
|  |  | 2023 | -0.0036 | (-0.0296, 0.0224) | 0.0035 | (-0.0288, 0.0359) | -0.0171 | (-0.0446, 0.0103) |
|  | 2012 | 2006 | 0.0117 | (-0.0042, 0.0275) | 0.0316 | (0.0104, 0.0527) | -0.0033 | (-0.0364, 0.0298) |
|  |  | 2007 | 0.0035 | (-0.0077, 0.0148) | 0.0175 | (-0.0106, 0.0456) | -0.0029 | (-0.0294, 0.0235) |
|  |  | 2008 | -0.0060 | (-0.0238, 0.0118) | 0.0089 | (-0.0151, 0.0329) | -0.0217 | (-0.0414, -0.0021) |
|  |  | 2009 | -0.0175 | (-0.0496, 0.0147) | -0.0050 | (-0.0424, 0.0324) | -0.0329 | (-0.0646, -0.0011) |
|  |  | 2010 | 0.0057 | (-0.0139, 0.0254) | 0.0029 | (-0.0311, 0.0369) | 0.0092 | (-0.0109, 0.0292) |
|  |  | 2012 | 0.0033 | (-0.0158, 0.0224) | 0.0198 | (-0.0071, 0.0467) | -0.0085 | (-0.0319, 0.0150) |
|  |  | 2013 | 0.0099 | (-0.0204, 0.0402) | 0.0222 | (-0.0056, 0.0499) | -0.0022 | (-0.0318, 0.0274) |
|  |  | 2014 | 0.0140 | (-0.0109, 0.0390) | 0.0298 | (-0.0088, 0.0683) | -0.0002 | (-0.0194, 0.0191) |
|  |  | 2015 | 0.0280 | (0.0113, 0.0447) | 0.0434 | (0.0217, 0.0651) | 0.0078 | (-0.0099, 0.0254) |
|  |  | 2016 | 0.0303 | (0.0091, 0.0515) | 0.0296 | (-0.0039, 0.0631) | 0.0271 | (0.0140, 0.0402) |
|  |  | 2017 | 0.0322 | (0.0079, 0.0565) | 0.0383 | (0.0118, 0.0647) | 0.0241 | (-0.0178, 0.0659) |
|  |  | 2018 | 0.0455 | (0.0322, 0.0588) | 0.0480 | (0.0248, 0.0712) | 0.0360 | (0.0043, 0.0677) |
|  |  | 2019 | 0.0571 | (0.0317, 0.0825) | 0.0600 | (0.0296, 0.0905) | 0.0580 | (0.0209, 0.0951) |
|  |  | 2020 | 0.0445 | (0.0038, 0.0851) | 0.0660 | (0.0300, 0.1020) | 0.0371 | (-0.0175, 0.0917) |
|  |  | 2021 | 0.0545 | (0.0340, 0.0750) | 0.0561 | (0.0377, 0.0746) | 0.0605 | (0.0065, 0.1145) |
|  |  | 2022 | 0.0655 | (0.0478, 0.0831) | 0.0812 | (0.0499, 0.1126) | 0.0480 | (0.0119, 0.0840) |
|  |  | 2023 | 0.0561 | (0.0345, 0.0777) | 0.0624 | (0.0402, 0.0845) | 0.0642 | (0.0201, 0.1084) |
|  | 2014 | 2006 | -0.0405 | (-0.0636, -0.0174) | -0.0166 | (-0.0443, 0.0111) | -0.0534 | (-0.0773, -0.0295) |
|  |  | 2007 | 0.0046 | (-0.0117, 0.0210) | 0.0368 | (0.0143, 0.0592) | -0.0122 | (-0.0313, 0.0069) |
|  |  | 2008 | -0.0223 | (-0.0401, -0.0044) | 0.0119 | (-0.0066, 0.0304) | -0.0660 | (-0.0851, -0.0470) |
|  |  | 2009 | 0.0095 | (-0.0059, 0.0250) | 0.0481 | (0.0289, 0.0672) | -0.0218 | (-0.0494, 0.0058) |
|  |  | 2010 | -0.0180 | (-0.0341, -0.0019) | 0.0238 | (0.0097, 0.0378) | -0.0449 | (-0.0684, -0.0214) |
|  |  | 2011 | -0.0078 | (-0.0195, 0.0039) | 0.0353 | (0.0158, 0.0547) | -0.0377 | (-0.0492, -0.0261) |
|  |  | 2012 | -0.0034 | (-0.0238, 0.0170) | 0.0198 | (-0.0042, 0.0438) | -0.0301 | (-0.0477, -0.0125) |
|  |  | 2014 | 0.0026 | (-0.0092, 0.0144) | 0.0273 | (0.0084, 0.0463) | -0.0292 | (-0.0502, -0.0082) |
|  |  | 2015 | 0.0105 | (-0.0048, 0.0257) | 0.0457 | (0.0248, 0.0665) | -0.0336 | (-0.0495, -0.0177) |
|  |  | 2016 | 0.0164 | (0.0033, 0.0295) | 0.0466 | (0.0199, 0.0734) | -0.0181 | (-0.0305, -0.0056) |
|  |  | 2017 | 0.0058 | (-0.005, 0.0165) | 0.0287 | (0.0099, 0.0474) | -0.0207 | (-0.039, -0.0023) |
|  |  | 2018 | 0.0165 | (0.0045, 0.0284) | 0.0432 | (0.0282, 0.0582) | -0.0179 | (-0.0365, 0.0007) |
|  |  | 2019 | 0.0339 | (0.0074, 0.0604) | 0.0365 | (0.0069, 0.0661) | 0.0423 | (0.0170, 0.0675) |
|  |  | 2020 | 0.0393 | (0.0189, 0.0598) | 0.0672 | (0.0454, 0.0889) | 0.0047 | (-0.0212, 0.0306) |
|  |  | 2021 | 0.0095 | (-0.0169, 0.0359) | 0.0241 | (-0.0014, 0.0495) | 0.0097 | (-0.0240, 0.0434) |
|  |  | 2022 | 0.0114 | (-0.0077, 0.0305) | 0.0272 | (0.0003, 0.0541) | 0.0074 | (-0.0114, 0.0262) |
|  |  | 2023 | 0.0164 | (-0.0046, 0.0373) | 0.0433 | (0.0215, 0.0652) | -0.0223 | (-0.0468, 0.0022) |
|  | 2020 | 2006 | -0.0274 | (-0.0557, 0.0009) | -0.0149 | (-0.0463, 0.0165) | -0.0047 | (-0.0336, 0.0241) |
|  |  | 2007 | -0.0116 | (-0.0376, 0.0143) | -0.0035 | (-0.0274, 0.0205) | 0.0123 | (-0.0238, 0.0483) |
|  |  | 2008 | -0.0257 | (-0.0522, 0.0007) | 0.0007 | (-0.0275, 0.0289) | -0.0325 | (-0.0621, -0.0030) |
|  |  | 2009 | -0.0107 | (-0.0432, 0.0218) | -0.0129 | (-0.0392, 0.0134) | 0.0182 | (-0.0298, 0.0662) |
|  |  | 2010 | -0.0114 | (-0.0458, 0.0231) | 0.0127 | (-0.0219, 0.0473) | -0.0075 | (-0.0496, 0.0345) |
|  |  | 2011 | 0.0032 | (-0.0260, 0.0324) | -0.0161 | (-0.0475, 0.0154) | 0.0389 | (0.0100, 0.0678) |
|  |  | 2012 | -0.0131 | (-0.0393, 0.0131) | -0.0199 | (-0.0500, 0.0101) | 0.0002 | (-0.0248, 0.0252) |
|  |  | 2013 | 0.0209 | (-0.0076, 0.0494) | 0.0103 | (-0.0212, 0.0419) | 0.0156 | (-0.0113, 0.0426) |
|  |  | 2014 | -0.0080 | (-0.0353, 0.0194) | -0.0173 | (-0.0477, 0.0132) | 0.0072 | (-0.0312, 0.0456) |
|  |  | 2015 | -0.0165 | (-0.0403, 0.0073) | -0.0133 | (-0.0506, 0.0239) | -0.0112 | (-0.0350, 0.0126) |
|  |  | 2016 | 0.0177 | (-0.0073, 0.0427) | 0.0217 | (-0.0025, 0.0460) | 0.0333 | (0.0024, 0.0642) |
|  |  | 2017 | 0.0158 | (-0.0089, 0.0405) | -0.0142 | (-0.0513, 0.0229) | 0.0459 | (0.0221, 0.0696) |
|  |  | 2018 | -0.0070 | (-0.0283, 0.0142) | -0.0045 | (-0.0326, 0.0236) | -0.0062 | (-0.0315, 0.0192) |
|  |  | 2020 | 0.0270 | (0.0041, 0.0499) | 0.0264 | (0.0019, 0.0509) | 0.0431 | (0.0182, 0.0680) |
|  |  | 2021 | 0.0144 | (-0.0107, 0.0395) | 0.0063 | (-0.0292, 0.0418) | 0.0317 | (-0.0057, 0.0691) |
|  |  | 2022 | -0.0066 | (-0.0246, 0.0113) | -0.0070 | (-0.0266, 0.0126) | 0.0009 | (-0.0290, 0.0308) |
|  |  | 2023 | -0.0118 | (-0.0308, 0.0072) | -0.0144 | (-0.0324, 0.0037) | 0.0047 | (-0.0301, 0.0394) |
|  | 2021 | 2006 | 0.0039 | (-0.0367, 0.0444) | 0.0030 | (-0.0375, 0.0434) | 0.0151 | (-0.0333, 0.0635) |
|  |  | 2007 | -0.0035 | (-0.0258, 0.0189) | -0.0079 | (-0.0335, 0.0177) | 0.0158 | (-0.0221, 0.0538) |
|  |  | 2008 | 0.0021 | (-0.0227, 0.0269) | 0.0041 | (-0.0223, 0.0306) | 0.0075 | (-0.0304, 0.0455) |
|  |  | 2009 | 0.0014 | (-0.0205, 0.0233) | -0.0191 | (-0.0519, 0.0137) | 0.0367 | (-0.0013, 0.0748) |
|  |  | 2010 | -0.0030 | (-0.0320, 0.0260) | -0.0142 | (-0.0505, 0.0222) | 0.0204 | (-0.0276, 0.0684) |
|  |  | 2011 | -0.0133 | (-0.0428, 0.0162) | -0.0114 | (-0.0361, 0.0133) | -0.0043 | (-0.0538, 0.0453) |
|  |  | 2012 | -0.0092 | (-0.0542, 0.0357) | -0.0263 | (-0.0522, -0.0004) | 0.0218 | (-0.0455, 0.0892) |
|  |  | 2013 | -0.0102 | (-0.0504, 0.0301) | -0.0035 | (-0.0422, 0.0352) | -0.0136 | (-0.0596, 0.0325) |
|  |  | 2014 | -0.0319 | (-0.0562, -0.0076) | -0.0511 | (-0.0881, -0.014) | -0.0058 | (-0.0465, 0.0349) |
|  |  | 2015 | -0.0336 | (-0.0621, -0.0051) | -0.0579 | (-0.0872, -0.0287) | -0.0069 | (-0.0484, 0.0346) |
|  |  | 2016 | -0.0391 | (-0.0659, -0.0122) | -0.0695 | (-0.1051, -0.034) | 0.0032 | (-0.0302, 0.0367) |
|  |  | 2017 | -0.0200 | (-0.0479, 0.0079) | -0.0379 | (-0.0767, 0.0008) | -0.0002 | (-0.0460, 0.0456) |
|  |  | 2018 | -0.0124 | (-0.0396, 0.0148) | -0.0324 | (-0.0543, -0.0106) | 0.0076 | (-0.0385, 0.0536) |
|  |  | 2019 | -0.0276 | (-0.0506, -0.0046) | -0.0482 | (-0.0764, -0.0200) | -0.0082 | (-0.0464, 0.0299) |
|  |  | 2021 | -0.0291 | (-0.0530, -0.0053) | -0.0211 | (-0.0455, 0.0034) | -0.0361 | (-0.0699, -0.0023) |
|  |  | 2022 | -0.0062 | (-0.0279, 0.0155) | -0.0237 | (-0.0606, 0.0132) | 0.0075 | (-0.0393, 0.0543) |
|  |  | 2023 | -0.0098 | (-0.0291, 0.0095) | -0.0248 | (-0.0435, -0.0062) | -0.0051 | (-0.0404, 0.0302) |
| Depressive mood | 2011 | 2006 | -0.0033 | (-0.0134, 0.0067) | 0.0197 | (-0.0119, 0.0513) | -0.0124 | (-0.0234, -0.0013) |
|  |  | 2007 | -0.0043 | (-0.0097, 0.0010) | -0.0102 | (-0.0460, 0.0257) | -0.0012 | (-0.0156, 0.0133) |
|  |  | 2008 | -0.0026 | (-0.0128, 0.0075) | -0.0090 | (-0.0390, 0.0211) | 0.0026 | (-0.0049, 0.0100) |
|  |  | 2009 | -0.0024 | (-0.0072, 0.0023) | -0.0031 | (-0.0329, 0.0267) | -0.0036 | (-0.0147, 0.0074) |
|  |  | 2011 | -0.0011 | (-0.0085, 0.0064) | -0.0069 | (-0.0414, 0.0275) | 0.0107 | (0.0023, 0.0191) |
|  |  | 2012 | -0.0020 | (-0.0122, 0.0082) | -0.0083 | (-0.0483, 0.0317) | 0.0038 | (-0.0108, 0.0184) |
|  |  | 2013 | -0.0007 | (-0.0126, 0.0113) | -0.0095 | (-0.0456, 0.0265) | 0.0028 | (-0.0128, 0.0184) |
|  |  | 2014 | 0.0014 | (-0.0106, 0.0134) | 0.0008 | (-0.0375, 0.0392) | 0.0057 | (-0.0075, 0.0188) |
|  |  | 2015 | 0.0038 | (-0.0099, 0.0175) | -0.0086 | (-0.0415, 0.0243) | 0.0142 | (0.0004, 0.0280) |
|  |  | 2016 | 0.0097 | (0.0017, 0.0178) | 0.0086 | (-0.0265, 0.0438) | 0.0152 | (0.0047, 0.0257) |
|  |  | 2017 | 0.0074 | (-0.0042, 0.019) | 0.0036 | (-0.0422, 0.0494) | 0.0201 | (0.0065, 0.0338) |
|  |  | 2018 | 0.0135 | (0.0066, 0.0204) | 0.0186 | (-0.0233, 0.0604) | 0.0214 | (0.0080, 0.0348) |
|  |  | 2019 | 0.0141 | (0.0035, 0.0246) | 0.0075 | (-0.0364, 0.0515) | 0.0271 | (0.0137, 0.0404) |
|  |  | 2020 | 0.0122 | (0.0033, 0.0210) | 0.0104 | (-0.0295, 0.0502) | 0.0219 | (0.0067, 0.0371) |
|  |  | 2021 | 0.0134 | (-0.0024, 0.0291) | 0.0254 | (-0.0330, 0.0839) | 0.0177 | (0.0069, 0.0286) |
|  |  | 2022 | 0.0035 | (-0.0103, 0.0174) | 0.0140 | (-0.0249, 0.0529) | 0.0136 | (-0.0012, 0.0283) |
|  |  | 2023 | 0.0025 | (-0.0231, 0.0281) | 0.0125 | (-0.0557, 0.0808) | 0.0170 | (-0.0023, 0.0362) |
|  | 2012 | 2006 | -0.0035 | (-0.0153, 0.0084) | 0.0051 | (-0.0347, 0.0449) | -0.0193 | (-0.0336, -0.005) |
|  |  | 2007 | -0.0018 | (-0.0148, 0.0111) | -0.0090 | (-0.0539, 0.0359) | 0.0053 | (-0.0124, 0.0230) |
|  |  | 2008 | -0.0058 | (-0.0222, 0.0106) | -0.0031 | (-0.0479, 0.0417) | -0.0096 | (-0.0235, 0.0042) |
|  |  | 2009 | 0.0050 | (-0.0067, 0.0168) | 0.0048 | (-0.0501, 0.0596) | 0.0009 | (-0.0174, 0.0192) |
|  |  | 2010 | 0.0031 | (-0.0073, 0.0135) | 0.0128 | (-0.0253, 0.0509) | -0.0091 | (-0.0199, 0.0017) |
|  |  | 2012 | -0.0105 | (-0.0234, 0.0023) | -0.0021 | (-0.0372, 0.0330) | -0.0171 | (-0.0337, -0.0005) |
|  |  | 2013 | 0.0089 | (-0.0047, 0.0226) | 0.0178 | (-0.0163, 0.0519) | 0.0009 | (-0.0192, 0.0211) |
|  |  | 2014 | 0.0028 | (-0.0223, 0.0278) | 0.0149 | (-0.0320, 0.0619) | 0.0020 | (-0.0313, 0.0353) |
|  |  | 2015 | 0.0100 | (-0.0064, 0.0264) | 0.0112 | (-0.0337, 0.0561) | 0.0132 | (-0.0048, 0.0313) |
|  |  | 2016 | 0.0096 | (-0.0047, 0.0239) | 0.0097 | (-0.0302, 0.0497) | 0.0132 | (-0.0038, 0.0302) |
|  |  | 2017 | 0.0016 | (-0.0187, 0.0220) | 0.0104 | (-0.0329, 0.0537) | 0.0025 | (-0.0319, 0.0369) |
|  |  | 2018 | 0.0055 | (-0.0075, 0.0186) | 0.0233 | (-0.0132, 0.0598) | -0.0036 | (-0.0226, 0.0154) |
|  |  | 2019 | -0.0039 | (-0.0232, 0.0153) | 0.0107 | (-0.0298, 0.0513) | -0.0176 | (-0.0520, 0.0168) |
|  |  | 2020 | -0.0079 | (-0.0196, 0.0038) | -0.0013 | (-0.0398, 0.0372) | -0.0132 | (-0.0245, -0.0019) |
|  |  | 2021 | -0.0071 | (-0.0205, 0.0063) | -0.0030 | (-0.0481, 0.0421) | -0.0047 | (-0.0221, 0.0127) |
|  |  | 2022 | -0.0039 | (-0.0184, 0.0106) | 0.0086 | (-0.0165, 0.0338) | -0.0089 | (-0.0315, 0.0137) |
|  |  | 2023 | -0.0019 | (-0.0296, 0.0259) | 0.0119 | (-0.0479, 0.0716) | -0.0075 | (-0.0407, 0.0258) |
|  | 2014 | 2006 | -0.0088 | (-0.0186, 0.0010) | -0.0560 | (-0.0863, -0.0257) | 0.0184 | (0.0046, 0.0323) |
|  |  | 2007 | 0.0176 | (0.0038, 0.0315) | 0.0332 | (-0.0044, 0.0708) | 0.0192 | (0.0022, 0.0362) |
|  |  | 2008 | 0.0075 | (-0.0067, 0.0218) | -0.0114 | (-0.0549, 0.0320) | 0.0204 | (0.0048, 0.0360) |
|  |  | 2009 | 0.0191 | (0.0058, 0.0324) | 0.0454 | (0.0138, 0.0770) | 0.0001 | (-0.0162, 0.0164) |
|  |  | 2010 | 0.0216 | (0.0096, 0.0337) | 0.0122 | (-0.025, 0.0495) | 0.0219 | (0.0057, 0.0381) |
|  |  | 2011 | 0.0024 | (-0.012, 0.0168) | -0.0103 | (-0.0466, 0.0260) | 0.0058 | (-0.0093, 0.0208) |
|  |  | 2012 | 0.0172 | (0.0070, 0.0275) | 0.0162 | (-0.0041, 0.0365) | 0.0127 | (-0.0057, 0.0311) |
|  |  | 2014 | 0.0036 | (-0.0081, 0.0153) | -0.0178 | (-0.0536, 0.0180) | 0.0105 | (-0.0031, 0.0242) |
|  |  | 2015 | 0.0258 | (0.0103, 0.0413) | 0.0040 | (-0.0392, 0.0472) | 0.0478 | (0.0301, 0.0654) |
|  |  | 2016 | 0.0226 | (0.0131, 0.0321) | 0.0082 | (-0.0259, 0.0423) | 0.0351 | (0.0176, 0.0526) |
|  |  | 2017 | 0.0226 | (0.0144, 0.0307) | 0.0338 | (-0.0027, 0.0703) | 0.0148 | (-0.0024, 0.0320) |
|  |  | 2018 | 0.0161 | (-0.0006, 0.0329) | -0.0114 | (-0.0542, 0.0314) | 0.0216 | (0.0035, 0.0396) |
|  |  | 2019 | 0.0288 | (0.0144, 0.0432) | 0.0305 | (-0.0074, 0.0683) | 0.0240 | (0.0047, 0.0433) |
|  |  | 2020 | 0.0198 | (0.0140, 0.0256) | 0.0266 | (-0.0071, 0.0603) | 0.0211 | (0.0125, 0.0298) |
|  |  | 2021 | 0.0090 | (-0.0119, 0.0300) | -0.0074 | (-0.0679, 0.0530) | 0.0246 | (0.0096, 0.0396) |
|  |  | 2022 | 0.0089 | (0.0004, 0.0173) | -0.0039 | (-0.0409, 0.0331) | 0.0201 | (0.0062, 0.0340) |
|  |  | 2023 | 0.0218 | (0.0032, 0.0404) | 0.0070 | (-0.0531, 0.067) | 0.0415 | (0.0240, 0.0590) |
|  | 2020 | 2006 | 0.0213 | (0.0064, 0.0362) | 0.0220 | (-0.0108, 0.0548) | 0.0240 | (0.0006, 0.0474) |
|  |  | 2007 | 0.0138 | (-0.0019, 0.0295) | 0.0264 | (-0.0201, 0.0729) | 0.0137 | (-0.0035, 0.0308) |
|  |  | 2008 | -0.0008 | (-0.0130, 0.0114) | -0.0115 | (-0.0676, 0.0445) | 0.0137 | (-0.0014, 0.0288) |
|  |  | 2009 | 0.0059 | (-0.0084, 0.0203) | -0.0071 | (-0.0526, 0.0385) | 0.0161 | (0.0010, 0.0312) |
|  |  | 2010 | 0.0102 | (-0.0011, 0.0215) | 0.0080 | (-0.0374, 0.0535) | 0.0108 | (-0.0036, 0.0251) |
|  |  | 2011 | 0.0092 | (-0.0015, 0.0198) | -0.0077 | (-0.0330, 0.0175) | 0.0124 | (-0.0047, 0.0295) |
|  |  | 2012 | 0.0197 | (0.0066, 0.0327) | 0.0387 | (0.0092, 0.0682) | 0.0154 | (-0.0019, 0.0328) |
|  |  | 2013 | 0.0423 | (0.0311, 0.0535) | 0.0372 | (0.0011, 0.0733) | 0.0611 | (0.0434, 0.0787) |
|  |  | 2014 | 0.0040 | (-0.0101, 0.0181) | -0.0062 | (-0.0590, 0.0466) | 0.0273 | (0.0123, 0.0424) |
|  |  | 2015 | 0.0123 | (0.0005, 0.0240) | 0.0117 | (-0.0302, 0.0536) | 0.0146 | (0.0013, 0.0279) |
|  |  | 2016 | 0.0172 | (0.0108, 0.0236) | 0.0278 | (-0.0025, 0.0581) | 0.0108 | (-0.0023, 0.0239) |
|  |  | 2017 | 0.0332 | (0.0203, 0.0462) | 0.0355 | (-0.0119, 0.0829) | 0.0325 | (0.0123, 0.0527) |
|  |  | 2018 | -0.0108 | (-0.0216, 0.0001) | -0.0217 | (-0.0673, 0.0239) | 0.0013 | (-0.0165, 0.0192) |
|  |  | 2020 | 0.0065 | (-0.0049, 0.0179) | -0.0066 | (-0.0365, 0.0232) | 0.0365 | (0.0146, 0.0584) |
|  |  | 2021 | 0.0341 | (0.0221, 0.0461) | 0.0397 | (-0.0022, 0.0816) | 0.0500 | (0.0408, 0.0592) |
|  |  | 2022 | 0.0050 | (-0.0071, 0.0171) | 0.0189 | (-0.0218, 0.0597) | 0.0037 | (-0.0106, 0.0181) |
|  |  | 2023 | 0.0168 | (-0.0003, 0.0338) | -0.0044 | (-0.0716, 0.0628) | 0.0385 | (0.0268, 0.0501) |
|  | 2021 | 2006 | -0.0006 | (-0.0140, 0.0128) | 0.0041 | (-0.0346, 0.0428) | 0.0031 | (-0.0254, 0.0316) |
|  |  | 2007 | -0.0222 | (-0.0385, -0.0059) | -0.0329 | (-0.0999, 0.0341) | -0.0149 | (-0.0320, 0.0023) |
|  |  | 2008 | -0.0144 | (-0.0291, 0.0003) | -0.0192 | (-0.0775, 0.0391) | -0.0032 | (-0.0286, 0.0222) |
|  |  | 2009 | 0.0005 | (-0.0173, 0.0183) | 0.0143 | (-0.0803, 0.1088) | 0.0016 | (-0.0210, 0.0241) |
|  |  | 2010 | -0.0105 | (-0.0204, -0.0007) | -0.0178 | (-0.0740, 0.0384) | -0.0004 | (-0.0163, 0.0155) |
|  |  | 2011 | 0.0031 | (-0.0140, 0.0202) | -0.0044 | (-0.0790, 0.0703) | 0.0056 | (-0.0077, 0.0190) |
|  |  | 2012 | 0.0041 | (-0.0065, 0.0147) | 0.0006 | (-0.0466, 0.0477) | 0.0115 | (-0.0246, 0.0477) |
|  |  | 2013 | -0.0071 | (-0.0204, 0.0062) | -0.0043 | (-0.0915, 0.0830) | 0.0016 | (-0.0163, 0.0196) |
|  |  | 2014 | 0.0001 | (-0.0196, 0.0198) | 0.0042 | (-0.0835, 0.0919) | 0.0061 | (-0.0099, 0.0220) |
|  |  | 2015 | -0.0098 | (-0.0288, 0.0091) | -0.0073 | (-0.0450, 0.0303) | 0.0031 | (-0.0118, 0.0180) |
|  |  | 2016 | -0.0036 | (-0.0126, 0.0055) | -0.0065 | (-0.0372, 0.0242) | 0.0137 | (-0.0050, 0.0324) |
|  |  | 2017 | -0.0122 | (-0.0222, -0.0023) | 0.0042 | (-0.0598, 0.0683) | -0.0185 | (-0.0414, 0.0043) |
|  |  | 2018 | -0.0058 | (-0.0171, 0.0055) | -0.0126 | (-0.0703, 0.0451) | 0.0135 | (-0.0137, 0.0407) |
|  |  | 2019 | -0.0024 | (-0.0166, 0.0117) | -0.0093 | (-0.0909, 0.0723) | 0.0052 | (-0.0403, 0.0506) |
|  |  | 2021 | -0.0073 | (-0.0293, 0.0147) | -0.0010 | (-0.0656, 0.0636) | -0.0045 | (-0.0471, 0.0380) |
|  |  | 2022 | -0.0007 | (-0.0208, 0.0194) | 0.0110 | (-0.0166, 0.0387) | -0.0041 | (-0.0246, 0.0163) |
|  |  | 2023 | 0.0061 | (-0.0132, 0.0254) | -0.0044 | (-0.0471, 0.0383) | 0.0188 | (-0.0003, 0.0379) |
| Suicide ideation | 2011 | 2006 | 0.0181 | (0.0096, 0.0267) | 0.0278 | (0.0138, 0.0418) | 0.0008 | (-0.0104, 0.0121) |
|  |  | 2007 | -0.0044 | (-0.0147, 0.0058) | -0.0093 | (-0.0223, 0.0036) | -0.0003 | (-0.0135, 0.0130) |
|  |  | 2008 | 0.0079 | (0.0031, 0.0127) | -0.0054 | (-0.0105, -0.0002) | 0.0211 | (0.0126, 0.0297) |
|  |  | 2009 | -0.0051 | (-0.0178, 0.0076) | -0.0008 | (-0.0133, 0.0117) | -0.0122 | (-0.0243, -0.0001) |
|  |  | 2011 | 0.0113 | (0.0052, 0.0174) | 0.0124 | (0.0044, 0.0203) | 0.0080 | (0.0004, 0.0157) |
|  |  | 2012 | -0.0020 | (-0.0182, 0.0142) | 0.0056 | (-0.0090, 0.0202) | -0.0077 | (-0.0283, 0.0128) |
|  |  | 2013 | 0.0003 | (-0.0136, 0.0142) | -0.0147 | (-0.0312, 0.0018) | 0.0131 | (-0.0009, 0.0271) |
|  |  | 2014 | 0.0035 | (-0.0073, 0.0143) | 0.0039 | (-0.0087, 0.0164) | 0.0025 | (-0.0104, 0.0155) |
|  |  | 2015 | -0.0021 | (-0.0125, 0.0083) | -0.0041 | (-0.0145, 0.0063) | 0.0038 | (-0.0106, 0.0181) |
|  |  | 2016 | 0.0073 | (-0.0054, 0.0200) | 0.0045 | (-0.0066, 0.0155) | 0.0098 | (-0.0109, 0.0306) |
|  |  | 2017 | 0.0069 | (-0.0049, 0.0187) | 0.0084 | (-0.0025, 0.0193) | 0.0038 | (-0.0130, 0.0206) |
|  |  | 2018 | 0.0158 | (0.0029, 0.0287) | 0.0086 | (-0.0046, 0.0218) | 0.0124 | (-0.0044, 0.0292) |
|  |  | 2019 | 0.0070 | (-0.0051, 0.0191) | 0.0080 | (-0.0017, 0.0176) | 0.0046 | (-0.0154, 0.0247) |
|  |  | 2020 | 0.0036 | (-0.0074, 0.0145) | 0.0131 | (0.0003, 0.0259) | -0.0052 | (-0.0232, 0.0129) |
|  |  | 2021 | 0.0073 | (-0.0054, 0.0201) | 0.0257 | (0.0137, 0.0377) | -0.0122 | (-0.0311, 0.0067) |
|  |  | 2022 | 0.0246 | (0.0089, 0.0402) | 0.0358 | (0.0203, 0.0514) | 0.0315 | (0.0078, 0.0551) |
|  |  | 2023 | 0.0256 | (0.0153, 0.0359) | 0.0308 | (0.0208, 0.0408) | 0.0236 | (0.0014, 0.0459) |
|  | 2012 | 2006 | -0.0004 | (-0.0187, 0.0180) | -0.0069 | (-0.0335, 0.0197) | 0.0064 | (-0.0129, 0.0257) |
|  |  | 2007 | -0.0041 | (-0.0236, 0.0154) | -0.0012 | (-0.0230, 0.0206) | -0.0108 | (-0.0353, 0.0138) |
|  |  | 2008 | -0.0007 | (-0.0087, 0.0074) | -0.0047 | (-0.0183, 0.0089) | 0.0032 | (-0.0034, 0.0098) |
|  |  | 2009 | -0.0074 | (-0.0263, 0.0115) | -0.0105 | (-0.0284, 0.0074) | -0.0051 | (-0.0225, 0.0124) |
|  |  | 2010 | 0.0010 | (-0.0092, 0.0111) | -0.0023 | (-0.0133, 0.0088) | 0.0012 | (-0.0134, 0.0157) |
|  |  | 2012 | -0.0041 | (-0.0226, 0.0145) | 0.0049 | (-0.0174, 0.0272) | -0.0122 | (-0.0311, 0.0066) |
|  |  | 2013 | 0.0090 | (-0.0029, 0.0208) | -0.0009 | (-0.0191, 0.0173) | 0.0199 | (0.0063, 0.0335) |
|  |  | 2014 | 0.0142 | (0.0050, 0.0235) | 0.0153 | (0.0007, 0.0299) | 0.0115 | (0.0005, 0.0226) |
|  |  | 2015 | 0.0073 | (-0.0051, 0.0198) | 0.0034 | (-0.0100, 0.0168) | 0.0112 | (-0.0055, 0.0279) |
|  |  | 2016 | 0.0059 | (-0.0067, 0.0184) | -0.0001 | (-0.0139, 0.0137) | 0.0093 | (-0.0059, 0.0245) |
|  |  | 2017 | 0.0101 | (-0.0070, 0.0272) | 0.0069 | (-0.0082, 0.0220) | 0.0105 | (-0.0134, 0.0345) |
|  |  | 2018 | 0.0111 | (-0.0012, 0.0234) | 0.0133 | (-0.0001, 0.0267) | 0.0096 | (-0.0065, 0.0257) |
|  |  | 2019 | -0.0059 | (-0.0235, 0.0116) | -0.0057 | (-0.0268, 0.0154) | -0.0091 | (-0.0235, 0.0052) |
|  |  | 2020 | -0.0092 | (-0.0221, 0.0037) | -0.0019 | (-0.0159, 0.0121) | -0.0166 | (-0.0421, 0.0088) |
|  |  | 2021 | -0.0104 | (-0.0255, 0.0047) | 0.0057 | (-0.0102, 0.0216) | -0.0319 | (-0.054, -0.0098) |
|  |  | 2022 | 0.0139 | (0.0007, 0.0272) | 0.0118 | (-0.0040, 0.0277) | 0.0136 | (0.0001, 0.0270) |
|  |  | 2023 | 0.0093 | (0.0003, 0.0184) | 0.0055 | (-0.0127, 0.0237) | 0.0095 | (-0.0057, 0.0247) |
|  | 2014 | 2006 | -0.0161 | (-0.0265, -0.0057) | -0.0398 | (-0.0549, -0.0247) | 0.0150 | (0.0008, 0.0291) |
|  |  | 2007 | 0.0146 | (0.0062, 0.0229) | 0.0368 | (0.0230, 0.0506) | -0.0101 | (-0.0276, 0.0074) |
|  |  | 2008 | -0.0088 | (-0.0212, 0.0037) | -0.0103 | (-0.0304, 0.0098) | -0.0060 | (-0.0190, 0.0070) |
|  |  | 2009 | 0.0160 | (0.0023, 0.0296) | 0.0129 | (-0.0069, 0.0327) | 0.0142 | (0.0020, 0.0263) |
|  |  | 2010 | -0.0072 | (-0.0221, 0.0078) | -0.0146 | (-0.0329, 0.0038) | 0.0036 | (-0.0109, 0.0180) |
|  |  | 2011 | -0.0068 | (-0.0190, 0.0055) | -0.0118 | (-0.0315, 0.0078) | -0.0004 | (-0.0123, 0.0115) |
|  |  | 2012 | 0.0019 | (-0.0110, 0.0147) | -0.0120 | (-0.0255, 0.0016) | 0.0132 | (-0.0071, 0.0334) |
|  |  | 2014 | -0.0003 | (-0.0105, 0.0098) | -0.0208 | (-0.0378, -0.0039) | 0.0246 | (0.0117, 0.0375) |
|  |  | 2015 | 0.0031 | (-0.0084, 0.0146) | 0.0006 | (-0.0166, 0.0178) | 0.0095 | (-0.0052, 0.0242) |
|  |  | 2016 | 0.0077 | (-0.0035, 0.0189) | -0.0031 | (-0.0164, 0.0101) | 0.0218 | (0.0029, 0.0407) |
|  |  | 2017 | 0.0115 | (-0.0005, 0.0235) | 0.0091 | (-0.0044, 0.0227) | 0.0188 | (0.0026, 0.0351) |
|  |  | 2018 | -0.0122 | (-0.0241, -0.0003) | -0.0338 | (-0.0477, -0.0199) | 0.0147 | (-0.001, 0.0304) |
|  |  | 2019 | 0.0134 | (-0.0008, 0.0276) | 0.0025 | (-0.0146, 0.0196) | 0.0237 | (0.0046, 0.0428) |
|  |  | 2020 | 0.0203 | (0.0080, 0.0326) | 0.0262 | (0.0093, 0.0431) | 0.0070 | (-0.0087, 0.0226) |
|  |  | 2021 | -0.0023 | (-0.0188, 0.0141) | -0.0003 | (-0.0181, 0.0176) | -0.0018 | (-0.0175, 0.0138) |
|  |  | 2022 | 0.0101 | (-0.0035, 0.0238) | 0.0058 | (-0.0140, 0.0256) | 0.0155 | (-0.0042, 0.0353) |
|  |  | 2023 | 0.0254 | (0.0103, 0.0405) | 0.0037 | (-0.0118, 0.0192) | 0.0484 | (0.0260, 0.0707) |
|  | 2020 | 2006 | 0.0152 | (-0.0018, 0.0321) | 0.0081 | (-0.0112, 0.0275) | 0.0193 | (0.0056, 0.0331) |
|  |  | 2007 | 0.0201 | (0.0096, 0.0307) | 0.0342 | (0.0177, 0.0507) | 0.0016 | (-0.0162, 0.0194) |
|  |  | 2008 | 0.0039 | (-0.0085, 0.0162) | 0.0125 | (-0.0055, 0.0305) | -0.0043 | (-0.0187, 0.0101) |
|  |  | 2009 | -0.0066 | (-0.0242, 0.0111) | 0.0091 | (-0.0079, 0.0262) | -0.0190 | (-0.0344, -0.0037) |
|  |  | 2010 | -0.0072 | (-0.0206, 0.0062) | 0.0046 | (-0.0069, 0.0161) | -0.0116 | (-0.0326, 0.0094) |
|  |  | 2011 | -0.0181 | (-0.0307, -0.0054) | -0.0204 | (-0.0346, -0.0061) | -0.0159 | (-0.0298, -0.0020) |
|  |  | 2012 | 0.0182 | (0.0038, 0.0326) | 0.0366 | (0.0260, 0.0472) | -0.0025 | (-0.0239, 0.0189) |
|  |  | 2013 | 0.0167 | (0.0022, 0.0312) | 0.0199 | (0.0030, 0.0368) | 0.0118 | (-0.0090, 0.0325) |
|  |  | 2014 | 0.0018 | (-0.0110, 0.0145) | 0.0196 | (0.0055, 0.0338) | -0.0166 | (-0.027, -0.0061) |
|  |  | 2015 | 0.0029 | (-0.0086, 0.0145) | 0.0059 | (-0.0046, 0.0165) | 0.0040 | (-0.0139, 0.0219) |
|  |  | 2016 | 0.0090 | (-0.0050, 0.0230) | 0.0106 | (-0.0072, 0.0285) | 0.0072 | (-0.0035, 0.0179) |
|  |  | 2017 | 0.0018 | (-0.0122, 0.0157) | -0.0017 | (-0.0156, 0.0123) | 0.0026 | (-0.0121, 0.0173) |
|  |  | 2018 | -0.0026 | (-0.0160, 0.0108) | -0.0009 | (-0.0164, 0.0146) | -0.0042 | (-0.0211, 0.0127) |
|  |  | 2020 | 0.0093 | (-0.0022, 0.0208) | 0.0210 | (0.0066, 0.0353) | -0.0099 | (-0.0262, 0.0064) |
|  |  | 2021 | 0.0213 | (0.0032, 0.0395) | 0.0387 | (0.0229, 0.0546) | -0.0015 | (-0.0184, 0.0153) |
|  |  | 2022 | 0.0160 | (0.0039, 0.0280) | 0.0204 | (0.0064, 0.0343) | 0.0131 | (0.0037, 0.0226) |
|  |  | 2023 | -0.0078 | (-0.0195, 0.0039) | -0.0100 | (-0.0264, 0.0065) | -0.0086 | (-0.0219, 0.0047) |
|  | 2021 | 2006 | 0.0189 | (-0.0050, 0.0428) | 0.0221 | (-0.0100, 0.0541) | 0.0172 | (-0.0110, 0.0453) |
|  |  | 2007 | -0.0029 | (-0.0175, 0.0117) | 0.0058 | (-0.0187, 0.0303) | -0.0089 | (-0.0312, 0.0135) |
|  |  | 2008 | 0.0060 | (-0.0133, 0.0252) | 0.0041 | (-0.0216, 0.0299) | 0.0094 | (-0.0135, 0.0322) |
|  |  | 2009 | 0.0131 | (-0.0110, 0.0371) | 0.0172 | (-0.0156, 0.0499) | 0.0060 | (-0.0152, 0.0272) |
|  |  | 2010 | 0.0039 | (-0.0168, 0.0245) | 0.0008 | (-0.0337, 0.0353) | 0.0056 | (-0.0148, 0.0261) |
|  |  | 2011 | 0.0062 | (-0.0130, 0.0255) | 0.0070 | (-0.0212, 0.0351) | 0.0100 | (-0.0118, 0.0318) |
|  |  | 2012 | 0.0064 | (-0.0114, 0.0242) | 0.0141 | (-0.0020, 0.0303) | -0.0015 | (-0.0329, 0.0299) |
|  |  | 2013 | 0.0141 | (-0.0053, 0.0335) | 0.0207 | (-0.0068, 0.0482) | 0.0118 | (-0.0090, 0.0326) |
|  |  | 2014 | 0.0066 | (-0.0127, 0.0258) | 0.0126 | (-0.0039, 0.0291) | 0.0057 | (-0.0187, 0.0302) |
|  |  | 2015 | -0.0023 | (-0.0205, 0.0159) | 0.0054 | (-0.0143, 0.0250) | -0.0078 | (-0.0278, 0.0122) |
|  |  | 2016 | 0.0122 | (-0.0139, 0.0383) | 0.0151 | (-0.0081, 0.0383) | 0.0106 | (-0.0272, 0.0484) |
|  |  | 2017 | 0.0161 | (-0.0073, 0.0394) | 0.0182 | (-0.0096, 0.0460) | 0.0151 | (-0.0078, 0.0380) |
|  |  | 2018 | 0.0162 | (-0.0021, 0.0345) | 0.0225 | (-0.0054, 0.0505) | 0.0072 | (-0.0200, 0.0344) |
|  |  | 2019 | 0.0082 | (-0.0141, 0.0304) | 0.0007 | (-0.0301, 0.0314) | 0.0148 | (-0.0018, 0.0315) |
|  |  | 2021 | 0.0104 | (-0.0006, 0.0214) | 0.0229 | (0.0006, 0.0452) | -0.0002 | (-0.0130, 0.0125) |
|  |  | 2022 | 0.0165 | (-0.0018, 0.0348) | 0.0218 | (-0.0022, 0.0457) | 0.0099 | (-0.0055, 0.0254) |
|  |  | 2023 | 0.0309 | (0.0105, 0.0513) | 0.0123 | (-0.0065, 0.0311) | 0.0495 | (0.0247, 0.0743) |
| Suicide attempt | 2011 | 2006 | 0.0030 | (-0.0017, 0.0076) | 0.0014 | (-0.0039, 0.0067) | 0.0097 | (0.0033, 0.0160) |
|  |  | 2007 | -0.0047 | (-0.0102, 0.0008) | -0.0095 | (-0.0144, -0.0046) | 0.0030 | (-0.0034, 0.0093) |
|  |  | 2008 | -0.0021 | (-0.0089, 0.0046) | -0.0097 | (-0.0162, -0.0031) | 0.0067 | (0.0022, 0.0112) |
|  |  | 2009 | -0.0056 | (-0.0110, -0.0003) | -0.0058 | (-0.0109, -0.0008) | -0.0043 | (-0.0111, 0.0026) |
|  |  | 2011 | -0.0008 | (-0.0050, 0.0034) | -0.0046 | (-0.0081, -0.0010) | 0.0060 | (0.0004, 0.0116) |
|  |  | 2012 | -0.0021 | (-0.0103, 0.0060) | -0.0001 | (-0.0062, 0.0061) | -0.0039 | (-0.0156, 0.0077) |
|  |  | 2013 | -0.0006 | (-0.0068, 0.0057) | -0.0056 | (-0.0129, 0.0017) | 0.0087 | (0.0015, 0.0159) |
|  |  | 2014 | 0.0010 | (-0.0030, 0.0049) | -0.0019 | (-0.0080, 0.0043) | 0.0015 | (-0.0083, 0.0114) |
|  |  | 2015 | -0.0072 | (-0.0106, -0.0038) | -0.0066 | (-0.0111, -0.0020) | -0.0033 | (-0.0134, 0.0069) |
|  |  | 2016 | -0.0013 | (-0.0057, 0.0031) | -0.0029 | (-0.0072, 0.0015) | 0.0011 | (-0.0031, 0.0054) |
|  |  | 2017 | -0.0043 | (-0.0090, 0.0005) | -0.0044 | (-0.0120, 0.0033) | -0.0016 | (-0.0090, 0.0058) |
|  |  | 2018 | 0.0011 | (-0.0043, 0.0064) | 0.0016 | (-0.0031, 0.0063) | 0.0061 | (-0.0063, 0.0184) |
|  |  | 2019 | 0.0005 | (-0.0084, 0.0094) | -0.0026 | (-0.0092, 0.0041) | 0.0010 | (-0.0099, 0.0118) |
|  |  | 2020 | -0.0018 | (-0.0072, 0.0037) | -0.0044 | (-0.0111, 0.0024) | -0.0034 | (-0.0118, 0.0049) |
|  |  | 2021 | -0.0080 | (-0.0149, -0.0012) | -0.0019 | (-0.0075, 0.0037) | -0.0076 | (-0.0206, 0.0055) |
|  |  | 2022 | 0.0033 | (-0.0012, 0.0078) | -0.0031 | (-0.0096, 0.0034) | 0.0036 | (-0.0061, 0.0133) |
|  |  | 2023 | 0.0019 | (-0.0061, 0.0098) | -0.0032 | (-0.0087, 0.0023) | 0.0049 | (-0.0085, 0.0183) |
|  | 2012 | 2006 | -0.0038 | (-0.0087, 0.0011) | -0.0040 | (-0.0134, 0.0055) | 0.0048 | (-0.0049, 0.0146) |
|  |  | 2007 | -0.0055 | (-0.0100, -0.0010) | -0.0012 | (-0.0103, 0.0079) | -0.0039 | (-0.0149, 0.0071) |
|  |  | 2008 | -0.0056 | (-0.0146, 0.0035) | -0.0045 | (-0.0107, 0.0017) | 0.0001 | (-0.0118, 0.0121) |
|  |  | 2009 | -0.0032 | (-0.0114, 0.0050) | -0.0022 | (-0.0103, 0.0059) | -0.0026 | (-0.0132, 0.0080) |
|  |  | 2010 | -0.0006 | (-0.0080, 0.0069) | 0.0006 | (-0.0071, 0.0084) | 0.0002 | (-0.0075, 0.0078) |
|  |  | 2012 | -0.0018 | (-0.0074, 0.0037) | 0.0012 | (-0.0076, 0.0100) | -0.0041 | (-0.0189, 0.0106) |
|  |  | 2013 | 0.0053 | (-0.0030, 0.0136) | 0.0046 | (-0.0051, 0.0144) | 0.0079 | (-0.0024, 0.0182) |
|  |  | 2014 | 0.0039 | (-0.0010, 0.0089) | 0.0027 | (-0.0069, 0.0124) | 0.0070 | (-0.0052, 0.0191) |
|  |  | 2015 | 0.0005 | (-0.0064, 0.0075) | 0.0019 | (-0.0022, 0.0060) | 0.0003 | (-0.0117, 0.0123) |
|  |  | 2016 | 0.0034 | (-0.0015, 0.0082) | 0.0051 | (-0.0006, 0.0109) | 0.0038 | (-0.0041, 0.0117) |
|  |  | 2017 | -0.0002 | (-0.0053, 0.0050) | 0.0023 | (-0.0065, 0.0111) | -0.0017 | (-0.0110, 0.0077) |
|  |  | 2018 | -0.0002 | (-0.0070, 0.0066) | 0.0062 | (-0.0033, 0.0156) | -0.0033 | (-0.0201, 0.0135) |
|  |  | 2019 | -0.0027 | (-0.0090, 0.0035) | -0.0004 | (-0.0095, 0.0086) | -0.0049 | (-0.0130, 0.0032) |
|  |  | 2020 | -0.0010 | (-0.0061, 0.0042) | 0.0033 | (-0.0076, 0.0141) | -0.0027 | (-0.0183, 0.0128) |
|  |  | 2021 | -0.0057 | (-0.0157, 0.0043) | 0.0018 | (-0.0018, 0.0055) | -0.0079 | (-0.0228, 0.0070) |
|  |  | 2022 | -0.0021 | (-0.0078, 0.0036) | -0.0004 | (-0.0085, 0.0077) | -0.0033 | (-0.0134, 0.0069) |
|  |  | 2023 | 0.0017 | (-0.0082, 0.0115) | 0.0003 | (-0.0088, 0.0095) | 0.0059 | (-0.0041, 0.0159) |
|  | 2014 | 2006 | -0.0050 | (-0.0095, -0.0004) | -0.0115 | (-0.0160, -0.0070) | -0.0028 | (-0.0120, 0.0064) |
|  |  | 2007 | 0.0043 | (-0.0012, 0.0098) | 0.0041 | (-0.0008, 0.0090) | -0.0015 | (-0.0139, 0.0110) |
|  |  | 2008 | 0.0000 | (-0.0061, 0.0060) | 0.0043 | (-0.0013, 0.0099) | -0.0053 | (-0.0156, 0.0050) |
|  |  | 2009 | -0.0082 | (-0.0168, 0.0004) | -0.0102 | (-0.0174, -0.0030) | -0.0146 | (-0.0273, -0.0018) |
|  |  | 2010 | -0.0033 | (-0.0102, 0.0035) | -0.0094 | (-0.0175, -0.0013) | -0.0032 | (-0.0117, 0.0053) |
|  |  | 2011 | -0.0023 | (-0.0069, 0.0023) | -0.0116 | (-0.0168, -0.0065) | 0.0062 | (-0.0025, 0.0148) |
|  |  | 2012 | 0.0057 | (-0.0007, 0.0122) | -0.0003 | (-0.005, 0.0043) | 0.0117 | (-0.0012, 0.0247) |
|  |  | 2014 | -0.0012 | (-0.0062, 0.0038) | -0.0119 | (-0.0165, -0.0073) | 0.0090 | (-0.0014, 0.0194) |
|  |  | 2015 | 0.0024 | (-0.0049, 0.0097) | -0.0036 | (-0.0075, 0.0004) | 0.0078 | (-0.0037, 0.0193) |
|  |  | 2016 | -0.0033 | (-0.0092, 0.0026) | -0.0087 | (-0.0134, -0.004) | 0.0028 | (-0.0065, 0.0121) |
|  |  | 2017 | -0.0050 | (-0.0097, -0.0003) | -0.0063 | (-0.0101, -0.0024) | -0.0054 | (-0.0130, 0.0023) |
|  |  | 2018 | -0.0068 | (-0.0144, 0.0008) | -0.0183 | (-0.0221, -0.0144) | 0.0091 | (-0.0030, 0.0212) |
|  |  | 2019 | 0.0027 | (-0.0055, 0.0109) | -0.0088 | (-0.0122, -0.0054) | 0.0147 | (0.0010, 0.0284) |
|  |  | 2020 | -0.0024 | (-0.0086, 0.0037) | -0.0023 | (-0.0057, 0.0011) | -0.0083 | (-0.0187, 0.0022) |
|  |  | 2021 | -0.0097 | (-0.0166, -0.0027) | -0.0109 | (-0.0151, -0.0068) | -0.0034 | (-0.0187, 0.0118) |
|  |  | 2022 | -0.0129 | (-0.0193, -0.0065) | -0.0161 | (-0.0228, -0.0094) | -0.0082 | (-0.0168, 0.0005) |
|  |  | 2023 | 0.0005 | (-0.0092, 0.0102) | -0.0063 | (-0.0125, -0.0001) | 0.0075 | (-0.0083, 0.0233) |
|  | 2020 | 2006 | 0.0071 | (0.0012, 0.0129) | 0.0142 | (0.0090, 0.0194) | 0.0116 | (-0.0012, 0.0244) |
|  |  | 2007 | -0.0016 | (-0.0094, 0.0063) | 0.0120 | (0.0062, 0.0177) | -0.0170 | (-0.0298, -0.0042) |
|  |  | 2008 | -0.0034 | (-0.0111, 0.0043) | 0.0076 | (0.0019, 0.0133) | -0.0126 | (-0.0242, -0.0011) |
|  |  | 2009 | -0.0068 | (-0.0185, 0.0050) | -0.0008 | (-0.0104, 0.0088) | -0.0112 | (-0.0248, 0.0024) |
|  |  | 2010 | -0.0032 | (-0.0133, 0.0068) | 0.0012 | (-0.0062, 0.0085) | -0.0058 | (-0.0190, 0.0075) |
|  |  | 2011 | 0.0006 | (-0.0061, 0.0073) | -0.0040 | (-0.0095, 0.0015) | 0.0025 | (-0.0066, 0.0117) |
|  |  | 2012 | 0.0002 | (-0.0093, 0.0097) | 0.0123 | (0.0074, 0.0171) | -0.0102 | (-0.0295, 0.0091) |
|  |  | 2013 | 0.0030 | (-0.0057, 0.0117) | 0.0041 | (0.0001, 0.0081) | 0.0064 | (-0.0100, 0.0228) |
|  |  | 2014 | 0.0003 | (-0.0084, 0.0090) | 0.0134 | (0.0105, 0.0163) | -0.0146 | (-0.0290, -0.0002) |
|  |  | 2015 | -0.0058 | (-0.0150 0.0034) | 0.0009 | (-0.0031, 0.0050) | -0.0134 | (-0.0257, -0.0011) |
|  |  | 2016 | -0.0023 | (-0.0108, 0.0062) | -0.0013 | (-0.0066, 0.0040) | -0.0033 | (-0.0150, 0.0084) |
|  |  | 2017 | -0.0004 | (-0.0070, 0.0063) | -0.0006 | (-0.0046, 0.0033) | 0.0023 | (-0.0123, 0.0170) |
|  |  | 2018 | -0.0001 | (-0.0110, 0.0108) | 0.0062 | (0.0021, 0.0103) | -0.0065 | (-0.0214, 0.0085) |
|  |  | 2020 | 0.0026 | (-0.0033, 0.0086) | 0.0158 | (0.0114, 0.0203) | -0.0138 | (-0.0230, -0.0047) |
|  |  | 2021 | 0.0036 | (-0.0006, 0.0077) | 0.0060 | (0.0022, 0.0098) | -0.0013 | (-0.0091, 0.0064) |
|  |  | 2022 | 0.0033 | (-0.0043, 0.0109) | 0.0099 | (0.0032, 0.0166) | -0.0083 | (-0.0189, 0.0024) |
|  |  | 2023 | -0.0011 | (-0.0090, 0.0068) | -0.0005 | (-0.0060, 0.0050) | -0.0062 | (-0.0178, 0.0053) |
|  | 2021 | 2006 | 0.0071 | (0.0019, 0.0124) | 0.0066 | (0.0011, 0.0121) | 0.0079 | (-0.0094, 0.0252) |
|  |  | 2007 | 0.0007 | (-0.0166, 0.0180) | 0.0034 | (-0.0002, 0.0070) | -0.0020 | (-0.0166, 0.0126) |
|  |  | 2008 | 0.0050 | (-0.0036, 0.0135) | -0.0008 | (-0.0059, 0.0042) | 0.0071 | (-0.0094, 0.0236) |
|  |  | 2009 | 0.0109 | (0.0014, 0.0203) | 0.0057 | (-0.0037, 0.0151) | 0.0133 | (0.0025, 0.0241) |
|  |  | 2010 | 0.0020 | (-0.0034, 0.0073) | -0.0005 | (-0.0084, 0.0074) | 0.0030 | (-0.0182, 0.0243) |
|  |  | 2011 | 0.0027 | (-0.0074, 0.0129) | -0.0021 | (-0.0078, 0.0036) | 0.0031 | (-0.0109, 0.0172) |
|  |  | 2012 | 0.0050 | (-0.0187, 0.0288) | 0.0082 | (0.0036, 0.0128) | -0.0023 | (-0.0362, 0.0317) |
|  |  | 2013 | 0.0056 | (-0.0120, 0.0231) | 0.0066 | (0.0041, 0.0091) | 0.0013 | (-0.0190, 0.0215) |
|  |  | 2014 | 0.0021 | (-0.0120, 0.0162) | 0.0047 | (-0.0029, 0.0122) | -0.0041 | (-0.0213, 0.0131) |
|  |  | 2015 | 0.0032 | (0.0003, 0.0062) | 0.0008 | (-0.0053, 0.0069) | 0.0013 | (-0.0135, 0.0161) |
|  |  | 2016 | 0.0060 | (-0.0107, 0.0227) | 0.0034 | (-0.0030, 0.0099) | 0.0022 | (-0.0124, 0.0167) |
|  |  | 2017 | 0.0080 | (-0.0059, 0.0219) | 0.0057 | (-0.0028, 0.0142) | 0.0087 | (-0.0108, 0.0282) |
|  |  | 2018 | 0.0063 | (-0.0023, 0.0150) | 0.0050 | (0.0015, 0.0085) | 0.0031 | (-0.0203, 0.0265) |
|  |  | 2019 | 0.0060 | (-0.0047, 0.0168) | 0.0036 | (-0.0024, 0.0097) | 0.0102 | (-0.0080, 0.0284) |
|  |  | 2021 | 0.0024 | (-0.0056, 0.0103) | 0.0093 | (0.0040, 0.0146) | -0.0046 | (-0.0290, 0.0199) |
|  |  | 2022 | 0.0046 | (-0.0038, 0.0131) | 0.0002 | (-0.0057, 0.0060) | 0.0079 | (-0.0107, 0.0265) |
|  |  | 2023 | 0.0083 | (-0.0098, 0.0264) | 0.0052 | (-0.0020, 0.0123) | 0.0086 | (-0.0083, 0.0254) |
