# Supplementary material for: Effects of student human rights ordinances on mental health among middle and high school students in South Korea: a difference-in-differences analysis
Source: Epidemiol Health. 2025 Mar 1;47:e2025011. doi: 10.4178/epih.e2025011 (PMC12062860; doi:10.4178/epih.e2025011)
Supplement: Supplementary Material 10. — Global effects of student human rights ordinances on mental health among middle and high school students in South Korea estimated using never-treated units as a comparison group [file epih-47-e2025011-Supplementary-10.docx]

Supplementary Material 10. Global effects of student human rights ordinances on mental health among middle and high school students in South Korea estimated using never-treated units as a comparison group

| Outcome | Total | | Male | | Female | |
| --- | --- | --- | --- | --- | --- | --- |
|  | Average treatment effect on the treated | 95% confidence interval | Average treatment effect on the treated | 95% confidence interval | Average treatment effect on the treated | 95% confidence interval |
| Perceived stress | 0.0046 | (-0.0023, 0.0116) | 0.0100 | (0.0025, 0.0174) | 0.0027 | (-0.0071, 0.0124) |
| Sleep insufficiency | 0.0037 | (-0.0285, 0.0360) | 0.0064 | (-0.0298, 0.0427) | -0.0029 | (-0.0367, 0.0309) |
| Depressive mood | 0.0045 | (-0.0013, 0.0104) | 0.0082 | (0.0007, 0.0157) | 0.0080 | (-0.0023, 0.0184) |
| Suicide ideation | 0.0082 | (0.0033, 0.0130) | 0.0097 | (0.0051, 0.0144) | 0.0062 | (-0.0013, 0.0138) |
| Suicide attempt | -0.0003 | (-0.0016, 0.0010) | 0.0001 | (-0.0047, 0.0049) | 0.0002 | (-0.0028, 0.0033) |

Note: The estimated effects represent weighted averages of the overall group-time average treatment effects, with weights being proportional to the size of each group.
