# Supplementary material for: Effects of student human rights ordinances on mental health among middle and high school students in South Korea: a difference-in-differences analysis
Source: Epidemiol Health. 2025 Mar 1;47:e2025011. doi: 10.4178/epih.e2025011 (PMC12062860; doi:10.4178/epih.e2025011)
Supplement: Supplementary Material 11. — Global effects of student human rights ordinances on mental health among middle and high school students in South Korea estimated without using the wild cluster bootstrap [file epih-47-e2025011-Supplementary-11.docx]

Supplementary Material 11. Global effects of student human rights ordinances on mental health among middle and high school students in South Korea estimated without using the wild cluster bootstrap

| Outcome | Total | | Male | | Female | |
| --- | --- | --- | --- | --- | --- | --- |
|  | Average treatment effect on the treated | 95% confidence interval | Average treatment effect on the treated | 95% confidence interval | Average treatment effect on the treated | 95% confidence interval |
| Perceived stress | 0.0023 | (-0.0054, 0.0101) | 0.0083 | (-0.0005, 0.0171) | -0.0014 | (-0.0123, 0.0096) |
| Sleep insufficiency | 0.0044 | (-0.0284, 0.0371) | 0.0078 | (-0.0313, 0.0468) | -0.0021 | (-0.0356, 0.0314) |
| Depressive mood | 0.0045 | (-0.0007, 0.0097) | 0.0068 | (-0.0005, 0.0140) | 0.0081 | (-0.0019, 0.0182) |
| Suicide ideation | 0.0069 | (0.0022, 0.0116) | 0.0077 | (0.0029, 0.0125) | 0.0058 | (-0.0010, 0.0127) |
| Suicide attempt | -0.0006 | (-0.0021, 0.0008) | -0.0008 | (-0.0053, 0.0037) | 0.0006 | (-0.0019, 0.0031) |

Note: The estimated effects represent weighted averages of the overall group-time average treatment effects, with weights being proportional to the size of each group.
