# Supplementary material for: Effects of student human rights ordinances on mental health among middle and high school students in South Korea: a difference-in-differences analysis
Source: Epidemiol Health. 2025 Mar 1;47:e2025011. doi: 10.4178/epih.e2025011 (PMC12062860; doi:10.4178/epih.e2025011)
Supplement: Supplementary Material 12. — Effects of student human rights ordinances on mental health among middle and high school students in South Korea estimated by two-way fixed effects regression [file epih-47-e2025011-Supplementary-12.docx]

Supplementary Material 12. Effects of student human rights ordinances on mental health among middle and high school students in South Korea estimated by two-way fixed effects regression

| Outcome | Total | | Male | | Female | |
| --- | --- | --- | --- | --- | --- | --- |
|  | Average treatment effect on the treated | 95% confidence interval | Average treatment effect on the treated | 95% confidence interval | Average treatment effect on the treated | 95% confidence interval |
| Perceived stress | -0.0013 | (-0.0098, 0.0073) | 0.0007 | (-0.0091, 0.0106) | -0.0006 | (-0.0126, 0.0115) |
| Sleep insufficiency | 0.0019 | (-0.0296, 0.0334) | 0.0021 | (-0.0307, 0.0349) | 0.0016 | (-0.0291, 0.0323) |
| Depressive mood | 0.0063 | (0.0016, 0.0110) | 0.0058 | (-0.0011, 0.0126) | 0.0112 | (0.0035, 0.0188) |
| Suicide ideation | 0.0057 | (0.0018, 0.0097) | 0.0059 | (0.0021, 0.0096) | 0.0059 | (0.0008, 0.0110) |
| Suicide attempt | 0.0011 | (0.0001, 0.0022) | 0.0020 | (-0.0002, 0.0042) | 0.0009 | (-0.0013, 0.0031) |
