# Supplementary material for: Effects of student human rights ordinances on mental health among middle and high school students in South Korea: a difference-in-differences analysis
Source: Epidemiol Health. 2025 Mar 1;47:e2025011. doi: 10.4178/epih.e2025011 (PMC12062860; doi:10.4178/epih.e2025011)
Supplement: Supplementary Material 13. — Robust confidence intervals for the overall dynamic effects of student human rights ordinances by type and degree of potential violations of parallel trends [file epih-47-e2025011-Supplementary-13.docx]

Supplementary Material 13. Robust confidence intervals for the overall dynamic effects of student human rights ordinances by type and degree of potential violations of parallel trends

| Outcome | Restriction type | Mbar and M | Total | | Male | | Female | |
| --- | --- | --- | --- | --- | --- | --- | --- | --- |
|  |  |  | Lower bound | Upper bound | Lower bound | Upper bound | Lower bound | Upper bound |
| Perceived stress |  | Original | -0.0042 | 0.0119 | 0.0014 | 0.0192 | -0.0115 | 0.0116 |
|  | Relative magnitudes | 0.5 | -0.0210 | 0.0290 | -0.0170 | 0.0370 | -0.0310 | 0.0310 |
|  |  | 1.0 | -0.0450 | 0.0531 | -0.0410 | 0.0611 | -0.0591 | 0.0591 |
|  |  | 1.5 | -0.0691 | 0.0771 | -0.0651 | 0.0851 | -0.0871 | 0.0871 |
|  |  | 2.0 | -0.0931 | 0.1011 | -0.0911 | 0.1111 | -0.1151 | 0.1151 |
|  | Smoothness | 0.00 | -0.0037 | 0.0124 | 0.0018 | 0.0196 | -0.0118 | 0.0114 |
|  |  | 0.01 | -0.0129 | 0.0222 | -0.0126 | 0.0269 | -0.0156 | 0.0260 |
|  |  | 0.02 | -0.0229 | 0.0322 | -0.0226 | 0.0369 | -0.0256 | 0.0360 |
|  |  | 0.03 | -0.0329 | 0.0422 | -0.0326 | 0.0469 | -0.0356 | 0.0460 |
|  |  | 0.04 | -0.0429 | 0.0522 | -0.0426 | 0.0569 | -0.0456 | 0.0560 |
|  |  | 0.05 | -0.0529 | 0.0622 | -0.0526 | 0.0669 | -0.0556 | 0.0660 |
| Sleep insufficiency |  | Original | -0.0157 | 0.0272 | -0.0159 | 0.0342 | -0.0235 | 0.0216 |
|  | Relative magnitudes | 0.5 | -0.0250 | 0.0370 | -0.0270 | 0.0450 | -0.0531 | 0.0511 |
|  |  | 1.0 | -0.0450 | 0.0551 | -0.0511 | 0.0691 | -0.0931 | 0.0911 |
|  |  | 1.5 | -0.0651 | 0.0771 | -0.0751 | 0.0931 | -0.1351 | 0.1331 |
|  |  | 2.0 | -0.0871 | 0.0991 | -0.1011 | 0.1211 | -0.1792 | 0.1772 |
|  | Smoothness | 0.00 | -0.0166 | 0.0263 | -0.0166 | 0.0335 | -0.0235 | 0.0216 |
|  |  | 0.01 | -0.0196 | 0.0402 | -0.0226 | 0.0454 | -0.0322 | 0.0329 |
|  |  | 0.02 | -0.0296 | 0.0502 | -0.0317 | 0.0561 | -0.0386 | 0.0471 |
|  |  | 0.03 | -0.0396 | 0.0602 | -0.0417 | 0.0661 | -0.0486 | 0.0571 |
|  |  | 0.04 | -0.0496 | 0.0702 | -0.0517 | 0.0761 | -0.0586 | 0.0671 |
|  |  | 0.05 | -0.0596 | 0.0802 | -0.0617 | 0.0861 | -0.0686 | 0.0771 |
| Depressive mood |  | Original | -0.0013 | 0.0104 | 0.0001 | 0.0151 | -0.0025 | 0.0197 |
|  | Relative magnitudes | 0.5 | -0.0150 | 0.0230 | -0.0230 | 0.0370 | -0.0190 | 0.0370 |
|  |  | 1.0 | -0.0330 | 0.0410 | -0.0511 | 0.0671 | -0.0430 | 0.0611 |
|  |  | 1.5 | -0.0511 | 0.0591 | -0.0811 | 0.0971 | -0.0691 | 0.0851 |
|  |  | 2.0 | -0.0691 | 0.0791 | -0.1111 | 0.1251 | -0.0931 | 0.1111 |
|  | Smoothness | 0.00 | -0.0012 | 0.0105 | 0.0001 | 0.0151 | -0.0018 | 0.0203 |
|  |  | 0.01 | -0.0108 | 0.0210 | -0.0120 | 0.0296 | -0.0164 | 0.0265 |
|  |  | 0.02 | -0.0208 | 0.0310 | -0.0214 | 0.0417 | -0.0264 | 0.0365 |
|  |  | 0.03 | -0.0308 | 0.0410 | -0.0314 | 0.0517 | -0.0364 | 0.0465 |
|  |  | 0.04 | -0.0408 | 0.0510 | -0.0414 | 0.0617 | -0.0464 | 0.0565 |
|  |  | 0.05 | -0.0508 | 0.0610 | -0.0514 | 0.0717 | -0.0564 | 0.0665 |
| Suicide ideation |  | Original | 0.0031 | 0.0127 | 0.0048 | 0.0136 | -0.0011 | 0.0140 |
|  | Relative magnitudes | 0.5 | -0.0090 | 0.0230 | -0.0150 | 0.0330 | -0.0130 | 0.0270 |
|  |  | 1.0 | -0.0230 | 0.0390 | -0.0390 | 0.0571 | -0.0310 | 0.0450 |
|  |  | 1.5 | -0.0390 | 0.0551 | -0.0631 | 0.0811 | -0.0511 | 0.0631 |
|  |  | 2.0 | -0.0551 | 0.0711 | -0.0871 | 0.1071 | -0.0691 | 0.0831 |
|  | Smoothness | 0.00 | 0.0038 | 0.0134 | 0.0056 | 0.0144 | -0.0006 | 0.0145 |
|  |  | 0.01 | -0.0090 | 0.0231 | -0.0088 | 0.0232 | -0.0149 | 0.0237 |
|  |  | 0.02 | -0.0190 | 0.0331 | -0.0188 | 0.0332 | -0.0249 | 0.0336 |
|  |  | 0.03 | -0.0290 | 0.0431 | -0.0288 | 0.0432 | -0.0349 | 0.0436 |
|  |  | 0.04 | -0.0390 | 0.0531 | -0.0388 | 0.0532 | -0.0449 | 0.0536 |
|  |  | 0.05 | -0.0490 | 0.0631 | -0.0488 | 0.0632 | -0.0549 | 0.0636 |
| Suicide attempt |  | Original | -0.0021 | 0.0007 | -0.0052 | 0.0033 | -0.0022 | 0.0033 |
|  | Relative magnitudes | 0.5 | -0.0070 | 0.0050 | -0.0070 | 0.0050 | -0.0090 | 0.0110 |
|  |  | 1.0 | -0.0150 | 0.0130 | -0.0130 | 0.0110 | -0.0210 | 0.0210 |
|  |  | 1.5 | -0.0230 | 0.0210 | -0.0190 | 0.0170 | -0.0310 | 0.0330 |
|  |  | 2.0 | -0.0290 | 0.0290 | -0.0250 | 0.0230 | -0.0430 | 0.0430 |
|  | Smoothness | 0.00 | -0.0024 | 0.0004 | -0.0050 | 0.0035 | -0.0020 | 0.0034 |
|  |  | 0.01 | -0.0158 | 0.0116 | -0.0172 | 0.0126 | -0.0132 | 0.0140 |
|  |  | 0.02 | -0.0258 | 0.0216 | -0.0272 | 0.0226 | -0.0232 | 0.0240 |
|  |  | 0.03 | -0.0358 | 0.0316 | -0.0372 | 0.0326 | -0.0332 | 0.0340 |
|  |  | 0.04 | -0.0458 | 0.0416 | -0.0472 | 0.0426 | -0.0432 | 0.0440 |
|  |  | 0.05 | -0.0558 | 0.0516 | -0.0572 | 0.0526 | -0.0532 | 0.0540 |
